# Genetic evidence for distinct biological mechanisms that link adiposity to type 2 diabetes: towards precision medicine

**DOI:** 10.2337/db23-1005

**Published:** 2024-03-26

**Authors:** Angela Abraham, Madeleine Cule, Marjola Thanaj, Nicolas Basty, M. Amin Hashemloo, Elena P. Sorokin, Brandon Whitcher, Stephen Burgess, Jimmy D. Bell, Naveed Sattar, E. Louise Thomas, Hanieh Yaghootkar

**Affiliations:** 1 College of Health and Science, University of Lincoln, Joseph Banks Laboratories, Green Lane, Lincoln, UK; 2 Calico Life Sciences LLC, South San Francisco, CA; 3 Research Centre for Optimal Health, School of Life Sciences, University of Westminster, London, UK; 4 Department of Life Sciences, Brunel University London, Uxbridge, United Kingdom; 5 Department of Radiology, MRI Unit, The Royal Marsden NHS Foundation Trust, London, UK; 6 MRC Biostatistics Unit, University of Cambridge, Cambridge, UK; 7 School of Cardiovascular and Metabolic Health, University of Glasgow, Glasgow, UK

**Keywords:** unfavorable adiposity, favorable adiposity, type 2 diabetes, clustering, MRI, metabolites, ectopic fat, precision medicine

## Abstract

We aimed to unravel the mechanisms connecting adiposity to type 2 diabetes. We employed MR-Clust to cluster independent genetic variants associated with body fat percentage (388 variants) and BMI (540 variants) based on their impact on type 2 diabetes. We identified five clusters of adiposity-increasing alleles associated with higher type 2 diabetes risk (unfavorable adiposity) and three clusters associated with lower risk (favorable adiposity). We then characterized each cluster based on various biomarkers, metabolites and Magnetic Resonance Imaging-based measures of fat distribution and muscle quality. Analyzing the metabolic signatures of these clusters revealed two primary mechanisms connecting higher adiposity to reduced type 2 diabetes risk. The first involves higher adiposity in subcutaneous tissues (abdomen and thigh), lower liver fat, improved insulin sensitivity, and decreased risk of cardiometabolic diseases and diabetes complications. The second mechanism is characterized by increased body size, enhanced muscle quality, with no impact on cardiometabolic outcomes. Furthermore, our findings unveil diverse mechanisms linking higher adiposity to higher disease risk, such as cholesterol pathways or inflammation. These results reinforce the existence of adiposity-related mechanisms that may act as protective factors against type 2 diabetes and its complications, especially when accompanied by reduced ectopic liver fat.

## Introduction

The strong link between excess weight (adiposity) and type 2 diabetes emphasizes weight management’s crucial role in prevention and treatment (1). However, the complex nature of type 2 diabetes and adiposity, influenced by genetics and lifestyle, poses challenges. This complexity leads to variations in insulin resistance, production, and fat accumulation in ectopic places (liver, skeletal muscles and pancreas) (2), making tailored weight management for diabetes challenging (3,4). While weight loss benefits glycemic control and health, responses vary among individuals (5,6), underscoring the need for personalized interventions.

Individuals with the same overall adiposity also have different risks of developing cardiometabolic disease (7,8). Reporting adiposity using surrogates like BMI has limitations in distinguishing fat and lean mass or accounting for variations in fat distribution, for example between the metabolically benign subcutaneous fat or more metabolically harmful visceral fat, and across different ethnicities (9,10). The current strategy for managing obesity in individuals with type 2 diabetes relies on using crude cut-offs for BMI and metabolic measures such as HbA1c or blood pressure. There is a need to create a reliable subtype classification system that accounts for the underlying causal factors that connect adiposity and type 2 diabetes to allow more accurate predictions of the benefits of intentional weight loss.

Research on adiposity subtype classification has primarily focused on metabolically healthy obesity, a condition with multiple definitions where individuals with obesity may not immediately exhibit metabolic dysfunction (11,12). Other approaches have involved behavioral traits, BMI, HbA1c, cardiometabolic traits and machine-learning techniques (13,14). However, these studies often relied on traits secondary to obesity or diabetes, introducing potential confounding from correlated factors and limiting their biological or clinical significance. In contrast, approaches that integrate genetic data allow clustering based on risk factors present at birth and unaffected by treatment, distinct from clinical biomarkers. In our previous work, we combined genetics with machine learning to identify two adiposity phenotypes with opposing effects on type 2 diabetes risk (15). Yet, including metabolic biomarkers, like liver-specific enzymes, in our model might introduce circular arguments, potentially biasing findings toward specific aspects, such as variants influencing liver fat.

In this study, we hypothesized that distinct biological pathways link higher adiposity with type 2 diabetes risk. We first selected variants associated with measures of adiposity. We next employed MR-Clust (16) to categorize adiposity variants based on their causal links to type 2 diabetes. MR-Clust groups variants with similar effect estimates, operating on the premise that an exposure (e.g., adiposity) can impact an outcome (e.g., type 2 diabetes) through diverse causal mechanisms with varying degrees. MR-Clust includes a provision to address potential spurious clusters by classifying variants with uncertain causal effect estimates into either ‘null’ or ‘junk’ clusters. This methodology has been previously applied to cluster IGF-1 associated variants based on their causal associations with type 2 diabetes (17). We then used different biomarkers, including metabolites, lipids, insulin sensitivity and secretion measures, and inflammatory cytokines to characterize metabolic signatures of each cluster. To further investigate the difference between clusters, we quantified the genetic effect of each cluster on body composition and adipose tissue distribution measured using magnetic resonance imaging (MRI). Finally, we estimated the causal effect of higher adiposity through each cluster on different diseases, including those common in people with type 2 diabetes, using Mendelian Randomization.

## Research Design and Methods

### Study design

Figure S1 summarizes our study design. To identify distinct causal pathways that link adiposity to type 2 diabetes, we first used independent genetic variants associated with two measures of adiposity − body fat percentage (BFP) and BMI. Although BMI does not represent adiposity accurately (9), it is by far the most commonly utilized metric to categorize people with obesity, therefore it is a useful measure to compare with body fat percentage (BFP). Second, we clustered these genetic variants based on their effect on type 2 diabetes risk (18). Each cluster represents a different causal pathway from adiposity to type 2 diabetes risk. Third, we validated the effect of each cluster on type 2 diabetes risk using FinnGen (Data Freeze 8 (19)) as an independent cohort. Fourth, to find the metabolic signature of each cluster, we calculated cluster-specific genetic risk score (GRS) effects on different biomarkers. Fifth, we calculated the causal effect of higher adiposity using Mendelian Randomization (MR) through each cluster on different diseases, including those prevalent in type 2 diabetes.

### Identification of distinct causal pathways

To identify distinct causal pathways linking adiposity to type 2 diabetes, we employed MR-Clust (16). This method calculates the Mendelian randomization estimate for each genetic variant as the ratio of the genetic association with the outcome (type 2 diabetes) divided by the genetic association with the exposure measure (adiposity) and seeks to find clusters of variants with similar estimates by maximizing the likelihood of a mixture of normal distributions. By convention, a genetic variant is only assigned to a cluster if the estimated probability of cluster membership is greater than 80%; if lower than this, then the variant is not assigned to any cluster. The motivation is that variants with similar Mendelian randomization estimates are likely to influence the outcome via similar mechanisms.

### Data source

We used published Genome-Wide Association Studies (GWAS) summary statistics from the largest and latest studies for traits of interest (anthropometric traits, clinical biomarkers, insulin sensitivity and secretion measures, metabolites and inflammatory markers and cytokines), focusing on European-specific data (table 1). For measures of adiposity, we accessed the GWAS of BFP from the IEU OpenGWAS project (20), where BFP had been estimated by impedance measurement in the UK Biobank (21), using the R package *ieugwasr* (n = 454,633). For BMI, we used the latest meta-analysis of the GIANT consortium and UK Biobank (n = 806,834) (22). To determine adiposity variant clusters, we used European-specific data from the DIAMANTE type 2 diabetes GWAS (80154 cases vs. 853816 controls) (18). For the second type 2 diabetes dataset and disease outcomes, we used data from FinnGen Data Freeze 8 or 7 (19).

### Studies of MRI scans

The UK Biobank MRI abdominal protocol has previously been reported (23). We used the neck-to-knee Dixon MRI and single-slice multiecho MRI in the abdomen. Dedicated image processing using deep learning models trained on 100+ manually annotated structures, achieved DICE scores > 0.8 for each organ (24–27). Image-derived phenotypes (IDPs) from these segmentations include volume, and median proton density fat fraction (PDFF), which was calculated from the Phase Regularized Estimation using Smoothing and Constrained Optimization (PRESCO) method (28). Quality control involved evaluating univariate distributions and visually inspecting scans with extreme values.

Table S1 summarizes the 15 IDPs used in this study including: subcutaneous adipose tissue (SAT) volumes (abdominal and thigh), visceral adipose tissue (VAT) volumes, internal fat and thigh intermuscular adipose tissue volumes (corrected for muscle volume), iliopsoas and total muscle volumes (indexed to height^2^), and organ volumes (kidney, pancreas, liver and spleen). We computed VAT:ASAT ratio. We also obtained a measure of fat (PDFF) stored in the liver, pancreas and the paraspinal muscles (intramyocellular fat), from the single-slice multiecho acquisition.

GWAS for the IDPs were performed using REGENIE version v3.1.1 (29). We included participants self-identified as ‘White British’ and clustering as such in PCA, excluding anomalies related to sex, heterozygosity, missingness, and genotype call rate (21). Sample sizes ranged from 28,587 to 37,589. Age, age^2^, sex, genotyping array, imaging center, and the first 10 principal components of the genotype relatedness matrix were included. Phenotypes were inverse normal transformed. Imputed SNPs were filtered to MAF > 0.01 and INFO score > 0.9, leaving 9,788,243 SNPs included in the final association study.

### Genetic risk score analysis

To calculate genetic risk score effects, we extracted effect size estimates (beta) and its corresponding standard error (SE) for each variant from trait GWAS summary statistics. For missing variants, we obtained proxies (r^2^ ≥ 0.8) using the European reference panel from the 1000 Genomes Project Phase 3 (1000G EUR). We aligned all effects for the adiposity increasing alleles. We performed a random-effect meta-analyses approach using the ‘rma’ function in the R package *metafor* to calculate the effect of each genetic risk score as previously described (30). To account for multiple testing, we used Benjamini-Hochberg−adjusted p-value < 0.05 to highlight significant associations.

### Mendelian randomization (MR) analysis

To best estimate the causal effects of each cluster on disease outcomes, we performed MR analyses in R (version 4.2.2) using the *TwoSampleMR* package (31,32). The Inverse Variance Weighted method (IVW) was our main test. We used MR-Egger as a sensitivity analysis method to identify horizontal pleiotropy based on the Egger intercept. Additionally, we utilized weighted median, simple mode, and weighted mode (33). For missing variants, we calculated proxies (r^2^ ≥ 0.8) using the European reference panel from the 1000 Genomes Project Phase 3 (1000G EUR). To account for multiple testing, we used a Benjamini-Hochberg–adjusted p-value < 0.05 to highlight significant causal associations.

### Pathway enrichment analysis

For each cluster, we first used the SNP2GENE function in FUMA (34) to identify expression quantitative trait loci (eQTL) using GTEx (35) v8 and default settings. Genes identified through SNP2GENE were input into the PANTHER v.17.0 tool for pathway enrichment analysis (36).

### eQTL comparison in adipose and brain tissue

To compare the number of independent eQTLs within each cluster in subcutaneous adipose, visceral adipose and brain tissue, eQTLs were identified using FUMA and then clumped using the European reference panel from the 1000 Genomes Project Phase 3 (1000G EUR), using a moderate cut of r^2^ ≥ 0.1 within 10,000 kb windows. Data sources for tissues were MuTHER and GTEx v8.

## Results

### Clusters of adiposity genetic variants

The adiposity-increasing alleles had a considerable heterogeneous effect on type 2 diabetes risk (figure S2). There was also significant heterogeneity in causal effects from MR-IVW results among instruments for both BFP and BMI (Cochran’s Q statistic p-value < 1e^-150^ and 1.29e^-140^, respectively), suggesting that distinct causal pathways exist between adiposity and type 2 diabetes.

**Figure 1:**
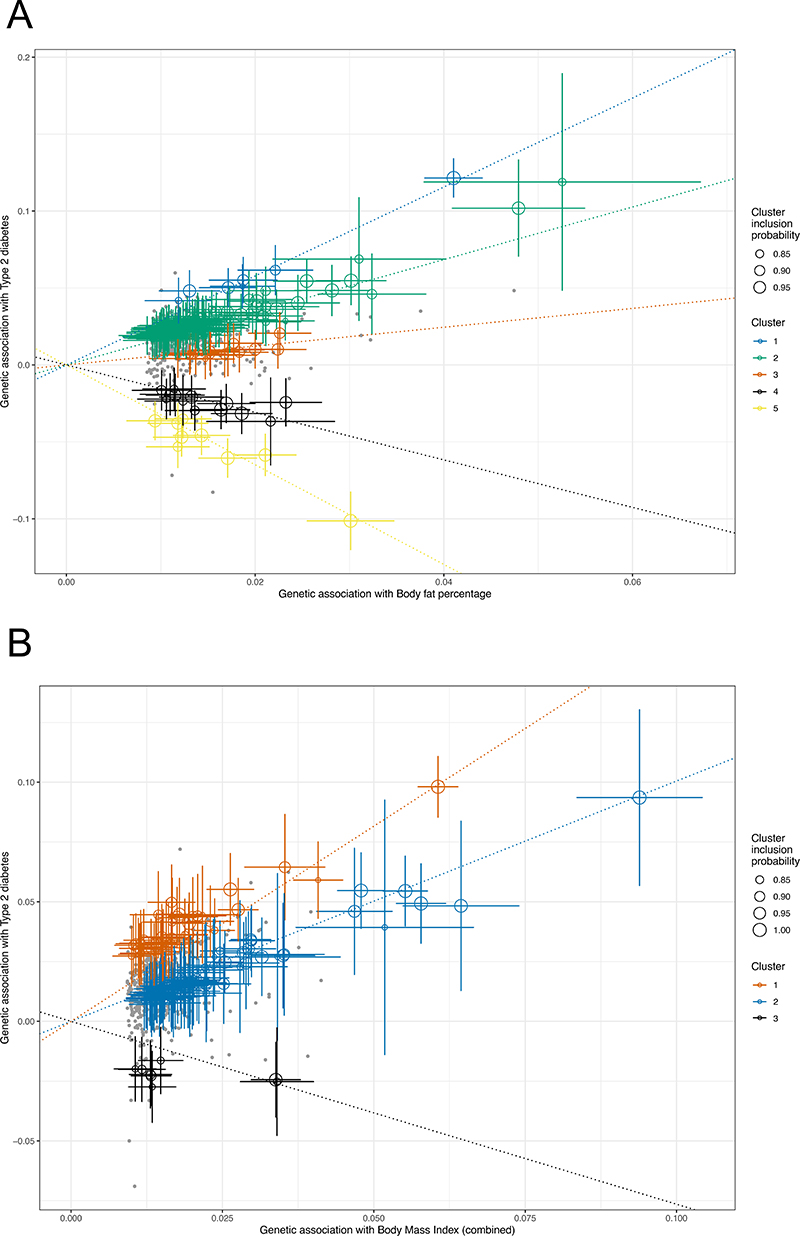
Scatter plots of the genetic associations with type 2 diabetes per additional adiposity-increasing allele using (A) body fat percentage and (B) BMI. Each circle represents a genetic variant. Error bars represent 95% confidence intervals for the genetic associations. Colors represent the clusters and lines represent the estimated causal effect of each cluster on type 2 diabetes through increasing adiposity. Only variants with a probability of >= 80% for belonging to one of the clusters are included in the plot and taken forward for further analysis. Variants with uncertain cluster membership are displayed as grey dots.

Using MR-Clust, we identified five clusters of BFP-increasing alleles representing five different causal pathways (figure 1A). Three clusters, comprising 7 variants in BFP-C1, 101 in BFP-C2, and 14 in BFP-C3, indicated a positive causal effect on type 2 diabetes risk, aligning with ‘unfavorable adiposity’ (higher adiposity, adverse metabolic profile, higher disease risk (15); table S2). Conversely, two BFP clusters (BFP-C4 with 13 variants and BFP-C5 with 9 variants) suggested a strong negative causal effect, consistent with ‘favorable adiposity’ (higher adiposity, favorable metabolic profile, lower disease risk (15)). Among BFP-C1, BFP-C2 and BFP-C3, 2, 5, and 3 variants, respectively, were previously associated with unfavorable adiposity (15). Among BFP-C4 and BFP-C5, 4 variants in each cluster were previously associated with favorable adiposity (table 2) (15). The higher number of previously known favorable and unfavorable adiposity variants among BFP clusters is anticipated, as the earlier study exclusively utilized variants associated with BFP to identify these groups.

We also identified 3 clusters of BMI-increasing alleles (figure 1B). Two clusters (BMI-C1, 39 variants; BMI-C2, 82 variants) indicated a positive causal effect on type 2 diabetes risk (consistent with unfavorable adiposity), while one cluster (BMI-C3, 8 variants) suggested a negative causal effect (consistent with favorable adiposity; table S3). Among BMI-C1 and BMI-C2, 1 and 2 variants respectively were previously associated with unfavorable adiposity (15). One variant in BMI-C3 was previously associated with favorable adiposity (table 2) (15). Correlated variants (r^2^ ≥ 0.8) were observed between BFP and BMI clusters, reflecting shared genetic architecture. Importantly, no correlation was noted between unfavorable and favorable adiposity clusters (table S6; figure S3).

We validated the causal effect of adiposity through these clusters on type 2 diabetes (in the unfavorable and favorable direction) using FinnGen (19) as an independent cohort. MR-IVW results against type 2 diabetes risk (odds ratios [95% confidence intervals]) were as follows: BFP-all 2.20 [1.89-2.56], BFP-C1 11.20 [6.90-18.21], BFP-C2 4.42 [3.72-5.25], BFP-C3 1.41 [1.07-1.86], BFP-C4 0.29 [0.18-0.48] and BFP-C5 0.05 [0.030-0.080] per one standard deviation (SD) increase in BFP. For BMI, results were: BMI-all 2.35 [2.19-2.53], BMI-C1 4.23 [3.53-5.07], BMI-C2 2.40 [2.13-2.71] and BMI-C3 0.47 [0.23-0.95] per 1-SD increase in BMI (table S5). The F-statistic (a representation of instrument strength for MR-IVW) was > 50 for all BFP and BMI clusters (table S7).

### The effect of clusters on adiposity-related traits

**Figure 2:**
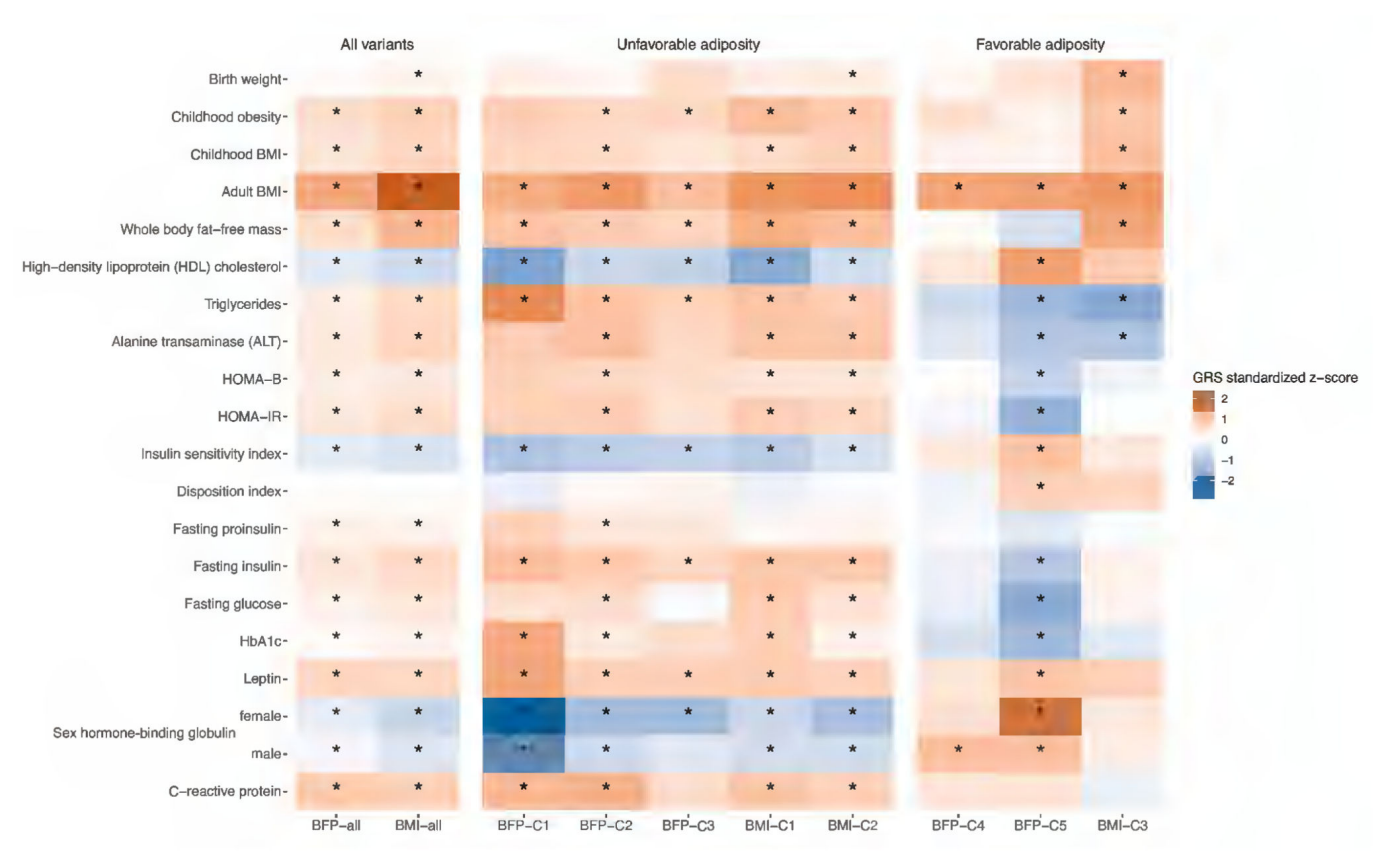
Genetic risk score effects on anthropometric and metabolic biomarkers. For easier comparison, the z-scores displayed are standardized for the number of variants per cluster. P values were corrected using the Benjamini-Hochberg procedure for each cluster. * indicates the result < the adjusted p value threshold 0.05

**Figure 3:**
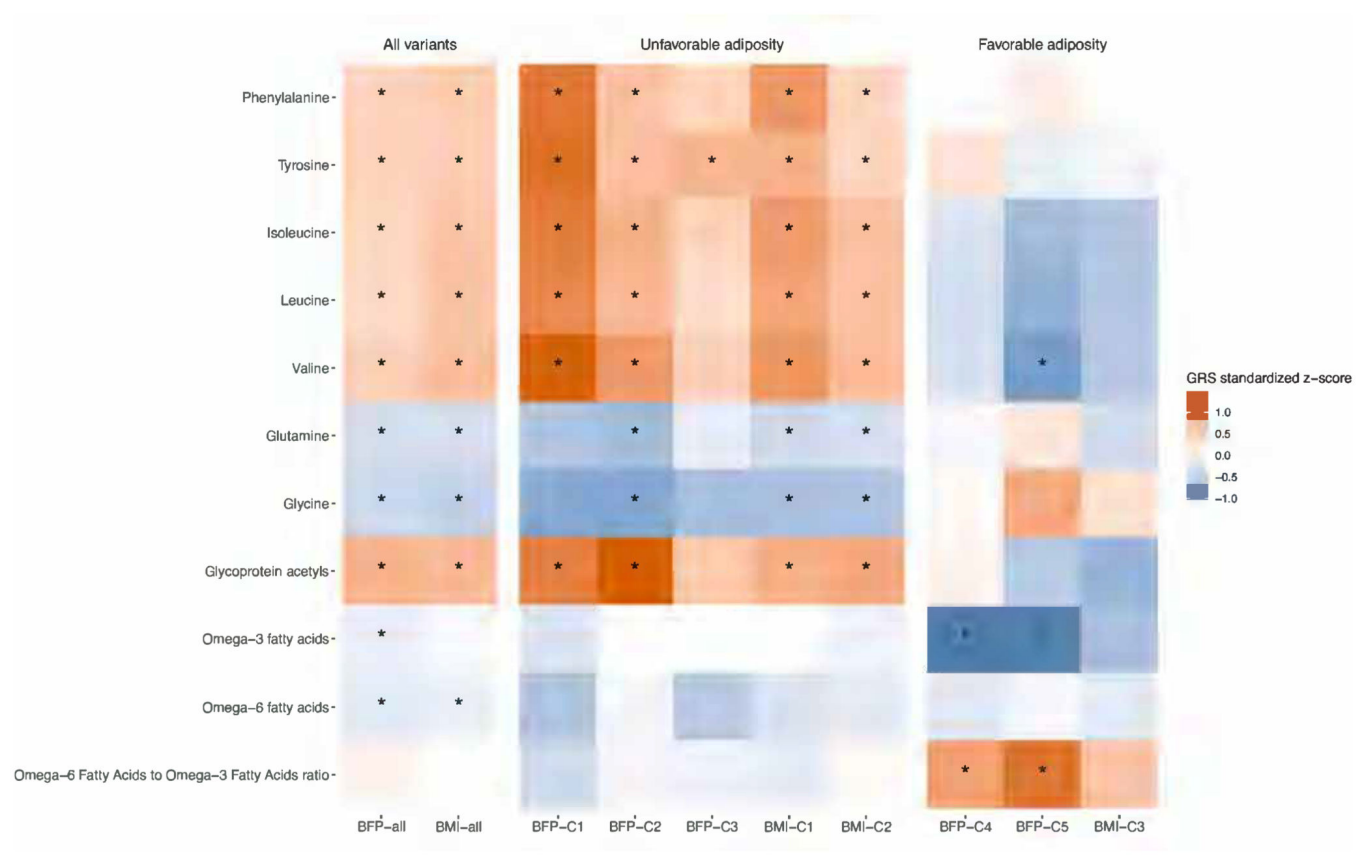
Genetic risk score effects on metabolites. For easier comparison, the z-scores displayed are standardized for the number of variants per cluster. P values were corrected using the Benjamini-Hochberg procedure for each cluster. * indicates the result < the adjusted p value threshold 0.05

**Figure 4:**
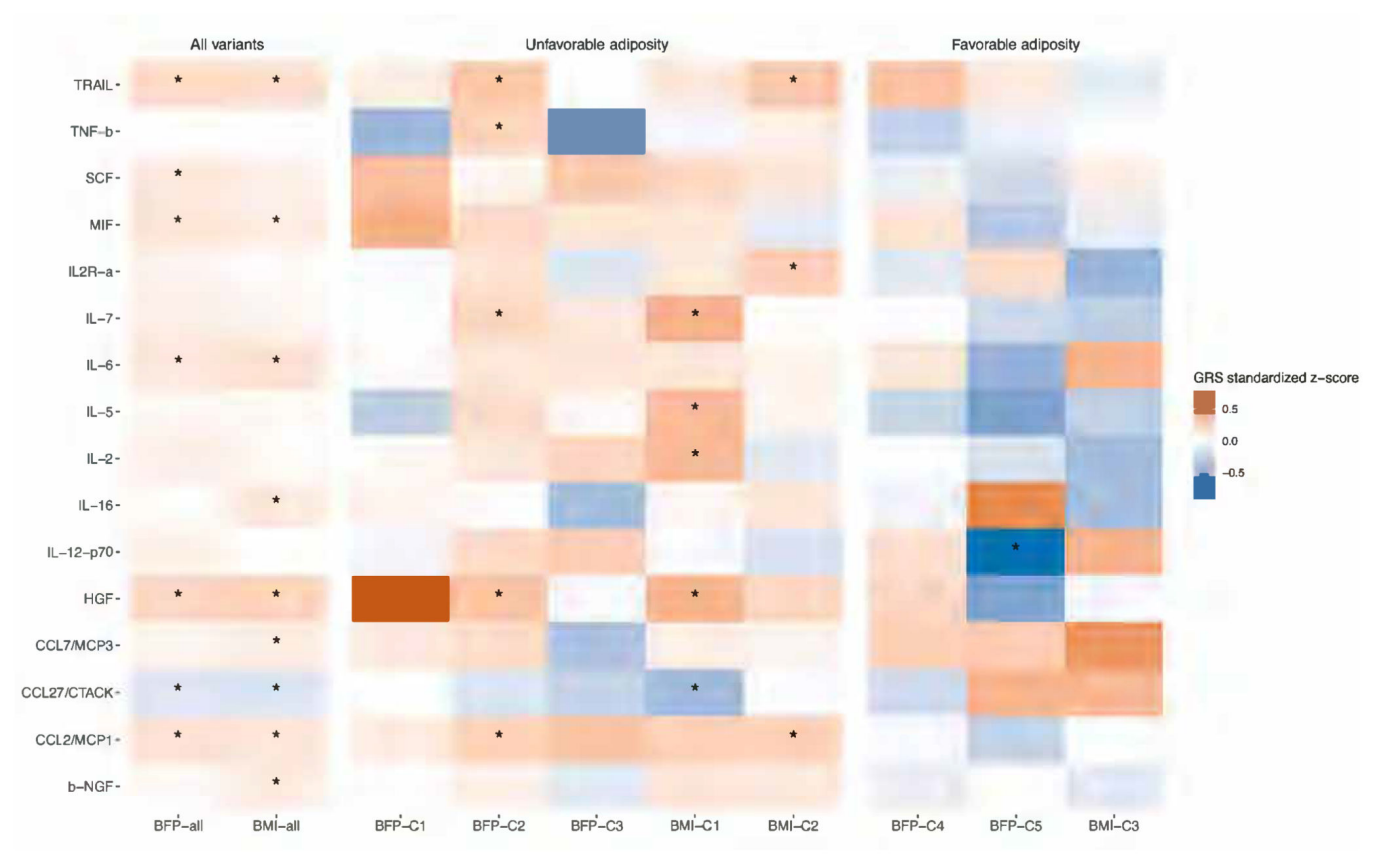
Genetic risk score effects on inflammatory cytokines. For easier comparison, the z-scores displayed are standardized for the number of variants per cluster. P values were corrected using the Benjamini-Hochberg procedure for each cluster. * indicates the result < the adjusted p value threshold 0.05

To investigate differences in cluster metabolic signatures, we generated cluster-specific genetic risk scores and compared the effects of these scores on different adiposity-related traits. We included metabolic biomarkers, anthropometric traits, metabolites, and inflammatory cytokines (figures 2-4, table S4).

The genetic risk scores for all BFP and BMI clusters were associated with higher adult BMI and leptin, regardless of their favorable or adverse metabolic effect. BMI clusters showed more significant associations with higher adiposity from early life (birth weight, childhood obesity, childhood BMI) than BFP clusters. This could be explained by the fact that BMI reflects overall body size, while BFP, focused on the proportion of body weight composed of fat, may be more influenced by factors related to fat distribution and metabolic processes. Comparisons would be more readable if we had a GWAS for childhood body fat percentage. All the unfavorable adiposity clusters (BFP-C1, C2 and C3 and BMI-C1 and C2) were associated with an adverse metabolic profile (higher triglycerides, CRP, liver enzymes, insulin resistance and lower HDL-C and sex-hormone binding globulin) while favorable adiposity clusters (BFP-C4 and C5 and BMI-C3) were associated with a favorable metabolic profile (figure 2).

Genetic risk scores for unfavorable adiposity clusters were associated with insulin resistance-correlated amino acids (37) (with a weaker effect for BFP-C3 but directionally consistent), including phenylalanine, tyrosine, isoleucine, leucine and valine. There was also association with higher glycoprotein acetyls levels, suggesting these clusters affect inflammation (38), and lower glutamine and glycine levels, which are metabolites linked to improved glucose regulation (37) (figure 3; table S5).

Favorable adiposity clusters had a significant association with lower omega-3 levels, and higher omega-6 to omega-3 ratio, whereas unfavorable adiposity clusters had no association with omega-3 or omega-6 (figure 3). Although observational studies link high omega-6 to omega-3 ratios with obesity (39), evidence from randomized controlled trials and Mendelian randomization studies remains inconclusive regarding their causal effects on metabolic outcomes like type 2 diabetes, glucose metabolism, or cardiovascular disease (40,41). Inconsistencies in trial results may stem from factors like study duration, cooking methods, ethnicity, sample size, and fatty acid source.

To further investigate the cluster-specific role of inflammation, as inflammation has been suggested as a mechanism that increases type 2 diabetes risk in people with obesity (42), we used data on pro- and anti-inflammatory cytokines. Genetic risk scores for unfavorable adiposity clusters were associated with higher cytokine levels (TRAIL, TNF-b, IL-7, HGF, CCL2/MCP1 for BFP-C2, and IL-2, IL-5, IL-7, and HGF for BMI-C1). The favorable adiposity cluster BFP-C5 was associated with lower inflammatory cytokine levels (e.g., IL-12; figure 4; table S5).

### The effect of clusters on MRI-derived measures of fat distribution and body composition

**Figure 5:**
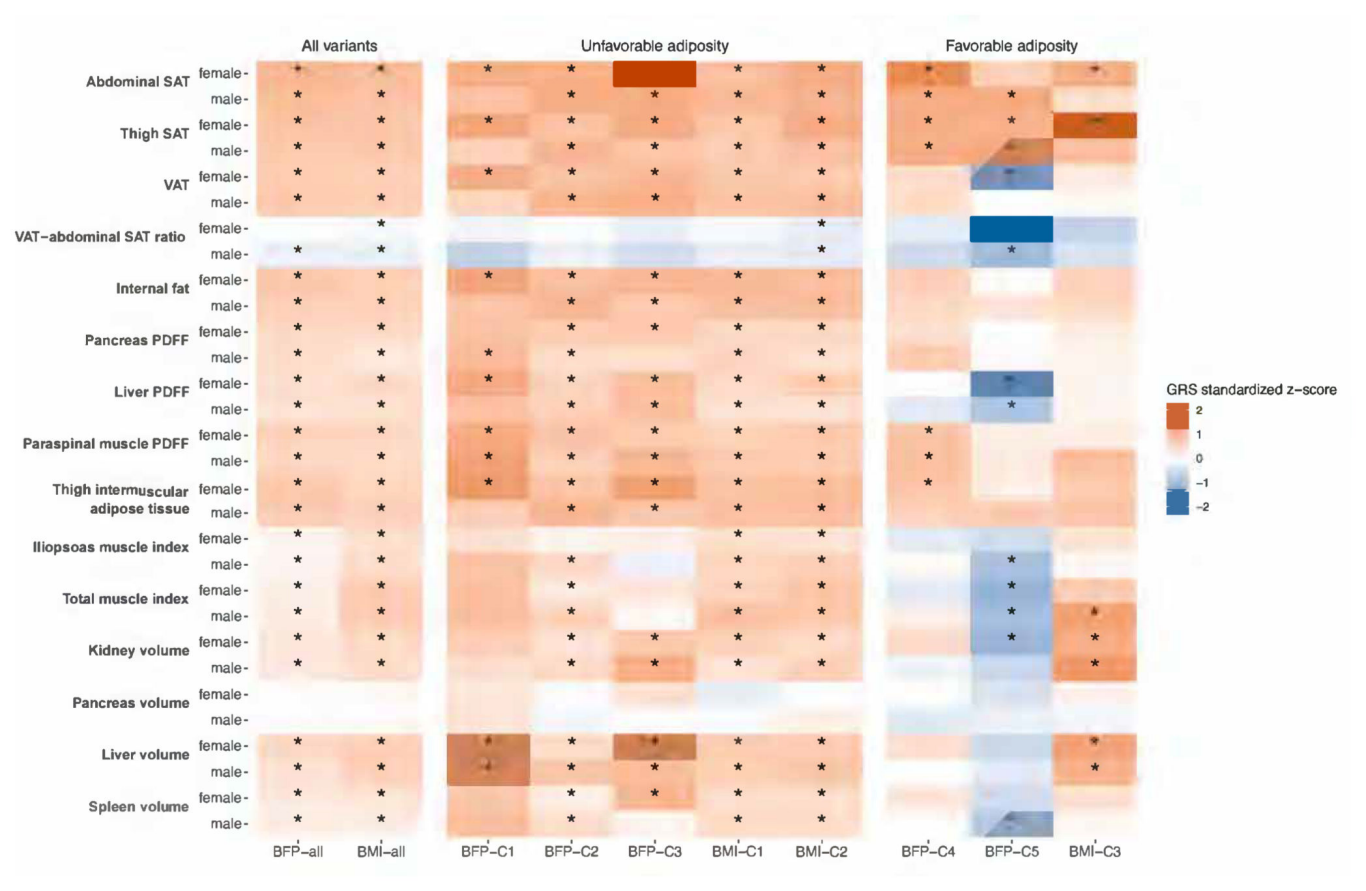
Genetic risk score effects on MRI-derived measures of fat distribution and body composition. For easier comparison, the z-scores displayed are standardized for the number of variants per cluster. P values were corrected using the Benjamini-Hochberg procedure for each cluster. * indicates the result < the adjusted p value threshold 0.05

We used precision MRI-derived measures of fat and body composition to investigate differences in fat distribution patterns of our adiposity clusters. Genetic risk scores for all clusters were associated with higher abdominal and thigh SAT. Unfavorable adiposity clusters were also associated with increased ectopic fat accumulation in pancreas, liver and paraspinal muscle, VAT, internal fat, and thigh intermuscular adipose tissue. They were also associated with higher muscle index and organ volume (kidney, liver, spleen), with some cluster specific effects (figure 5; table S5).

Favorable adiposity clusters had unique and distinct patterns of association with MRI-derived measures. BFP-C4 was associated with higher paraspinal muscle PDFF and higher thigh intermuscular adipose tissue, but no association with liver PDFF, pancreas PDFF, VAT, muscle index measures or organ volume. BFP-C5 was associated with lower liver PDFF, lower VAT-ASAT ratio, lower muscle index measures, and lower kidney and spleen volume. BMI-C3 was associated with higher muscle index and higher kidney and liver volume. These results were consistent in males and females.

### The causal effect of adiposity clusters on risk of type 2 diabetes-related disease outcomes

**Figure 6:**
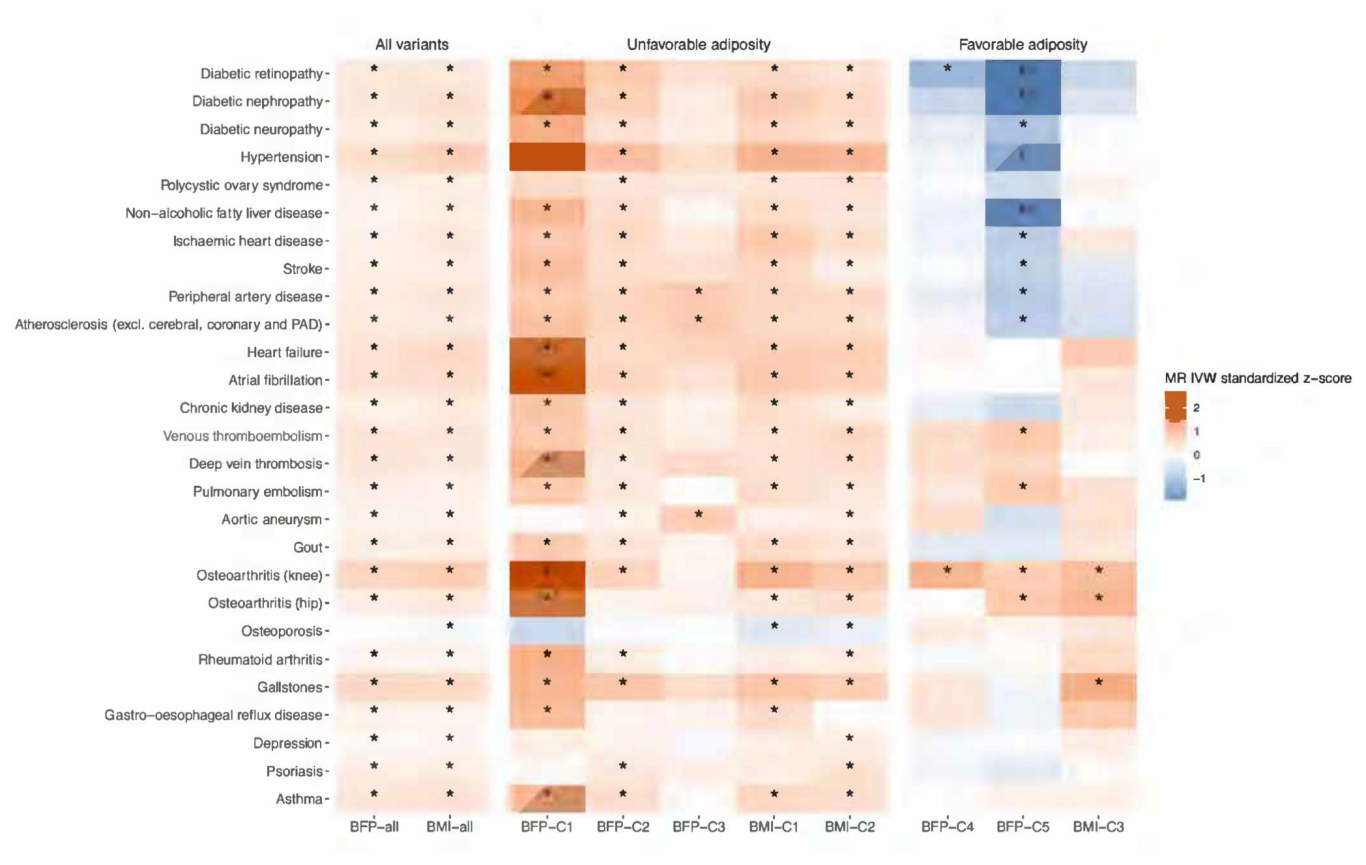
The causal effects of higher adiposity through each cluster on risk of type 2 diabetes and its complications. For easier comparison, the z-scores displayed are standardized for the number of variants per cluster. P values were corrected using the Benjamini-Hochberg procedure for each cluster. * indicates the result < the adjusted p value threshold 0.05

Since the genetically predicted favorable and unfavorable adiposity clusters had distinct effects on different clinical and MRI biomarkers, we used two-sample Mendelian randomization (MR) to investigate differences in causal effect of each cluster on disease risk, including those related to type 2 diabetes (figure 6; table S5). We detected evidence of heterogeneity from MR estimates when we studied the effect of higher adiposity using all BFP and BMI variants (BFP-all and BMI-all, table S5). However, there was no evidence of heterogeneity in the causal estimates when using each cluster. Unfavorable adiposity clusters BFP-C1, BFP-C2, BMI-C1 and BMI-C2 were associated with higher disease risk, including diabetic nephropathy, retinopathy and neuropathy, hypertension, polycystic ovary syndrome, non-alcoholic fatty liver disease, ischemic heart disease, stroke, peripheral artery disease, atherosclerosis, heart failure, atrial fibrillation, chronic kidney disease, thrombotic events, aortic aneurysm, gout, osteoarthritis, gallstones, and asthma. BFP-C3 was only associated with higher risk of peripheral artery disease, atherosclerosis, and aortic aneurysm. We also observed some cluster specific effects among unfavorable adiposity clusters; for example, BFP-C2 and BMI-C2 were associated with higher psoriasis risk.

Among favorable adiposity clusters, BFP-C5 was associated with lower disease risk, including diabetic nephropathy, retinopathy and neuropathy, hypertension, non-alcoholic fatty liver disease, ischemic heart disease, stroke, peripheral artery disease, and atherosclerosis, but it was associated with higher risk of thrombotic events and osteoarthritis. BFP-C4 was associated with lower diabetic retinopathy risk and higher osteoarthritis risk, and BMI-C3 was associated with higher risk of osteoarthritis and gallstones. All results were directionally consistent with those from sensitivity tests (table S5).

### eQTL and pathway enrichment analysis

To explore differences in tissue-specific gene expression for unfavorable and favorable adiposity variant clusters, we counted the number of independent eQTLs in brain and adipose (subcutaneous and visceral) tissue per cluster. When comparing the ratio of independent eQTLs in adipose to brain tissue, unfavorable adiposity clusters BFP-C2 and BMI-C2 were more enriched for eQTLs in the brain, and favorable adiposity clusters were more enriched in adipose tissue (table S8).

All clusters were enriched for different pathways (table S9). Notable pathways for unfavorable adiposity clusters comprised cytoskeletal regulation by Rho GTPase (BFP-C1); JAK/STAT signaling pathway (BFP-C2); Endothelin signaling pathway (BFP-C3); ubiquitin proteasome pathway (BMI-C1); and JAK/STAT signaling pathway (BFP-C2). For favorable adiposity clusters, the Alzheimer disease-amyloid secretase pathway was highlighted (BFP-C4). Of these, only BFP-C3 and BMI-C2 remained significant after correction for multiple testing (FDR<0.05).

## Discussion

We performed clustered MR analyses to identify distinct causal mechanisms linking higher adiposity with type 2 diabetes risk. We identified evidence for multiple causal mechanisms by which adiposity influences type 2 diabetes risk. While most biological mechanisms associated with higher adiposity lead to increased type 2 diabetes risk (e.g. inflammation), there may also be some pathways associated with higher adiposity that lead to lower type 2 diabetes risk. These potentially protective mechanisms relate to lower liver fat and improved insulin sensitivity, or increased body size and enhanced muscle quality.

### Association patterns common to all adiposity clusters

Shared associations across adiposity clusters, irrespective of their favorable or unfavorable metabolic effect, suggest consequences of higher adiposity beyond metabolic impact. For example, association with higher leptin for all clusters was expected, as leptin is produced by adipose tissue. The associations with higher osteoarthritis risk are consistent with previous findings stating that the metabolic effect of adiposity might not be the primary driver of this condition. The higher thrombotic event risk is also in agreement with previous results confirming the causal role of non-metabolic components of higher adiposity, e.g., the mechanical effect of higher weight on blood flow in lower limbs (43).

### The difference between unfavorable and favorable adiposity clusters

Overall, the unfavorable adiposity clusters were associated with an adverse metabolic profile encompassing higher insulin resistance and inflammatory markers, adverse liver profile, and increased ectopic fat deposition (liver, pancreas, paraspinal and thigh muscle). The favorable adiposity clusters were overall associated with a healthy metabolic profile, with an association pattern opposite to the unfavorable adiposity clusters.

The association between unfavorable adiposity clusters and higher organ volume, especially the liver, could be due to increased ectopic fat. No cluster showed an association with pancreas volume, suggesting limited power or a lack of involvement in adiposity-to-diabetes pathways. Although, pancreatic volume tends to decline in diabetes, suggesting volume changes in this organ are more difficult to contextualize. The overall associations with fat distribution were consistent with previous work, where unfavorable adiposity was associated with higher liver, pancreatic and visceral fat, and favorable adiposity was associated with lower liver fat and had no significant effect on pancreatic fat (15,43).

Recent findings show that intentional weight loss in type 2 diabetes reverses many associated amino acid changes (44). Therefore, the opposite effect of favorable and unfavorable adiposity clusters on amino acid levels previously associated with lower insulin sensitivity and higher insulin resistance and type 2 diabetes risk (37) could suggest these amino acids are not causal risk factors, but are biomarkers of metabolically healthy or unhealthy adiposity.

### Differences between unfavorable adiposity clusters

Differences among unfavorable adiposity clusters in associations with biomarkers suggest diverse mechanisms by which higher adiposity leads to adverse metabolic outcomes. BFP-C1 demonstrated a more unfavorable metabolic effect, with the strongest impact on type 2 diabetes risk, circulatory lipids, and surrogates of insulin resistance with no effect on inflammatory cytokines. Cytoskeletal regulation by Rho GTPase was highlighted for BFP-C1 for which there is emerging evidence to implicate a role in metabolic homeostasis by regulating glucose uptake into skeletal muscle and adipose tissue (45). This cluster also had more significant associations with measures of fat distribution and body composition in females.

BFP-C2 and BMI-C2 were associated with cytokines and inflammatory markers, and were both enriched for pathways related to inflammation, suggesting that inflammation is strongly associated with the mechanisms these clusters may represent. Higher adiposity through these clusters was associated with higher risk of psoriasis, possibly through higher inflammation as an underlying mechanism. BFP-C3 was only associated with vascular outcomes including peripheral artery disease, atherosclerosis and aortic aneurysm aligning with the highlighted endothelin signaling pathway for this cluster.

### Differences between favorable adiposity clusters

Similarly, the differences between favorable adiposity clusters associations with metabolic and imaging biomarkers suggest that there is more than one mechanism of adiposity leading to favorable metabolic outcomes. BFP-C5 was more protective against disease risk compared to BFP-C4 and BMI-C3. BFP-C5 was associated with higher insulin sensitivity and lower inflammatory marker levels, whilst BFP-C4 and BMI-C3 were not associated with these measures.

The favorable adiposity clusters also had unique association patterns with measures of fat distribution and body composition. BFP-C5 was associated with lower liver PDFF whilst BFP-C4 and BMI-C3 had no association with liver fat. BFP-C4 was associated with higher subcutaneous fat and paraspinal muscle PDFF but had no association with any other ectopic fat depot.

BMI-C3 could represent an adiposity subtype associated with increased body size regardless of fat, as it was associated with higher measures of early life obesity, muscle index, kidney volume, liver volume, but had no association with any ectopic fat measures. The favorable effect of BMI-C3 could be through increasing skeletal muscle mass, which has been associated with decreased type 2 diabetes risk potentially via increased insulin sensitivity, improved glucose metabolism or acting as a sink for glucose (46,47).

None of the favorable adiposity clusters were associated with pancreatic fat, though this is harder to measure accurately. This is consistent with result of the “twin-cycle” hypothesis, finding that liver fat is more likely to mediate glycemic control in type 2 diabetes than pancreatic fat (15,48).

### Strengths and limitations

We leveraged a range of publicly available GWAS datasets to investigate the complexity between adiposity and type 2 diabetes risk. This research can be expanded as sample sizes and data accessibility improve. We also used gold standard measurements of MRI scans of sex-specific fat and organ content within the UK Biobank to strengthen our analysis and consider sexual dimorphism in body fat distribution.

The GWAS datasets we chose were focused on European populations due to large sample size, potentially limiting the generalizability of our findings to people of other ethnicities and fat distributions (9). Nevertheless, we have shown that previously identified favorable and unfavorable adiposity clusters have a consistent effect across different ethnic groups (49). Second, the biological interpretation of our adiposity cluster variants will require further exploration, as most GWAS variants reside within non-coding regions and often exert their effects alongside correlated variants (50). Third, using genetic associations as a starting point may downplay the influence of environmental factors. This approach necessitates accurate effect estimates, well-established genetic foundations for traits, and large sample sizes, hence why we selected the most current and expansive GWAS studies available. Fourth, in our clustering algorithm, we prioritized the minimization of false-positive findings. While this cautious approach bolsters reliability of our findings, it may leave certain associations unexplored if we overlooked variants that might belong to adiposity clusters. Finally, one key consideration is the strength and distinctiveness of the identified clusters. The interpretation of ‘distinct’ clusters is contingent upon effect size ratios, and we recognize the need for a nuanced evaluation of their robustness. We acknowledge that the observed differences in associations with various traits among clusters may, in some instances, represent differences in magnitude rather than distinct mechanistic pathways.

## Conclusion

Using genetically predicted measures of adiposity and diverse traits, we found evidence for different underlying pathways and subtypes of higher adiposity with contrasting risks for type 2 diabetes and related complications. These novel insights hold potential for advancing precision medicine strategies for type 2 diabetes and related conditions through targeted adiposity management.

## Supplementary Material

Supplementary material

## Figures and Tables

**Table 1:** All publicly available GWAS used. AA − African American, EAS − East Asian, EUR − European.

Trait/disease	PubMed ID	Sample size (case/control for disease if available)	Ethnicity	First author, journal, publication year
Cytokines and growth factors	27989323, 33491305	8293	EUR	Ahola-Olli, A.V. et al, AJHG, 2017; Kalaoja, M et al, Obesity, 2021
Metabolites	35692035	115078	EUR	Borges, C, M. et al, BMC Medicine, 2022. Accessed via IEU OpenGWAS ID: met-d-*
Childhood obesity	31504550	24160	EUR	Bradfield, J.P. et al, Human Molecular Genetics, 2019
Childhood BMI	26604143	35668	EUR	Felix, J, F, et al, Human Molecular Genetics, 2016
HbA1c	34059833	281416	EUR	Chen, J. et al, Nature Genetics, 2021
Adiponectin	22479202	45891 (AA n = 4,232, EAS n = 1,776, EUR n = 29,347)	AA, EAS, EUR	Dastani, Z. et al, PLoS Genetics, 2012
HOMA-B, HOMA-IR	20081858	46186	EUR	Dupuis, J. et al, Nature Genetics, 2010
HDL, LDL and non-HDL cholesterol, Total cholesterol, Triglycerides	34887591, 36575460, 35931049	1320000	EUR	Graham, S.E. et al, Nature, 2021; Kanoni, S. et al, Genome Biology, 2022; Ramdas, S. et al, AJHG, 2022
Leptin	26833098	32161	EUR	Kilpeläinen, T.O. et al, Nature Communications, 2016
Fasting glucose, Fasting insulin	33558525	140595, 98210	EUR	Lagou, V. et al, Nature Communications, 2021
Type 2 Diabetes	35551307	80154/853816	EUR	Mahajan, A. et al, Nature Genetics, 2022
Liver enzymes (ALP, ALT, GGT)	33972514	437438, 437267, 437194	EUR	Pazoki, R. et al, Nature Communications, 2021
Disposition index, corrected insulin response, insulin at 30 mins, incremental insulin at 30 mins	24699409	5318	EUR	Prokopenko, I. et al, PLoS Genetics, 2014
Adult BMI, waist-to-hip ratio (female), waist-to-hip ratio (male)	30239722	806834, 379501, 315284	EUR	Pulit, S.L. et al, Human Molecular Genetics, 2019
Fasting proinsulin	21873549	27079	EUR	Strawbridge, R.J. et al, Diabetes, 2011
Insulin sensitivity index	27416945	16753	EUR	Walford, G.A. et al, Diabetes, 2016
Birth weight	31043758	298142	EUR	Warrington, N.M. et al, Nature Genetics, 2019
Adult height	36224396	4080687	EUR	Yengo, L. et al, Nature, 2022
Body fat percentage	NA	454633	EUR	Elsworth, B. 2018. Accessed via IEU OpenGWAS ID: ukb-b-8909
C-Reactive protein	30388399	204402	EUR	Ligthart, S, AJHG, 2018. Accessed via IEU OpenGWAS ID: ieu-b-35
Whole body fat-free mass	NA	454850	EUR	Elsworth, B. 2018. Accessed via IEU OpenGWAS ID: ukb-b-13354
Sex hormone-binding globulin (female)	NA	214989	EUR	Richmond, R. 2020. Accessed via IEU OpenGWAS ID: ieu-b-4870
Sex hormone-binding globulin (male)	NA	185221	EUR	Richmond, R. 2020. Accessed via IEU OpenGWAS ID: ieu-b-4871
**FinnGen Data Freeze 8 disease outcomes**	36653562	342499	EUR	Kurki, M.I. et al, medRxiv, 2022
Type 2 diabetes		49114/283207		
Diabetic retinopathy		8942/283545		
Diabetic nephropathy		3676/283456		
Diabetic neuropathy		2444/249480		
Hypertension		81138/243756		
Polycystic ovary syndrome		1196/181796		
Non−alcoholic fatty liver disease		1908/340591		
Ischemic heart disease		56730/285769		
Stroke		34560/249480		
Atherosclerosis (excl. cerebral, coronary and PAD)		13434/317899		
Heart failure		23622/317939		
Atrial fibrillation		40594/168000		
Chronic kidney disease		7916/330300		
Venous thromboembolism		17048/325451		
Deep vein thrombosis		8077/295014		
Pulmonary embolism		8170/333487		
Aortic aneurysm		7603/317899		
Gout		7461/221323		
Osteoarthritis (knee)		39343/221323		
Osteoarthritis (hip)		17536/324963		
Osteoporosis		6303/325717		
Rheumatoid arthritis		11178/221323		
Gallstones		32894/301383		
Gastro−esophageal reflux disease		22867/292256		
Depression		38225/299886		
Psoriasis		8075/330975		
Asthma		37253/187112		
Intrahepatic liver and bile duct cancer		648/259583		
Colorectal cancer		5458/259583		
**FinnGen Data Freeze 7 disease outcomes**				
Peripheral artery disease		11924/288638		

**Table 2:** Favorable adiposity variants identified by MR-Clust due to having a decreasing effect on type 2 diabetes risk. Variants not previously identified as favorable adiposity in previous work (15) are considered novel (Y).

Chr:pos (b37)	rsid	Adiposity-increasing allele	Other allele	Cluster	Novel? (Y/N)	Nearest gene
1:203527812	rs2802774	A	C	BFP_C4	N	*OPTC*--[]--*ATP2B4*
2:135597628	rs10496731	T	G	BFP_C4	Y	*ACMSD*
3:123062657	rs9814758	T	G	BFP_C4	Y	*ADCY5*
3:171833266	rs4894808	G	C	BFP_C4	Y	*FNDC3B*
9:136929586	rs55924785	C	T	BFP_C4	Y	*BRD3*
11:27487992	rs11030016	T	C	BFP_C4	Y	*LGR4*
12:121709430	rs75412871	C	T	BFP_C4	Y	*CAMKK2*
12:124409502	rs7133378	A	G	BFP_C4	N	*DNAH10*
15:31689543	rs12441543	A	G	BFP_C4	N	*KLF13*
18:2846812	rs11664106	T	A	BFP_C4	N	*SMCHD1*--[]--*EMILIN2*
19:34008600	rs33836	C	T	BFP_C4	Y	*PEPD*
19:46182304	rs10423928	T	A	BFP_C4	Y	*GIPR*
22:38599767	rs4820323	C	G	BFP_C4	Y	*MAFF/* * PLA2G6*
1:219744138	rs2785988	A	C	BFP_C5	Y	[]--*ZC3H11B*
2:165528876	rs13389219	T	C	BFP_C5	N	*COBLL1*
3:12393125	rs1801282	G	C	BFP_C5	Y	*PPARG*
3:64718258	rs2371767	C	G	BFP_C5	Y	*ADAMTS9*--[]
4:89726283	rs2276936	A	C	BFP_C5	Y	*FAM13A*
6:43757896	rs998584	C	A	BFP_C5	N	*VEGFA*
6:127003464	rs853961	T	G	BFP_C5	Y	*CENPW*--[]--*RSPO3*
7:130466854	rs972283	A	G	BFP_C5	N	*KLF14*--[]-- *MKLN1*
7:150542711	rs6977416	G	A	BFP_C5	N	*AOC1*
1:11284336	rs10779751	A	G	BMI_C3	Y	*MTOR*
3:48085349	rs11919665	A	T	BMI_C3	Y	*MAP4*
6:130384187	rs9375702	C	T	BMI_C3	Y	*L3MBTL3*
7:93085722	rs2283006	A	G	BMI_C3	Y	*CALCR*
12:122963550	rs12369179	C	T	BMI_C3	N	*ZCCHC8*
14:91512339	rs1951455	C	T	BMI_C3	Y	*RPS6KA5*
19:46180184	rs11672660	C	T	BMI_C3	Y	*GIPR*
20:62691550	rs6512302	C	G	BMI_C3	Y	*TCEA2*

## Data Availability

All data supporting the findings of this study are available within the paper and its Supplementary Information. Publicly available GWAS summary statistics are available online.

## Article Highlights

- The relationship between excess adiposity and type 2 diabetes is complex.
- Can genetic subtypes of adiposity reveal distinct pathways linking adiposity with type 2 diabetes?
- Higher adiposity increases type 2 diabetes risk via different mechanisms (e.g. cholesterol pathways or inflammation) but decreases risk via other mechanisms (lower liver fat and improved insulin sensitivity, or increased body size and enhanced muscle quality).
- These insights could improve precision medicine for type 2 diabetes via treating adiposity.
