# Supplementary material for: Genetic evidence for distinct biological mechanisms that link adiposity to type 2 diabetes: towards precision medicine
Source: Diabetes. Author manuscript; Available in PMC 2024 Jun 17. (PMC11109787; doi:10.2337/db23-1005)

Navigate to:

Table S1: Image-derived phenotype (IDP) GWAS data sources.
Table S2: Output from MR-Clust for body fat percentage (BFP).
Table S3: Output from MR-Clust for body mass index (BMI).
Table S4: The association between cluster genetic-risk scores and biomarkers.
Table S5: Mendelian Randomization results for disease outcomes.
Table S6: Correlated variants between body fat percentage and BMI clusters.
Table S7: F-statistics for each BFP/BMI-increasing cluster.
Table S8: eQTL analysis results.
Table S9: Pathway analysis results for all adiposity-increasing clusters.

Figure S1: Study design.
Figure S2: Scatter plots of genetic association between adiposity and type 2 diabetes.
Figure S3: Venn diagram showing number of variants either correlated with or shared between adiposity-increasing clusters.

Table S1: Image-derived phenotype (IDP) GWAS data sources. ASAT – abdominal subcutaneous adipose tissue, VAT – visceral adipose tissue, PDFF – proton density fat fraction, SAT – subcutaneous adipose tissue.

IDP	PubMed ID/link to preprint	Sample size	Sample size - male	Sample size - female
ASAT volume	34128465	37589	18320	19269
VAT volume	34128465	37589	18320	19269
VAT/ASAT ratio		37589	18320	19269
Internal fat volume	https://www.medrxiv.org/content/10.1101/2023.03.02.23286689v3	37589	18320	19269
Pancreas PDFF	34128465	28587	14040	14547
Liver PDFF	34128465	30161	14753	15408
Paraspinal muscle PDFF	35260612	30175	14760	15415
Thigh internal fat/muscle ratio	https://www.medrxiv.org/content/10.1101/2023.03.02.23286689v3	37584	18318	19266
Thigh SAT	https://www.medrxiv.org/content/10.1101/2023.03.02.23286689v3	37584	18318	19266
Iliopsoas muscle index	https://www.medrxiv.org/content/10.1101/2023.03.02.23286689v3	36426	17788	18638
Total muscle index	https://www.medrxiv.org/content/10.1101/2023.03.02.23286689v3	36426	17788	18638
Kidney volume	34128465	37589	18320	19269
Pancreas volume	34128465	36133	17627	18506
Liver volume	34128465	37589	18320	19269
Spleen volume	34128465	37589	18320	19269

Table S2: Output from MR-Clust for body fat percentage (BFP). Each BFP variant was assigned to a cluster with a probability of inclusion within that cluster. The highest cluster inclusion probability for each BFP variant as shown here was used to assign a variant to a cluster. Each cluster had a minimum inclusion probability of 0.8 and contained a minimum of 4 variants.

observation (b37)	cluster	probability	cluster_mean	Theta	theta_se	cluster_class	cluster_size
chr1:32197257	2	0.85	1.711	1.648316	0.606523	2	101
chr1:47690438	2	0.884	1.711	1.644387	0.553809	2	101
chr1:49879122	3	0.826	0.613	0.525808	0.5039	3	14
chr1:62570321	2	0.955	1.711	1.694538	0.459536	2	101
chr1:96924097	2	0.894	1.711	1.471771	0.442894	2	101
chr1:149906413	3	0.906	0.613	0.452859	0.365561	3	14
chr1:177889480	2	0.999	1.711	1.813402	0.268529	2	101
chr1:201800511	2	0.942	1.711	2.076439	0.491595	2	101
chr1:203527812	4	0.837	-1.542	-2.09525	0.63235	4	13
chr1:219744138	5	0.972	-3.241	-2.77609	0.332182	5	9
chr1:243533273	2	0.837	1.711	1.673858	0.637987	2	101
chr2:422144	2	0.975	1.711	2.126749	0.336022	2	101
chr2:628504	2	0.999	1.711	1.720033	0.302072	2	101
chr2:25150116	3	0.938	0.613	0.450839	0.28568	3	14
chr2:46899742	2	0.869	1.711	1.59011	0.549913	2	101
chr2:59335104	2	0.838	1.711	2.338103	0.422157	2	101
chr2:69582028	3	0.834	0.613	0.489585	0.497235	3	14
chr2:76185292	2	0.838	1.711	2.022576	0.725489	2	101
chr2:100838157	2	0.939	1.711	1.59812	0.44969	2	101

				9			
chr2:105404221	2	0.805	1.711	1.433385	0.536472	2	101
chr2:135597628	4	0.931	-1.542	-1.69975	0.615726	4	13
chr2:138416391	2	0.824	1.711	2.241846	0.747282	2	101
chr2:147903382	2	0.901	1.711	2.112777	0.584385	2	101
chr2:165528876	5	0.986	-3.241	-3.5401	0.380342	5	9
chr2:166185707	2	0.857	1.711	1.724709	0.627167	2	101
chr2:181329491	2	0.909	1.711	2.133848	0.55322	2	101
chr2:205375909	2	0.911	1.711	1.795562	0.563778	2	101
chr2:212286934	2	0.846	1.711	1.829428	0.677566	2	101
chr2:228980358	2	0.842	1.711	2.345557	0.541905	2	101
chr2:230624929	2	0.878	1.711	1.450766	0.448419	2	101
chr3:12393125	5	0.991	-3.241	-3.36376	0.322098	5	9
chr3:49920571	2	0.992	1.711	1.805786	0.38142	2	101
chr3:64718258	5	0.878	-3.241	-4.50008	0.601705	5	9
chr3:71681487	2	0.903	1.711	1.953629	0.59899	2	101
chr3:94038085	2	0.965	1.711	1.898192	0.472702	2	101
chr3:123062657	4	0.869	-1.542	-2.17577	0.492488	4	13
chr3:131629716	2	0.877	1.711	2.274789	0.511108	2	101
chr3:171833266	4	0.885	-1.542	-1.63686	0.664663	4	13
chr3:173656059	2	0.833	1.711	2.165779	0.737286	2	101
chr3:183467986	2	0.824	1.711	2.378794	0.643444	2	101
chr3:185805294	2	0.864	1.711	1.842782	0.651209	2	101
chr4:45182527	2	0.99	1.711	2.05259	0.32226	2	101

				4	7		
chr4:89726283	5	0.896	-3.241	-2.8416	0.518126	5	9
chr4:100239319	2	0.856	1.711	2.218933	0.661165	2	101
chr4:103188709	2	0.885	1.711	1.420871	0.416995	2	101
chr4:130724740	2	0.851	1.711	2.178257	0.689448	2	101
chr4:140863365	2	0.924	1.711	2.122999	0.517622	2	101
chr5:74472939	2	0.927	1.711	2.100957	0.52129	2	101
chr5:75003678	1	0.959	2.891	2.92778	0.379095	1	7
chr5:77389973	2	0.806	1.711	1.774692	0.731329	2	101
chr5:87682877	2	0.867	1.711	2.254712	0.600737	2	101
chr5:87986314	2	0.982	1.711	1.643261	0.379214	2	101
chr5:104022239	2	0.881	1.711	1.895173	0.631724	2	101
chr5:119389031	2	0.865	1.711	2.163876	0.657252	2	101
chr5:153551892	2	0.858	1.711	1.809803	0.653022	2	101
chr5:170506141	2	0.915	1.711	1.707136	0.531608	2	101
chr6:40367138	2	0.923	1.711	2.143497	0.506511	2	101
chr6:43757896	5	0.965	-3.241	-3.86605	0.692269	5	9
chr6:50816887	1	0.891	2.891	2.782244	0.37488	1	7
chr6:97764299	2	0.828	1.711	1.864808	0.718058	2	101
chr6:98550289	2	0.932	1.711	1.478165	0.395827	2	101
chr6:127003464	5	0.973	-3.241	-3.85094	0.524384	5	9
chr6:130165691	2	0.83	1.711	2.115935	0.745177	2	101
chr7:2103739	2	0.887	1.711	1.890656	0.620706	2	101
chr7:32378979	2	0.928	1.711	2.01719	0.54563	2	101

				6	5		
chr7:50697051	2	0.843	1.711	2.218341	0.698542	2	101
chr7:75081418	3	0.816	0.613	0.594435	0.502984	3	14
chr7:104898337	2	0.852	1.711	2.148829	0.691818	2	101
chr7:130466854	5	0.986	-3.241	-3.19982	0.454123	5	9
chr7:150542711	5	0.961	-3.241	-3.20754	0.575496	5	9
chr8:9777555	2	0.821	1.711	2.372128	0.681987	2	101
chr8:27261138	2	0.845	1.711	1.699638	0.635919	2	101
chr8:30865733	2	0.914	1.711	2.070742	0.566357	2	101
chr8:77226919	3	0.896	0.613	0.591537	0.420649	3	14
chr8:143611456	2	0.832	1.711	2.242874	0.72317	2	101
chr8:144505785	2	0.804	1.711	1.757591	0.727279	2	101
chr9:11469190	2	0.803	1.711	1.744846	0.724276	2	101
chr9:15910044	3	0.944	0.613	0.509888	0.31993	3	14
chr9:16739763	2	0.807	1.711	1.375967	0.477667	2	101
chr9:129408290	2	0.897	1.711	2.133356	0.587321	2	101
chr9:136929586	4	0.855	-1.542	-1.90353	0.69661	4	13
chr10:16750129	2	0.841	1.711	2.282559	0.67779	2	101
chr10:102447647	2	0.929	1.711	1.888537	0.546682	2	101
chr11:27487992	4	0.965	-1.542	-1.5766	0.543134	4	13
chr11:27695464	2	0.992	1.711	1.761896	0.372917	2	101
chr11:43692423	2	0.978	1.711	2.001362	0.410837	2	101
chr11:47529947	2	0.998	1.711	1.626506	0.292052	2	101
chr11:66060546	2	0.879	1.711	1.49197	0.47660	2	101

					2		
chr11:89922417	2	0.877	1.711	1.828355	0.626381	2	101
chr11:118912982	2	0.877	1.711	2.072677	0.64708	2	101
chr11:130745719	3	0.801	0.613	0.355471	0.541671	3	14
chr12:2159556	2	0.847	1.711	1.809337	0.670878	2	101
chr12:50263148	2	0.996	1.711	1.707877	0.33448	2	101
chr12:108618630	1	0.811	2.891	2.739697	0.391385	1	7
chr12:121709430	4	0.882	-1.542	-1.69622	0.674789	4	13
chr12:123076778	3	0.854	0.613	0.375852	0.435512	3	14
chr12:124409502	4	0.993	-1.542	-1.70095	0.366026	4	13
chr13:27998600	2	0.83	1.711	2.356289	0.646825	2	101
chr13:54107352	2	0.912	1.711	2.19703	0.491101	2	101
chr13:54372377	2	0.913	1.711	1.918635	0.579211	2	101
chr13:59285126	2	0.848	1.711	1.82232	0.672234	2	101
chr13:79580919	2	0.922	1.711	2.059037	0.550188	2	101
chr13:91993627	2	0.848	1.711	1.999669	0.704919	2	101
chr13:99113166	2	0.937	1.711	1.72229	0.50025	2	101
chr14:29681138	2	0.942	1.711	1.635555	0.460987	2	101
chr14:51207741	2	0.841	1.711	2.262693	0.686991	2	101
chr14:69753369	2	0.931	1.711	1.944849	0.546213	2	101
chr14:79945162	1	0.925	2.891	2.941648	0.411083	1	7
chr15:31689543	4	0.844	-1.542	-1.36779	0.622518	4	13
chr15:41456374	2	0.844	1.711	1.812241	0.676069	2	101
chr15:68127769	2	0.841	1.711	1.329611	0.383269	2	101

chr15:73431600	2	0.884	1.711	2.268049	0.47906	2	101
chr15:84521398	3	0.913	0.613	0.915812	0.309695	3	14
chr15:89415247	3	0.846	0.613	0.445157	0.477889	3	14
chr15:99240793	2	0.802	1.711	1.5551	0.631513	2	101
chr16:4015729	3	0.81	0.613	0.25817	0.455194	3	14
chr16:24726237	2	0.874	1.711	1.755692	0.610675	2	101
chr16:28883241	2	0.811	1.711	1.236728	0.280095	2	101
chr16:30120308	2	0.838	1.711	2.006067	0.724705	2	101
chr16:53806453	1	0.998	2.891	2.961031	0.158409	1	7
chr16:69549749	2	0.944	1.711	2.160127	0.415651	2	101
chr16:89818340	2	0.849	1.711	2.299428	0.627995	2	101
chr17:1835482	2	0.926	1.711	1.525861	0.431847	2	101
chr17:7101292	2	0.845	1.711	2.009995	0.710053	2	101
chr17:21281663	2	0.83	1.711	2.365649	0.614335	2	101
chr17:47090785	1	0.965	2.891	3.688198	0.529076	1	7
chr17:65832016	2	0.884	1.711	2.283527	0.379009	2	101
chr17:78757626	2	0.928	1.711	1.845673	0.544237	2	101
chr18:2846812	4	0.917	-1.542	-1.73731	0.636711	4	13
chr18:22164216	2	0.822	1.711	1.787633	0.706739	2	101
chr18:39910592	2	0.874	1.711	1.879332	0.64195	2	101
chr18:40768309	2	0.949	1.711	1.748544	0.48611	2	101
chr18:57829135	2	0.982	1.711	2.140789	0.294604	2	101
chr19:4050424	3	0.809	0.613	0.572624	0.514193	3	14

chr19:18383506	3	0.896	0.613	0.796201	0.378337	3	14
chr19:18812785	2	0.947	1.711	2.082332	0.473574	2	101
chr19:34008600	4	0.991	-1.542	-1.77041	0.396816	4	13
chr19:46182304	4	0.949	-1.542	-1.04515	0.344083	4	13
chr19:47602577	1	0.826	2.891	3.519555	0.639919	1	7
chr20:41990761	2	0.838	1.711	1.73826	0.662838	2	101
chr20:51100420	2	0.933	1.711	2.041706	0.527632	2	101
chr21:46581798	2	0.834	1.711	1.460832	0.516537	2	101
chr22:38599767	4	0.993	-1.542	-1.48185	0.383746	4	13
chr22:48874412	2	0.827	1.711	1.608564	0.622361	2	101

Table S3: Output from MR-Clust for body mass index (BMI). Each BMI variant was assigned to a cluster with a probability of inclusion within that cluster. The highest cluster inclusion probability for each body fat percentage variant as shown here was used to assign a variant to a cluster. Each cluster had a minimum inclusion probability of 0.8 and contained a minimum of 4 variants.

**observation (b37)**	**cluster**	**probability**	**cluster_mean**	**Theta**	**theta_se**	**cluster_class**	**cluster_size**
chr1:1590521	2	0.851	1.005	0.77	0.475	2	82
chr1:11284336	3	0.884	-0.765	-1.69466	0.549618	3	8
chr1:62579891	1	0.927	1.633	1.827196	0.320113	1	39
chr1:66461401	2	0.826	1.005	0.853846	0.492308	2	82
chr1:72751185	2	0.976	1.005	0.976378	0.259843	2	82
chr1:98315893	2	0.803	1.005	0.455621	0.431953	2	82
chr1:110154688	2	0.986	1.005	0.75	0.281056	2	82
chr1:112289983	1	0.913	1.633	2.319149	0.460993	1	39
chr1:156049877	2	0.951	1.005	0.844291	0.3391	2	82
chr1:174321997	2	0.829	1.005	0.529148	0.466368	2	82
chr1:177889480	2	0.988	1.005	1.141962	0.169102	2	82
chr1:190239907	2	0.832	1.005	0.979866	0.436242	2	82
chr1:194965200	2	0.824	1.005	0.730159	0.515873	2	82
chr1:243684019	2	0.832	1.005	0.878173	0.477157	2	82
chr2:430975	2	0.996	1.005	0.996805	0.200213	2	82
chr2:632348	2	1	1.005	0.852941	0.147059	2	82
chr2:26949366	2	0.875	1.005	0.592105	0.421053	2	82
chr2:50233352	2	0.85	1.005	0.980769	0.413462	2	82
chr2:59307725	1	0.925	1.633	1.871134	0.335052	1	39

chr2:67837553	2	0.902	1.005	0.866667	0.393939	2	82
chr2:161265910	1	0.913	1.633	2.825	0.55	1	39
chr2:181575281	1	0.86	1.633	1.963636	0.412121	1	39
chr2:229002620	1	0.809	1.633	1.972973	0.452703	1	39
chr3:9507314	1	0.81	1.633	2.0553	0.479263	1	39
chr3:42306294	2	0.872	1.005	0.705357	0.450893	2	82
chr3:42418446	2	0.846	1.005	0.761628	0.482558	2	82
chr3:48085349	3	0.859	-0.765	-1.71795	0.589744	3	8
chr3:49924424	2	0.841	1.005	1.199187	0.260163	2	82
chr3:61236462	2	0.889	1.005	0.577381	0.386905	2	82
chr3:84221774	2	0.825	1.005	0.824176	0.5	2	82
chr3:104606144	2	0.844	1.005	0.931973	0.442177	2	82
chr3:135932359	1	0.844	1.633	1.920213	0.409575	1	39
chr3:141275436	2	0.934	1.005	0.795455	0.369318	2	82
chr3:154034950	2	0.865	1.005	0.89404	0.430464	2	82
chr3:185834499	2	0.99	1.005	0.857143	0.263492	2	82
chr4:3298800	1	0.849	1.633	2.48503	0.562874	1	39
chr4:25408838	2	0.951	1.005	1.037543	0.269625	2	82
chr4:28489339	2	0.864	1.005	0.875	0.4375	2	82
chr4:52752812	1	0.873	1.633	2.843478	0.608696	1	39
chr4:96030402	1	0.825	1.633	2.477876	0.584071	1	39
chr4:103188709	2	0.956	1.005	0.982906	0.288462	2	82
chr4:112691776	2	0.821	1.005	0.710744	0.520661	2	82
chr4:137083193	1	0.927	1.633	2.084337	0.391566	1	39

chr4:162129844	2	0.812	1.005	0.798387	0.524194	2	82
chr5:63026280	2	0.815	1.005	1.058065	0.412903	2	82
chr5:63977815	2	0.848	1.005	0.681482	0.481482	2	82
chr5:75015242	1	0.968	1.633	1.692029	0.242754	1	39
chr5:95864693	2	0.805	1.005	0.931298	0.496183	2	82
chr5:105876806	1	0.836	1.633	2.456897	0.568966	1	39
chr5:107439012	2	0.935	1.005	0.623016	0.337302	2	82
chr5:152529936	2	0.804	1.005	0.727273	0.545455	2	82
chr6:13180454	2	0.805	1.005	0.533835	0.518797	2	82
chr6:31643399	1	0.959	1.633	2.502793	0.430168	1	39
chr6:50798526	1	0.811	1.633	1.448529	0.20098	1	39
chr6:97753952	2	0.812	1.005	0.993289	0.456376	2	82
chr6:126042783	1	0.827	1.633	2.73	0.64	1	39
chr6:130384187	3	0.826	-0.765	-1.87736	0.650943	3	8
chr6:153392002	1	0.915	1.633	2.353333	0.466667	1	39
chr6:163033350	2	0.839	1.005	1	0.417021	2	82
chr7:1872921	1	0.81	1.633	1.982036	0.45509	1	39
chr7:50614173	2	0.838	1.005	0.815385	0.484615	2	82
chr7:93085722	3	0.9	-0.765	-1.72727	0.484849	3	8
chr7:113028634	2	0.929	1.005	0.676136	0.369318	2	82
chr7:113362799	2	0.888	1.005	0.786667	0.426667	2	82
chr8:10787612	1	0.975	1.633	2.48	0.371429	1	39
chr8:73435964	2	0.951	1.005	0.831683	0.341584	2	82
chr8:76806584	2	0.929	1.005	0.59715	0.32701	2	82

				6	4		
chr8:116670347	2	0.869	1.005	1.005495	0.379121	2	82
chr8:118884379	2	0.825	1.005	0.964126	0.452915	2	82
chr9:15634326	2	0.957	1.005	0.772487	0.338624	2	82
chr9:16720329	2	0.821	1.005	0.989247	0.446237	2	82
chr9:28410996	1	0.833	1.633	1.603376	0.28692	1	39
chr9:34124860	1	0.887	1.633	2.756757	0.576577	1	39
chr9:81371441	1	0.896	1.633	3.037383	0.598131	1	39
chr9:103088321	2	0.889	1.005	0.820988	0.419753	2	82
chr9:120378483	2	0.825	1.005	0.902985	0.477612	2	82
chr9:131015279	2	0.812	1.005	0.869231	0.507692	2	82
chr10:88110925	1	0.881	1.633	2.287879	0.484849	1	39
chr10:99778226	2	0.905	1.005	0.624242	0.387879	2	82
chr11:13294268	2	0.878	1.005	0.783784	0.439189	2	82
chr11:30422068	2	0.805	1.005	0.978417	0.47482	2	82
chr11:46174948	2	0.815	1.005	0.758687	0.525097	2	82
chr11:69445173	2	0.845	1.005	0.569343	0.459854	2	82
chr11:76476030	1	0.836	1.633	2.229299	0.509554	1	39
chr11:93221105	1	0.885	1.633	3.087379	0.621359	1	39
chr11:118944675	1	0.888	1.633	2.228571	0.464286	1	39
chr11:134601012	2	0.864	1.005	0.743316	0.459893	2	82
chr12:939480	2	0.948	1.005	0.695067	0.345292	2	82
chr12:24060075	2	0.821	1.005	0.841026	0.502564	2	82
chr12:49399132	2	0.814	1.005	0.79178	0.52199	2	82

				9	4		
chr12:50247468	2	0.936	1.005	1.151515	0.222222	2	82
chr12:56494991	2	0.846	1.005	0.87234	0.460993	2	82
chr12:89757937	2	0.862	1.005	0.830986	0.450704	2	82
chr12:122963550	3	0.836	-0.765	-0.74118	0.338235	3	8
chr13:33184288	2	0.858	1.005	0.922078	0.428571	2	82
chr13:58484786	1	0.923	1.633	2.174129	0.41791	1	39
chr13:86494817	2	0.826	1.005	1.029412	0.417647	2	82
chr14:62360075	2	0.886	1.005	0.627907	0.418605	2	82
chr14:79940383	1	0.991	1.633	2.098859	0.292776	1	39
chr14:91512339	3	0.833	-0.765	-1.10811	0.47973	3	8
chr14:94031914	2	0.962	1.005	0.777228	0.331683	2	82
chr15:53072673	1	0.876	1.633	3.097222	0.638889	1	39
chr15:68086838	2	0.91	1.005	1.130873	0.258389	2	82
chr15:78024806	2	0.865	1.005	0.614458	0.445783	2	82
chr16:3599655	1	0.808	1.633	2.10559	0.496894	1	39
chr16:19941968	2	0.809	1.005	1.139241	0.35443	2	82
chr16:28883241	2	0.986	1.005	1.007018	0.22807	2	82
chr16:49011249	2	0.803	1.005	0.643836	0.547945	2	82
chr16:53712135	2	0.834	1.005	1.022624	0.411765	2	82
chr16:53797908	1	1	1.633	1.618812	0.107261	1	39
chr16:69556715	1	0.977	1.633	2.102564	0.333333	1	39
chr17:2138828	2	0.808	1.005	0.785714	0.531746	2	82
chr17:65870073	1	0.903	1.633	3	0.48192	1	39

					8		
chr17:78611724	2	0.913	1.005	0.944444	0.355556	2	82
chr18:1839601	1	0.897	1.633	2.110048	0.425837	1	39
chr18:40708038	1	0.802	1.633	2.043796	0.481752	1	39
chr18:56882326	1	0.814	1.633	2.111842	0.493421	1	39
chr18:57829135	2	1	1.005	0.987319	0.13587	2	82
chr18:58049656	2	0.968	1.005	0.785714	0.32	2	82
chr19:1865901	2	0.863	1.005	0.854305	0.443709	2	82
chr19:2244849	2	0.822	1.005	0.820789	0.505376	2	82
chr19:4060707	2	0.846	1.005	0.545918	0.443878	2	82
chr19:18454825	2	0.868	1.005	0.860465	0.436047	2	82
chr19:30272202	2	0.889	1.005	0.549223	0.352332	2	82
chr19:46180184	3	0.975	-0.765	-0.72189	0.236686	3	8
chr19:47602577	1	0.909	1.633	1.817391	0.330435	1	39
chr20:47689036	2	0.867	1.005	0.80198	0.450495	2	82
chr20:62691550	3	0.822	-0.765	-2.04478	0.567164	3	8
chr22:41804716	1	0.815	1.633	2.039735	0.470199	1	39

Table S4: The association between adiposity-increasing cluster genetic-risk scores and biomarkers. There are 10 clusters: BFP-all (all independent body fat percentage variants), BFP-C1, BFP-C2, BFP-C3, BFP-C4, BFP-C5, BMI-all (all independent body mass index variants), BMI-C1, BMI-C2, BMI-C3. nsnp – number of variants, BHp – Benjamini-Hochberg corrected p-value to account for false discovery rate.

Outcome	Exposure	Nsnp	Beta	SE	pval	BHp
Acetone	BFP-all	388	0.000185	0.000307	0.547874	0.611792
Acetone	BFP-C1	7	0.000329	0.001753	0.851044	0.998836
Acetone	BFP-C2	101	-0.00074	0.000504	0.140118	0.20862
Acetone	BFP-C3	14	-0.00082	0.001449	0.57077	0.742555
Acetone	BFP-C4	13	0.004794	0.002542	0.059346	0.266607
Acetone	BFP-C5	9	0.005016	0.002614	0.054992	0.118854
Acetone	BMI-all	540	-5.2E-05	0.000236	0.825441	0.871334
Acetone	BMI-C1	39	-0.00113	0.001002	0.258156	0.356628
Acetone	BMI-C2	82	-2.8E-05	0.000654	0.966124	0.987218
Acetone	BMI-C3	8	0.008082	0.004463	0.070166	0.270016
Acetoacetate	BFP-all	388	0.002168	0.000282	1.37E-14	5.09E-14
Acetoacetate	BFP-C1	7	7.46E-05	0.001752	0.966019	0.999247
Acetoacetate	BFP-C2	101	0.001983	0.000476	3.12E-05	7.1E-05
Acetoacetate	BFP-C3	14	0.001978	0.001543	0.1999	0.382665
Acetoacetate	BFP-C4	13	0.004533	0.002212	0.040421	0.225685
Acetoacetate	BFP-C5	9	0.003759	0.001554	0.015571	0.052162
Acetoacetate	BMI-all	540	0.001819	0.000222	2.34E-16	5.8E-16
Acetoacetate	BMI-C1	39	0.001059	0.00075	0.158116	0.2303
Acetoacetate	BMI-C2	82	0.001909	0.000624	0.0022	0.004997
Acetoacetate	BMI-C3	8	0.00651	0.003459	0.059844	0.243005

Acetate	BFP-all	388	-0.00193	0.000285	1.22E-11	3.71E-11
Acetate	BFP-C1	7	-0.00342	0.001765	0.052887	0.141738
Acetate	BFP-C2	101	-0.0031	0.000488	2.19E-10	6.99E-10
Acetate	BFP-C3	14	2.74E-05	0.001277	0.982884	0.992645
Acetate	BFP-C4	13	-0.00172	0.00139	0.215089	0.57644
Acetate	BFP-C5	9	-9.4E-05	0.002138	0.964866	0.979802
Acetate	BMI-all	540	-0.00174	0.000233	8.74E-14	2.09E-13
Acetate	BMI-C1	39	-0.00241	0.000821	0.003387	0.008105
Acetate	BMI-C2	82	-0.00231	0.000631	0.000251	0.000646
Acetate	BMI-C3	8	-0.00034	0.002126	0.872797	0.933046
3-Hydroxybutyrate	BFP-all	388	0.001479	0.000297	6.59E-07	1.52E-06
3-Hydroxybutyrate	BFP-C1	7	0.001757	0.002488	0.480086	0.728755
3-Hydroxybutyrate	BFP-C2	101	0.001238	0.000526	0.018567	0.035043
3-Hydroxybutyrate	BFP-C3	14	-0.00063	0.001509	0.674506	0.80545
3-Hydroxybutyrate	BFP-C4	13	0.00475	0.002305	0.039339	0.225685
3-Hydroxybutyrate	BFP-C5	9	0.002449	0.002102	0.244025	0.359333
3-Hydroxybutyrate	BMI-all	540	0.001069	0.000249	1.7E-05	3.17E-05
3-Hydroxybutyrate	BMI-C1	39	-0.00055	0.001073	0.608039	0.684683
3-Hydroxybutyrate	BMI-C2	82	0.001147	0.000619	0.064052	0.110039
3-Hydroxybutyrate	BMI-C3	8	0.005578	0.004621	0.227406	0.513957
Apolipoprotein B to Apolipoprotein A1 ratio	BFP-all	388	0.001	0.000	0.000	0.000

			426	408	478	915
Apolipoprotein B to Apolipoprotein A1 ratio	BFP-C1	7	-0.00332	0.00345	0.336176	0.592731
Apolipoprotein B to Apolipoprotein A1 ratio	BFP-C2	101	0.003208	0.000702	4.87E-06	1.15E-05
Apolipoprotein B to Apolipoprotein A1 ratio	BFP-C3	14	-0.00124	0.001437	0.38657	0.602329
Apolipoprotein B to Apolipoprotein A1 ratio	BFP-C4	13	-0.00458	0.003111	0.141272	0.450724
Apolipoprotein B to Apolipoprotein A1 ratio	BFP-C5	9	-0.00831	0.004002	0.03779	0.095841
Apolipoprotein B to Apolipoprotein A1 ratio	BMI-all	540	0.00194	0.000289	1.89E-11	4.23E-11
Apolipoprotein B to Apolipoprotein A1 ratio	BMI-C1	39	0.00358	0.00145	0.01355	0.028821
Apolipoprotein B to Apolipoprotein A1 ratio	BMI-C2	82	0.002947	0.000843	0.000473	0.001132
Apolipoprotein B to Apolipoprotein A1 ratio	BMI-C3	8	-0.00482	0.003374	0.153548	0.419906
Apolipoprotein A1	BFP-all	388	-0.00332	0.000416	1.47E-15	5.81E-15
Apolipoprotein A1	BFP-C1	7	-0.00596	0.003639	0.101617	0.216137
Apolipoprotein A1	BFP-C2	101	-0.0053	0.000786	1.59E-11	5.77E-11
Apolipoprotein A1	BFP-C3	14	-0.00225	0.001547	0.145862	0.320418
Apolipoprotein A1	BFP-C4	13	0.002925	0.003088	0.343546	0.730717
Apolipoprotein A1	BFP-C5	9	0.00984	0.002911	0.000724	0.005107
Apolipoprotein A1	BMI-all	540	-0.00353	0.000298	2.38E-32	8.4E-32
Apolipoprotein A1	BMI-C1	39	-0.00702	0.000985	1.05E-12	2.81E-11
Apolipoprotein A1	BMI-C2	82	-0.00494	0.000954	2.24E-07	7.51E-07
Apolipoprotein A1	BMI-C3	8	0.005338	0.003675	0.146385	0.417352

Apolipoprotein B	BFP-all	388	-0.00059	0.000374	0.113687	0.173114
Apolipoprotein B	BFP-C1	7	-0.00781	0.004677	0.094897	0.2051
Apolipoprotein B	BFP-C2	101	0.000125	0.000721	0.862482	0.898515
Apolipoprotein B	BFP-C3	14	-0.00311	0.001332	0.01959	0.072918
Apolipoprotein B	BFP-C4	13	-0.00346	0.002294	0.131217	0.428855
Apolipoprotein B	BFP-C5	9	-0.0033	0.002924	0.258984	0.37316
Apolipoprotein B	BMI-all	540	-0.00011	0.000273	0.691806	0.747597
Apolipoprotein B	BMI-C1	39	-0.00058	0.001495	0.698501	0.767207
Apolipoprotein B	BMI-C2	82	0.000206	0.000738	0.779771	0.842656
Apolipoprotein B	BMI-C3	8	-0.00235	0.002684	0.382072	0.673653
Polyunsaturated Fatty Acids to Monounsaturated Fatty Acids ratio	BFP-all	388	-0.00531	0.000476	5.7E-29	4.25E-28
Polyunsaturated Fatty Acids to Monounsaturated Fatty Acids ratio	BFP-C1	7	-0.00875	0.002776	0.001621	0.010344
Polyunsaturated Fatty Acids to Monounsaturated Fatty Acids ratio	BFP-C2	101	-0.00823	0.000687	4.67E-33	1.25E-31
Polyunsaturated Fatty Acids to Monounsaturated Fatty Acids ratio	BFP-C3	14	-0.0059	0.001332	9.41E-06	0.00018
Polyunsaturated Fatty Acids to Monounsaturated Fatty Acids ratio	BFP-C4	13	0.004035	0.002628	0.124674	0.421274
Polyunsaturated Fatty Acids to Monounsaturated Fatty Acids ratio	BFP-C5	9	0.011622	0.004853	0.016638	0.053084
Polyunsaturated Fatty Acids to Monounsaturated Fatty Acids ratio	BMI-all	540	-0.00529	0.000318	4.42E-62	4.93E-61
Polyunsaturated Fatty Acids to Monounsaturated Fatty Acids ratio	BMI-C1	39	-0.007	0.001668	2.69E-05	9.48E-05
Polyunsaturated Fatty Acids to Monounsaturated Fatty Acids ratio	BMI-C2	82	-0.005	0.000747	1.3E-15	1.16E-14

			97			
Polyunsaturated Fatty Acids to Monounsaturated Fatty Acids ratio	BMI-C3	8	0.007225	0.001645	1.12E-05	0.000299
Polyunsaturated fatty acids	BFP-all	388	-0.00155	0.000376	3.95E-05	8.26E-05
Polyunsaturated fatty acids	BFP-C1	7	-0.00685	0.004478	0.125914	0.248124
Polyunsaturated fatty acids	BFP-C2	101	-0.00051	0.000709	0.467989	0.540608
Polyunsaturated fatty acids	BFP-C3	14	-0.00244	0.001556	0.116721	0.273897
Polyunsaturated fatty acids	BFP-C4	13	-0.00323	0.001737	0.063217	0.266607
Polyunsaturated fatty acids	BFP-C5	9	-0.00273	0.002446	0.263822	0.376087
Polyunsaturated fatty acids	BMI-all	540	-0.00091	0.000277	0.001055	0.001812
Polyunsaturated fatty acids	BMI-C1	39	-0.0019	0.00132	0.149617	0.220315
Polyunsaturated fatty acids	BMI-C2	82	-0.00138	0.000791	0.08156	0.134926
Polyunsaturated fatty acids	BMI-C3	8	-0.00279	0.001949	0.152279	0.419906
Saturated fatty acids	BFP-all	388	0.001205	0.000423	0.004408	0.007671
Saturated fatty acids	BFP-C1	7	-0.00081	0.003653	0.82525	0.987353
Saturated fatty acids	BFP-C2	101	0.003572	0.00063	1.42E-08	3.97E-08
Saturated fatty acids	BFP-C3	14	0.000942	0.001476	0.52346	0.708522
Saturated fatty acids	BFP-C4	13	-0.0053	0.001608	0.000989	0.014728
Saturated fatty acids	BFP-C5	9	-0.00942	0.003957	0.017285	0.053864
Saturated fatty acids	BMI-all	540	0.00164	0.000267	7.55E-10	1.63E-09
Saturated fatty acids	BMI-C1	39	0.001	0.001	0.344	0.462

			314	391	808	042
Saturated fatty acids	BMI-C2	82	0.001575	0.000775	0.042156	0.076337
Saturated fatty acids	BMI-C3	8	-0.00613	0.001666	0.000233	0.003123
Monounsaturated fatty acids	BFP-all	388	0.002853	0.000457	4.28E-10	1.17E-09
Monounsaturated fatty acids	BFP-C1	7	0.002211	0.002975	0.457335	0.718007
Monounsaturated fatty acids	BFP-C2	101	0.005652	0.000641	1.21E-18	8.11E-18
Monounsaturated fatty acids	BFP-C3	14	0.002574	0.00147	0.079985	0.218736
Monounsaturated fatty acids	BFP-C4	13	-0.00494	0.002059	0.016439	0.110141
Monounsaturated fatty acids	BFP-C5	9	-0.01027	0.004739	0.030172	0.08251
Monounsaturated fatty acids	BMI-all	540	0.003224	0.000295	1E-27	3.21E-27
Monounsaturated fatty acids	BMI-C1	39	0.004001	0.00167	0.01655	0.033601
Monounsaturated fatty acids	BMI-C2	82	0.003442	0.00078	1.03E-05	2.82E-05
Monounsaturated fatty acids	BMI-C3	8	-0.00708	0.001745	4.9E-05	0.000939
Degree of unsaturation	BFP-all	388	-0.00363	0.000376	5.15E-22	3.14E-21
Degree of unsaturation	BFP-C1	7	-0.00702	0.002735	0.010301	0.044526
Degree of unsaturation	BFP-C2	101	-0.00558	0.000613	8.94E-20	7.05E-19
Degree of unsaturation	BFP-C3	14	-0.00171	0.001713	0.317159	0.537966
Degree of unsaturation	BFP-C4	13	0.001285	0.001997	0.519948	0.870913
Degree of unsaturation	BFP-C5	9	0.007727	0.003126	0.013438	0.047388
Degree of unsaturation	BMI-all	540	-0.00357	0.000294	8.54E-34	3.37E-33
Degree of unsaturation	BMI-C1	39	-0.00414	0.001238	0.000834	0.002234

Degree of unsaturation	BMI-C2	82	- 0.003 96	0.000 663	2.28E -09	9.86E -09
Degree of unsaturation	BMI-C3	8	0.004 587	0.001 6	0.004 142	0.032 649
Omega-6 Fatty Acids to Omega-3 Fatty Acids ratio	BFP-all	388	0.000 698	0.000 353	0.047 822	0.076 288
Omega-6 Fatty Acids to Omega-3 Fatty Acids ratio	BFP-C1	7	- 0.001 62	0.001 735	0.351 737	0.610 109
Omega-6 Fatty Acids to Omega-3 Fatty Acids ratio	BFP-C2	101	- 0.000 53	0.000 593	0.375 182	0.452 923
Omega-6 Fatty Acids to Omega-3 Fatty Acids ratio	BFP-C3	14	- 0.001 04	0.002 391	0.664 861	0.805 45
Omega-6 Fatty Acids to Omega-3 Fatty Acids ratio	BFP-C4	13	0.006 759	0.002 135	0.001 549	0.020 75
Omega-6 Fatty Acids to Omega-3 Fatty Acids ratio	BFP-C5	9	0.008 921	0.002 312	0.000 114	0.001 524
Omega-6 Fatty Acids to Omega-3 Fatty Acids ratio	BMI-all	540	2.24E -05	0.000 263	0.931 954	0.946 074
Omega-6 Fatty Acids to Omega-3 Fatty Acids ratio	BMI-C1	39	- 0.001 01	0.000 877	0.247 028	0.344 81
Omega-6 Fatty Acids to Omega-3 Fatty Acids ratio	BMI-C2	82	0.000 263	0.000 638	0.680 375	0.766 886
Omega-6 Fatty Acids to Omega-3 Fatty Acids ratio	BMI-C3	8	0.006 105	0.003 982	0.125 187	0.371 824
Linoleic acid	BFP-all	388	- 0.002 2	0.000 354	5.29E -10	1.42E -09
Linoleic acid	BFP-C1	7	- 0.007 91	0.004 622	0.087 158	0.197 953
Linoleic acid	BFP-C2	101	- 0.002 22	0.000 735	0.002 572	0.004 994
Linoleic acid	BFP-C3	14	- 0.004 12	0.001 198	0.000 595	0.004 448
Linoleic acid	BFP-C4	13	- 0.001 45	0.001 905	0.446 622	0.808 747
Linoleic acid	BFP-C5	9	- 0.000 7	0.002 009	0.725 703	0.803 671
Linoleic acid	BMI-all	540	- 0.001 53	0.000 274	2.42E -08	5.07E -08
Linoleic acid	BMI-C1	39	-	0.001	0.032	0.060

			0.0028	308	415	328
Linoleic acid	BMI-C2	82	-0.00202	0.000754	0.007463	0.016129
Linoleic acid	BMI-C3	8	-0.00175	0.002229	0.431533	0.722818
Omega-6 fatty acids	BFP-all	388	-0.00142	0.000364	9.38E-05	0.00019
Omega-6 fatty acids	BFP-C1	7	-0.0074	0.004644	0.11107	0.232553
Omega-6 fatty acids	BFP-C2	101	-0.00063	0.000743	0.396362	0.470022
Omega-6 fatty acids	BFP-C3	14	-0.00257	0.001331	0.053873	0.160423
Omega-6 fatty acids	BFP-C4	13	-0.00165	0.00192	0.391488	0.760282
Omega-6 fatty acids	BFP-C5	9	-0.00052	0.002115	0.807501	0.872622
Omega-6 fatty acids	BMI-all	540	-0.00094	0.000275	0.000635	0.00112
Omega-6 fatty acids	BMI-C1	39	-0.00223	0.001328	0.092461	0.147498
Omega-6 fatty acids	BMI-C2	82	-0.0014	0.00079	0.075783	0.128544
Omega-6 fatty acids	BMI-C3	8	-0.00133	0.002272	0.559016	0.780292
Docosahexaenoic acid	BFP-all	388	-0.00301	0.000324	1.47E-20	8.19E-20
Docosahexaenoic acid	BFP-C1	7	-0.00534	0.002231	0.016565	0.065286
Docosahexaenoic acid	BFP-C2	101	-0.00344	0.000623	3.47E-08	9.31E-08
Docosahexaenoic acid	BFP-C3	14	-0.00038	0.002168	0.859977	0.94324
Docosahexaenoic acid	BFP-C4	13	-	0.001	0.012	0.091

			0.004<br>07	637	977	519
Docosahexaenoic acid	BFP-C5	9	-<br>0.000<br>89	0.001<br>474	0.547<br>414	0.637<br>856
Docosahexaenoic acid	BMI-all	540	-<br>0.002<br>57	0.000<br>27	1.37E<br>-21	3.66E<br>-21
Docosahexaenoic acid	BMI-C1	39	-<br>0.003<br>54	0.000<br>927	0.000<br>134	0.000<br>399
Docosahexaenoic acid	BMI-C2	82	-<br>0.003<br>11	0.000<br>669	3.46E<br>-06	9.87E<br>-06
Docosahexaenoic acid	BMI-C3	8	-<br>0.001<br>66	0.002<br>367	0.483<br>371	0.738<br>22
Omega-3 fatty acids	BFP-all	388	-<br>0.001<br>14	0.000<br>372	0.002<br>095	0.003<br>845
Omega-3 fatty acids	BFP-C1	7	-<br>0.001<br>53	0.002<br>213	0.488<br>411	0.728<br>755
Omega-3 fatty acids	BFP-C2	101	1.82E<br>-05	0.000<br>582	0.975<br>07	0.975<br>07
Omega-3 fatty acids	BFP-C3	14	-2.2E-<br>05	0.002<br>36	0.992<br>645	0.992<br>645
Omega-3 fatty acids	BFP-C4	13	-<br>0.006<br>71	0.001<br>802	0.000<br>199	0.003<br>808
Omega-3 fatty acids	BFP-C5	9	-<br>0.008<br>23	0.002<br>731	0.002<br>575	0.012<br>169
Omega-3 fatty acids	BMI-all	540	-<br>0.000<br>36	0.000<br>268	0.177<br>219	0.242<br>32
Omega-3 fatty acids	BMI-C1	39	2.05E<br>-05	0.000<br>989	0.983<br>449	0.988<br>552
Omega-3 fatty acids	BMI-C2	82	-<br>0.000<br>72	0.000<br>696	0.302<br>308	0.401<br>082
Omega-3 fatty acids	BMI-C3	8	-<br>0.005<br>77	0.003<br>285	0.078<br>869	0.285<br>632
Glycoprotein acetyls	BFP-all	388	0.005<br>463	0.000<br>399	1.37E<br>-42	1.31E<br>-41
Glycoprotein acetyls	BFP-C1	7	0.005<br>417	0.001<br>745	0.001<br>91	0.011<br>589
Glycoprotein acetyls	BFP-C2	101	0.008<br>405	0.000<br>564	3.68E<br>-50	2.47E<br>-48
Glycoprotein acetyls	BFP-C3	14	0.003	0.001	0.064	0.184

			49	89	847	884
Glycoprotein acetyls	BFP-C4	13	0.000831	0.002424	0.731856	0.935741
Glycoprotein acetyls	BFP-C5	9	-0.00552	0.00357	0.122327	0.207492
Glycoprotein acetyls	BMI-all	540	0.004802	0.000328	1.27E-48	6.53E-48
Glycoprotein acetyls	BMI-C1	39	0.006039	0.001335	6.08E-06	2.4E-05
Glycoprotein acetyls	BMI-C2	82	0.005826	0.000814	8.1E-13	5.2E-12
Glycoprotein acetyls	BMI-C3	8	-0.00536	0.002493	0.031557	0.156616
Citrate	BFP-all	388	-0.00037	0.0003	0.21892	0.287601
Citrate	BFP-C1	7	0.000848	0.001741	0.626235	0.822701
Citrate	BFP-C2	101	-0.00089	0.00053	0.091324	0.145992
Citrate	BFP-C3	14	0.000713	0.001923	0.710841	0.825833
Citrate	BFP-C4	13	-0.00043	0.001436	0.762841	0.935741
Citrate	BFP-C5	9	0.00118	0.001961	0.547138	0.637856
Citrate	BMI-all	540	-0.00101	0.000244	3.84E-05	7.06E-05
Citrate	BMI-C1	39	-0.00227	0.001035	0.028515	0.055355
Citrate	BMI-C2	82	-0.00083	0.000593	0.163003	0.251062
Citrate	BMI-C3	8	-0.00205	0.003326	0.536758	0.763831
Pyruvate	BFP-all	388	0.001681	0.000278	1.54E-09	4.05E-09
Pyruvate	BFP-C1	7	0.000735	0.001933	0.703799	0.905769
Pyruvate	BFP-C2	101	0.001825	0.000528	0.000545	0.001178
Pyruvate	BFP-C3	14	0.00191	0.001187	0.107605	0.260487

Pyruvate	BFP-C4	13	0.000369	0.002043	0.856566	0.96189
Pyruvate	BFP-C5	9	0.003524	0.001894	0.062743	0.129348
Pyruvate	BMI-all	540	0.00108	0.000248	1.35E-05	2.55E-05
Pyruvate	BMI-C1	39	0.00169	0.000935	0.070658	0.12458
Pyruvate	BMI-C2	82	0.001502	0.000626	0.01645	0.032417
Pyruvate	BMI-C3	8	-0.00018	0.003086	0.954739	0.976603
Lactate	BFP-all	388	0.00108	0.000279	0.00011	0.000217
Lactate	BFP-C1	7	0.003565	0.001866	0.056072	0.147327
Lactate	BFP-C2	101	0.001686	0.000499	0.000729	0.00155
Lactate	BFP-C3	14	0.00032	0.001439	0.82394	0.93566
Lactate	BFP-C4	13	-0.00034	0.001559	0.829074	0.957723
Lactate	BFP-C5	9	-0.00223	0.001596	0.163069	0.254084
Lactate	BMI-all	540	0.000845	0.00025	0.000732	0.001274
Lactate	BMI-C1	39	0.001736	0.000807	0.031503	0.059457
Lactate	BMI-C2	82	0.000563	0.000647	0.384292	0.495146
Lactate	BMI-C3	8	-0.00431	0.002692	0.109201	0.355654
Glucose	BFP-all	388	0.001967	0.000305	1.12E-10	3.32E-10
Glucose	BFP-C1	7	0.004085	0.002291	0.074586	0.175096
Glucose	BFP-C2	101	0.003355	0.000524	1.56E-10	5.1E-10
Glucose	BFP-C3	14	0.001297	0.001424	0.36243	0.578162
Glucose	BFP-C4	13	-0.00135	0.00161	0.400244	0.766181
Glucose	BFP-C5	9	-0.00793	0.002948	0.007145	0.02816
Glucose	BMI-all	540	0.001	0.000	9.88E	2.24E

			756	246	-13	-12
Glucose	BMI-C1	39	0.004 394	0.000 862	3.49E -07	1.73E -06
Glucose	BMI-C2	82	0.001 547	0.000 665	0.020 03	0.038 9
Glucose	BMI-C3	8	0.000 44	0.002 086	0.833 065	0.915 005
Histidine	BFP-all	388	- 0.000 88	0.000 261	0.000 765	0.001 444
Histidine	BFP-C1	7	0.001 208	0.001 758	0.491 996	0.728 755
Histidine	BFP-C2	101	- 0.000 65	0.000 554	0.240 397	0.312 749
Histidine	BFP-C3	14	- 0.000 25	0.001 17	0.831 578	0.936 399
Histidine	BFP-C4	13	- 0.001 49	0.001 504	0.322 348	0.729 053
Histidine	BFP-C5	9	- 0.003	0.001 811	0.097 293	0.171 542
Histidine	BMI-all	540	0.000 139	0.000 23	0.545 449	0.619 408
Histidine	BMI-C1	39	0.000 316	0.000 887	0.722 064	0.786 638
Histidine	BMI-C2	82	- 0.000 66	0.000 535	0.215 526	0.317 368
Histidine	BMI-C3	8	- 0.003 74	0.003 201	0.242 081	0.523 207
Glycine	BFP-all	388	- 0.002 83	0.000 377	6.93E -14	2.38E -13
Glycine	BFP-C1	7	- 0.004 42	0.001 898	0.019 898	0.066 33
Glycine	BFP-C2	101	- 0.004 78	0.000 52	3.68E -20	3.08E -19
Glycine	BFP-C3	14	- 0.006 22	0.002 525	0.013 842	0.054 555
Glycine	BFP-C4	13	0.000 56	0.002 387	0.814 566	0.957 473
Glycine	BFP-C5	9	0.010 701	0.004 551	0.018 721	0.057 013
Glycine	BMI-all	540	- 0.002	0.000 251	2.31E -24	6.72E -24

			56			
Glycine	BMI-C1	39	-0.00379	0.00097	9.44E-05	0.00029
Glycine	BMI-C2	82	-0.00343	0.000637	6.89E-08	2.43E-07
Glycine	BMI-C3	8	0.001901	0.002533	0.452966	0.726175
Glutamine	BFP-all	388	-0.00219	0.000345	2.31E-10	6.74E-10
Glutamine	BFP-C1	7	-0.00274	0.001781	0.123886	0.248124
Glutamine	BFP-C2	101	-0.00412	0.000613	1.77E-11	6.23E-11
Glutamine	BFP-C3	14	-0.00139	0.002149	0.518913	0.708522
Glutamine	BFP-C4	13	-0.00061	0.002297	0.789335	0.944383
Glutamine	BFP-C5	9	0.003099	0.004799	0.518431	0.637337
Glutamine	BMI-all	540	-0.002	0.000275	3.4E-13	8E-13
Glutamine	BMI-C1	39	-0.00269	0.001163	0.020878	0.041142
Glutamine	BMI-C2	82	-0.00214	0.000806	0.007799	0.016589
Glutamine	BMI-C3	8	-0.0031	0.003068	0.311951	0.605818
Alanine	BFP-all	388	0.000368	0.000328	0.262638	0.328002
Alanine	BFP-C1	7	0.002687	0.001738	0.122112	0.248124
Alanine	BFP-C2	101	0.001825	0.000591	0.002005	0.00407
Alanine	BFP-C3	14	0.000945	0.00116	0.415464	0.630391
Alanine	BFP-C4	13	-0.00286	0.001868	0.125753	0.421274
Alanine	BFP-C5	9	-0.00891	0.002536	0.000441	0.003691

Alanine	BMI-all	540	0.000487	0.000268	0.068529	0.104351
Alanine	BMI-C1	39	0.003238	0.001094	0.003086	0.007658
Alanine	BMI-C2	82	-0.00055	0.000657	0.403218	0.514582
Alanine	BMI-C3	8	-0.00135	0.002411	0.576054	0.795787
Valine	BFP-all	388	0.002898	0.00036	8.44E-16	3.65E-15
Valine	BFP-C1	7	0.008229	0.002138	0.000119	0.001989
Valine	BFP-C2	101	0.004811	0.000513	7.1E-21	6.34E-20
Valine	BFP-C3	14	0.002286	0.001426	0.10886	0.260487
Valine	BFP-C4	13	-0.0021	0.002138	0.326851	0.729053
Valine	BFP-C5	9	-0.0107	0.003628	0.003189	0.014245
Valine	BMI-all	540	0.003929	0.000284	1.74E-43	8.32E-43
Valine	BMI-C1	39	0.006852	0.001136	1.61E-09	1.97E-08
Valine	BMI-C2	82	0.003868	0.000688	1.9E-08	7.08E-08
Valine	BMI-C3	8	-0.00426	0.002798	0.127641	0.371824
Leucine	BFP-all	388	0.002062	0.000327	2.96E-10	8.27E-10
Leucine	BFP-C1	7	0.006002	0.002062	0.003607	0.017264
Leucine	BFP-C2	101	0.003213	0.000497	1.03E-10	3.46E-10
Leucine	BFP-C3	14	0.001366	0.001118	0.221787	0.412769
Leucine	BFP-C4	13	-0.00233	0.001955	0.233362	0.59001
Leucine	BFP-C5	9	-0.00839	0.003491	0.016252	0.053084
Leucine	BMI-all	540	0.003238	0.000276	8.22E-32	2.82E-31
Leucine	BMI-C1	39	0.005321	0.001055	4.59E-07	2.2E-06

Leucine	BMI-C2	82	0.00329	0.000657	5.56E-07	1.82E-06
Leucine	BMI-C3	8	-0.00388	0.002435	0.111474	0.355654
Isoleucine	BFP-all	388	0.002384	0.000323	1.46E-13	4.77E-13
Isoleucine	BFP-C1	7	0.006753	0.002137	0.001575	0.010344
Isoleucine	BFP-C2	101	0.003472	0.000487	1.01E-12	4.1E-12
Isoleucine	BFP-C3	14	0.001733	0.001145	0.129959	0.290242
Isoleucine	BFP-C4	13	-0.00188	0.001958	0.337323	0.729053
Isoleucine	BFP-C5	9	-0.00703	0.00346	0.042264	0.102971
Isoleucine	BMI-all	540	0.003231	0.000259	1.18E-35	4.94E-35
Isoleucine	BMI-C1	39	0.006046	0.001065	1.39E-08	1.09E-07
Isoleucine	BMI-C2	82	0.003397	0.000651	1.78E-07	6.11E-07
Isoleucine	BMI-C3	8	-0.00351	0.002157	0.104124	0.355654
Tyrosine	BFP-all	388	0.002853	0.000345	1.47E-16	6.8E-16
Tyrosine	BFP-C1	7	0.008279	0.00235	0.000426	0.005284
Tyrosine	BFP-C2	101	0.003463	0.000595	5.75E-09	1.68E-08
Tyrosine	BFP-C3	14	0.003744	0.001469	0.010821	0.043939
Tyrosine	BFP-C4	13	0.001925	0.002148	0.370203	0.740406
Tyrosine	BFP-C5	9	-0.00111	0.002487	0.656325	0.732896
Tyrosine	BMI-all	540	0.002776	0.000276	8E-24	2.19E-23
Tyrosine	BMI-C1	39	0.005956	0.001351	1.04E-05	3.88E-05
Tyrosine	BMI-C2	82	0.002732	0.00078	0.000458	0.001115
Tyrosine	BMI-C3	8	-0.00074	0.002714	0.784032	0.881473
Phenylalanine	BFP-all	388	0.002	0.000	7.08E	2.71E

			061	265	-15	-14
Phenylalanine	BFP-C1	7	0.006929	0.002082	0.000876	0.007828
Phenylalanine	BFP-C2	101	0.002579	0.000463	2.6E-08	7.11E-08
Phenylalanine	BFP-C3	14	0.001684	0.001167	0.148952	0.321928
Phenylalanine	BFP-C4	13	3.37E-05	0.001293	0.97923	0.986593
Phenylalanine	BFP-C5	9	0.000912	0.002002	0.648709	0.730479
Phenylalanine	BMI-all	540	0.002333	0.000219	1.93E-26	5.75E-26
Phenylalanine	BMI-C1	39	0.005213	0.000879	3.01E-09	2.88E-08
Phenylalanine	BMI-C2	82	0.002069	0.000623	0.000904	0.002089
Phenylalanine	BMI-C3	8	1.65E-05	0.001698	0.992244	0.992244
b-NGF	BFP-all	381	0.00089	0.001435	0.535078	0.602525
b-NGF	BFP-C1	7	0.000263	0.010899	0.980758	0.999247
b-NGF	BFP-C2	99	0.00193	0.002685	0.472108	0.540705
b-NGF	BFP-C3	14	-0.0033	0.006721	0.62374	0.782955
b-NGF	BFP-C4	12	-0.00302	0.009604	0.752874	0.935741
b-NGF	BFP-C5	9	0.000293	0.008489	0.97249	0.979802
b-NGF	BMI-all	538	0.003044	0.001185	0.010209	0.016889
b-NGF	BMI-C1	39	0.003947	0.004251	0.353171	0.468564
b-NGF	BMI-C2	81	0.004087	0.003806	0.282821	0.379011
b-NGF	BMI-C3	8	-0.00353	0.009831	0.719402	0.880301
CCL11/Eotaxin	BFP-all	383	-0.00119	0.000932	0.202965	0.26928
CCL11/Eotaxin	BFP-C1	7	-0.00712	0.010807	0.510282	0.735245
CCL11/Eotaxin	BFP-C2	99	0.000311	0.001829	0.864988	0.898515

CCL11/Eotaxin	BFP-C3	14	0.000345	0.004452	0.93823	0.989943
CCL11/Eotaxin	BFP-C4	13	-0.01394	0.005299	0.008499	0.075927
CCL11/Eotaxin	BFP-C5	9	-0.01055	0.005585	0.058806	0.12508
CCL11/Eotaxin	BMI-all	539	-0.00134	0.000792	0.090688	0.132088
CCL11/Eotaxin	BMI-C1	39	-0.00214	0.002814	0.446951	0.549463
CCL11/Eotaxin	BMI-C2	81	-0.0035	0.002249	0.119301	0.192606
CCL11/Eotaxin	BMI-C3	8	0.008521	0.006491	0.189288	0.478503
CCL2/MCP1	BFP-all	384	0.003024	0.000982	0.00208	0.003845
CCL2/MCP1	BFP-C1	7	0.002515	0.008763	0.774151	0.943057
CCL2/MCP1	BFP-C2	100	0.004931	0.001969	0.012262	0.023472
CCL2/MCP1	BFP-C3	14	0.005353	0.004419	0.225754	0.414398
CCL2/MCP1	BFP-C4	13	-0.00045	0.004896	0.927209	0.965131
CCL2/MCP1	BFP-C5	9	-0.0045	0.005547	0.417387	0.548332
CCL2/MCP1	BMI-all	539	0.002413	0.000809	0.002835	0.004809
CCL2/MCP1	BMI-C1	39	0.004738	0.002797	0.090221	0.147434
CCL2/MCP1	BMI-C2	81	0.004762	0.002054	0.0204	0.039051
CCL2/MCP1	BMI-C3	8	-0.00012	0.01154	0.992005	0.992244
CCL27/CTACK	BFP-all	381	-0.00411	0.001379	0.002878	0.005211
CCL27/CTACK	BFP-C1	7	1.53E-05	0.016237	0.999247	0.999247
CCL27/CTACK	BFP-C2	99	-0.00425	0.00264	0.107102	0.166879
CCL27/CTACK	BFP-C3	14	-	0.007	0.308	0.530

			0.00748	347	847	584
CCL27/CTACK	BFP-C4	12	-0.00732	0.009849	0.457289	0.815913
CCL27/CTACK	BFP-C5	9	0.013645	0.010543	0.195576	0.294463
CCL27/CTACK	BMI-all	539	-0.00435	0.00121	0.000328	0.000585
CCL27/CTACK	BMI-C1	39	-0.0122	0.004533	0.007134	0.015932
CCL27/CTACK	BMI-C2	81	-0.00061	0.003201	0.848941	0.888735
CCL27/CTACK	BMI-C3	8	0.014956	0.012928	0.247335	0.523263
CCL3/MIP-1-a	BFP-all	382	0.001328	0.001463	0.364223	0.431911
CCL3/MIP-1-a	BFP-C1	7	0.000296	0.013358	0.982304	0.999247
CCL3/MIP-1-a	BFP-C2	99	0.003562	0.002786	0.201049	0.269406
CCL3/MIP-1-a	BFP-C3	14	-0.00021	0.008023	0.979535	0.992645
CCL3/MIP-1-a	BFP-C4	12	-0.00665	0.009985	0.505323	0.85713
CCL3/MIP-1-a	BFP-C5	9	-0.01046	0.008466	0.216654	0.322573
CCL3/MIP-1-a	BMI-all	539	0.000258	0.001173	0.825868	0.871334
CCL3/MIP-1-a	BMI-C1	39	0.007579	0.004381	0.083636	0.143683
CCL3/MIP-1-a	BMI-C2	81	-0.0008	0.003307	0.80917	0.85423
CCL3/MIP-1-a	BMI-C3	8	-0.00647	0.010239	0.527407	0.763831
CCL4/MIP-1-b	BFP-all	384	0.001696	0.000927	0.067402	0.106257
CCL4/MIP-1-b	BFP-C1	7	0.003136	0.010035	0.754653	0.932726
CCL4/MIP-1-b	BFP-C2	100	0.002602	0.001938	0.179312	0.247709
CCL4/MIP-1-b	BFP-C3	14	0.000746	0.004414	0.86581	0.94324

CCL4/MIP-1-b	BFP-C4	13	0.002227	0.005	0.656003	0.899436
CCL4/MIP-1-b	BFP-C5	9	-0.00947	0.005565	0.088847	0.160886
CCL4/MIP-1-b	BMI-all	539	0.000559	0.000866	0.518793	0.614024
CCL4/MIP-1-b	BMI-C1	39	0.00472	0.002797	0.091467	0.147498
CCL4/MIP-1-b	BMI-C2	81	-5.6E-05	0.002347	0.981116	0.988493
CCL4/MIP-1-b	BMI-C3	8	-0.0068	0.015189	0.65461	0.835407
CCL5/RANTES	BFP-all	382	0.000828	0.00142	0.5596	0.619722
CCL5/RANTES	BFP-C1	7	-0.0037	0.009952	0.709745	0.905769
CCL5/RANTES	BFP-C2	99	0.001439	0.002726	0.597491	0.650926
CCL5/RANTES	BFP-C3	14	0.011427	0.006821	0.093873	0.241903
CCL5/RANTES	BFP-C4	12	0.010136	0.007842	0.19615	0.547585
CCL5/RANTES	BFP-C5	9	0.00103	0.012105	0.932218	0.968351
CCL5/RANTES	BMI-all	539	-0.00076	0.001189	0.521146	0.614024
CCL5/RANTES	BMI-C1	39	0.002428	0.004323	0.574299	0.67261
CCL5/RANTES	BMI-C2	81	-0.00083	0.003182	0.793851	0.851008
CCL5/RANTES	BMI-C3	8	-0.00883	0.010001	0.377338	0.673653
CCL7/MCP3	BFP-all	375	0.00309	0.002578	0.230761	0.297327
CCL7/MCP3	BFP-C1	7	0.006008	0.018168	0.740863	0.92781
CCL7/MCP3	BFP-C2	98	0.010263	0.005076	0.04317	0.073226
CCL7/MCP3	BFP-C3	14	-0.01707	0.012079	0.15759	0.335191
CCL7/MCP3	BFP-C4	12	0.017056	0.018696	0.361606	0.740406
CCL7/MCP3	BFP-C5	9	0.015635	0.01788	0.381873	0.522153

CCL7/MCP3	BMI-all	534	0.004889	0.002118	0.020988	0.033087
CCL7/MCP3	BMI-C1	39	0.004891	0.009134	0.592298	0.67261
CCL7/MCP3	BMI-C2	79	0.002975	0.00591	0.614756	0.710149
CCL7/MCP3	BMI-C3	8	0.037803	0.022263	0.089512	0.315649
CXCL1/GRO-a	BFP-all	381	0.00036	0.001398	0.796648	0.827526
CXCL1/GRO-a	BFP-C1	7	0.004906	0.00981	0.617035	0.822701
CXCL1/GRO-a	BFP-C2	99	0.003094	0.002684	0.248958	0.320773
CXCL1/GRO-a	BFP-C3	14	-0.01465	0.006722	0.029283	0.097315
CXCL1/GRO-a	BFP-C4	12	-0.0037	0.008344	0.657797	0.899436
CXCL1/GRO-a	BFP-C5	9	-0.01714	0.008485	0.043423	0.10337
CXCL1/GRO-a	BMI-all	539	0.000552	0.001175	0.638292	0.71276
CXCL1/GRO-a	BMI-C1	39	0.009173	0.004548	0.04369	0.080199
CXCL1/GRO-a	BMI-C2	81	0.00338	0.003131	0.280421	0.379011
CXCL1/GRO-a	BMI-C3	8	-0.00349	0.009843	0.722635	0.880301
CXCL10/IP10	BFP-all	383	0.000609	0.001387	0.660668	0.713948
CXCL10/IP10	BFP-C1	7	0.008771	0.00958	0.359907	0.610476
CXCL10/IP10	BFP-C2	100	0.003181	0.002624	0.225395	0.299039
CXCL10/IP10	BFP-C3	14	-0.01025	0.007398	0.165831	0.337075
CXCL10/IP10	BFP-C4	12	-0.00132	0.009445	0.888841	0.96189
CXCL10/IP10	BFP-C5	9	-0.01897	0.008296	0.022227	0.06515
CXCL10/IP10	BMI-all	539	0.002069	0.001215	0.088587	0.130447
CXCL10/IP10	BMI-C1	39	-0.000	0.004907	0.978702	0.988552

			13			
CXCL10/IP10	BMI-C2	81	0.007309	0.003457	0.034498	0.064205
CXCL10/IP10	BMI-C3	8	0.010905	0.016011	0.495819	0.73822
CXCL12/SDF1-a	BFP-all	384	0.001279	0.000966	0.185433	0.253551
CXCL12/SDF1-a	BFP-C1	7	0.005024	0.007974	0.528665	0.753628
CXCL12/SDF1-a	BFP-C2	100	0.000683	0.001855	0.712599	0.763906
CXCL12/SDF1-a	BFP-C3	14	-0.00361	0.004675	0.440468	0.648601
CXCL12/SDF1-a	BFP-C4	13	0.000681	0.005046	0.892693	0.96189
CXCL12/SDF1-a	BFP-C5	9	-0.01392	0.00635	0.028317	0.079053
CXCL12/SDF1-a	BMI-all	539	-6.3E-05	0.000833	0.939805	0.946871
CXCL12/SDF1-a	BMI-C1	39	-0.00368	0.002883	0.201641	0.287445
CXCL12/SDF1-a	BMI-C2	81	0.000751	0.00255	0.768303	0.837012
CXCL12/SDF1-a	BMI-C3	8	0.010219	0.011094	0.356965	0.664352
CXCL8/IL-8	BFP-all	381	0.001918	0.001394	0.1691	0.23852
CXCL8/IL-8	BFP-C1	7	0.001417	0.013024	0.913391	0.999247
CXCL8/IL-8	BFP-C2	99	0.003932	0.002679	0.142226	0.209432
CXCL8/IL-8	BFP-C3	14	0.002728	0.006731	0.685234	0.80545
CXCL8/IL-8	BFP-C4	12	-0.0034	0.009726	0.726806	0.935741
CXCL8/IL-8	BFP-C5	9	-0.01198	0.008461	0.156674	0.251157
CXCL8/IL-8	BMI-all	539	0.001949	0.001168	0.095272	0.137274
CXCL8/IL-8	BMI-C1	39	0.008043	0.004243	0.057987	0.103604
CXCL8/IL-8	BMI-C2	81	-0.00175	0.003121	0.575828	0.68573
CXCL8/IL-8	BMI-C3	8	-0.005	0.009814	0.596103	0.805326

			2			
CXCL9/MIG	BFP-all	383	-0.00185	0.001384	0.182129	0.251601
CXCL9/MIG	BFP-C1	7	-0.00033	0.009565	0.972092	0.999247
CXCL9/MIG	BFP-C2	100	-0.00086	0.002622	0.74216	0.789282
CXCL9/MIG	BFP-C3	14	-0.00474	0.00772	0.538983	0.722238
CXCL9/MIG	BFP-C4	12	-8.5E-05	0.009631	0.992981	0.992981
CXCL9/MIG	BFP-C5	9	-0.00481	0.008276	0.561368	0.648477
CXCL9/MIG	BMI-all	539	0.000243	0.00115	0.832319	0.871334
CXCL9/MIG	BMI-C1	39	-0.00224	0.00415	0.59019	0.67261
CXCL9/MIG	BMI-C2	81	0.003573	0.003092	0.247761	0.349474
CXCL9/MIG	BMI-C3	8	0.00066	0.009589	0.945098	0.974178
FGF-basic	BFP-all	383	0.001161	0.000997	0.244588	0.312141
FGF-basic	BFP-C1	7	-0.0048	0.009092	0.597309	0.808479
FGF-basic	BFP-C2	99	0.002744	0.002015	0.173188	0.244287
FGF-basic	BFP-C3	14	-0.00372	0.004604	0.418693	0.630391
FGF-basic	BFP-C4	13	-0.00327	0.006195	0.597396	0.899436
FGF-basic	BFP-C5	9	-0.01122	0.005791	0.052724	0.117751
FGF-basic	BMI-all	539	-0.00053	0.000821	0.522378	0.614024
FGF-basic	BMI-C1	39	0.001748	0.003185	0.583082	0.67261
FGF-basic	BMI-C2	81	-0.00103	0.002172	0.634581	0.726785

FGF-basic	BMI-C3	8	0.002329	0.008389	0.78127	0.881473
GCSF	BFP-all	383	0.001007	0.000938	0.283386	0.345216
GCSF	BFP-C1	7	-0.0112	0.00667	0.093076	0.204462
GCSF	BFP-C2	99	0.001697	0.002048	0.407262	0.474549
GCSF	BFP-C3	14	-0.0002	0.004512	0.965061	0.992645
GCSF	BFP-C4	13	-0.00117	0.006014	0.845675	0.960343
GCSF	BFP-C5	9	-0.00996	0.005653	0.078011	0.151436
GCSF	BMI-all	539	-0.00054	0.000785	0.492082	0.599445
GCSF	BMI-C1	39	-4.4E-05	0.003075	0.988552	0.988552
GCSF	BMI-C2	81	-0.00308	0.002122	0.146386	0.230773
GCSF	BMI-C3	8	-0.00239	0.006584	0.716249	0.880301
HGF	BFP-all	384	0.004338	0.00096	6.22E-06	1.37E-05
HGF	BFP-C1	7	0.013458	0.006488	0.038062	0.104088
HGF	BFP-C2	100	0.006179	0.001851	0.000842	0.001763
HGF	BFP-C3	14	-0.00085	0.004408	0.846613	0.94324
HGF	BFP-C4	13	0.004371	0.005383	0.416817	0.779045
HGF	BFP-C5	9	-0.01205	0.006911	0.081205	0.151436
HGF	BMI-all	539	0.004517	0.000775	5.51E-09	1.17E-08
HGF	BMI-C1	39	0.009169	0.003457	0.007996	0.017565
HGF	BMI-C2	81	0.004275	0.002157	0.047525	0.084911
HGF	BMI-C3	8	-0.000	0.006684	0.891268	0.933046

			91			
IFN-g	BFP-all	384	0.001 467	0.000 976	0.132 808	0.195 563
IFN-g	BFP-C1	7	- 0.006 4	0.011 196	0.567 373	0.775 796
IFN-g	BFP-C2	100	0.002 028	0.002 009	0.312 68	0.387 955
IFN-g	BFP-C3	14	0.001 696	0.004 642	0.714 9	0.825 833
IFN-g	BFP-C4	13	- 0.001 38	0.005 079	0.786 358	0.944 383
IFN-g	BFP-C5	9	- 0.012 96	0.005 753	0.024 285	0.069 237
IFN-g	BMI-all	539	0.000 123	0.000 817	0.880 486	0.907 578
IFN-g	BMI-C1	39	- 0.000 41	0.003 105	0.895 446	0.922 998
IFN-g	BMI-C2	81	- 0.001 5	0.002 195	0.494 813	0.613 934
IFN-g	BMI-C3	8	0.001 889	0.011 476	0.869 278	0.933 046
IL-1-b	BFP-all	381	0.001 662	0.001 489	0.264 36	0.328 002
IL-1-b	BFP-C1	7	- 0.015 72	0.013 891	0.257 791	0.466 811
IL-1-b	BFP-C2	99	0.004 314	0.002 772	0.119 735	0.182 324
IL-1-b	BFP-C3	14	0.012 028	0.009 48	0.204 529	0.386 013
IL-1-b	BFP-C4	12	- 0.001 61	0.007 973	0.840 076	0.960 343
IL-1-b	BFP-C5	9	- 0.007 12	0.008 767	0.416 836	0.548 332
IL-1-b	BMI-all	539	0.001 659	0.001 208	0.169 562	0.234 801
IL-1-b	BMI-C1	39	0.008 775	0.004 391	0.045 677	0.082 713
IL-1-b	BMI-C2	81	- 0.001 07	0.003 228	0.739 467	0.818 914
IL-1-b	BMI-C3	8	- 0.008 9	0.010 163	0.381 303	0.673 653

IL-1-RA	BFP-all	382	0.002103	0.001424	0.139654	0.203409
IL-1-RA	BFP-C1	7	-0.00117	0.012313	0.924515	0.999247
IL-1-RA	BFP-C2	99	0.004844	0.002871	0.091517	0.145992
IL-1-RA	BFP-C3	14	0.00745	0.007299	0.307419	0.530584
IL-1-RA	BFP-C4	12	0.003096	0.00973	0.750317	0.935741
IL-1-RA	BFP-C5	9	-0.01365	0.008348	0.102123	0.17772
IL-1-RA	BMI-all	539	0.001021	0.001151	0.375217	0.474331
IL-1-RA	BMI-C1	39	0.002992	0.004184	0.474617	0.57817
IL-1-RA	BMI-C2	81	-0.00283	0.003078	0.357615	0.465247
IL-1-RA	BMI-C3	8	0.006623	0.010849	0.541522	0.763831
IL-10	BFP-all	383	0.001099	0.001021	0.281712	0.345216
IL-10	BFP-C1	7	0.000326	0.008349	0.968876	0.999247
IL-10	BFP-C2	99	0.003252	0.002072	0.116585	0.179568
IL-10	BFP-C3	14	0.002969	0.004574	0.516321	0.708522
IL-10	BFP-C4	13	-0.00244	0.006716	0.716299	0.935741
IL-10	BFP-C5	9	-0.01501	0.007647	0.049622	0.1127
IL-10	BMI-all	539	0.000918	0.00085	0.280039	0.36082
IL-10	BMI-C1	39	0.002241	0.003188	0.482049	0.581933
IL-10	BMI-C2	81	0.000701	0.002306	0.761124	0.835989
IL-10	BMI-C3	8	0.009331	0.006682	0.162576	0.435703
IL-12-p70	BFP-all	383	0.001455	0.000959	0.129481	0.192782
IL-12-p70	BFP-C1	7	-0.00093	0.006736	0.889746	0.999247
IL-12-p70	BFP-C2	99	0.004	0.001	0.037	0.063

			084	96	174	863
IL-12-p70	BFP-C3	14	0.004563	0.004422	0.302126	0.530584
IL-12-p70	BFP-C4	13	0.003562	0.005846	0.542337	0.890073
IL-12-p70	BFP-C5	9	-0.01532	0.005547	0.005749	0.023344
IL-12-p70	BMI-all	539	-1.4E-05	0.000775	0.98522	0.98522
IL-12-p70	BMI-C1	39	-0.00041	0.002979	0.891776	0.922998
IL-12-p70	BMI-C2	81	-0.00331	0.002169	0.127058	0.202687
IL-12-p70	BMI-C3	8	0.008547	0.007181	0.233966	0.513957
IL-13	BFP-all	382	0.001187	0.001407	0.399066	0.469078
IL-13	BFP-C1	7	0.002088	0.013041	0.872795	0.999247
IL-13	BFP-C2	99	0.006018	0.00287	0.036004	0.062657
IL-13	BFP-C3	14	0.005561	0.006687	0.405578	0.624683
IL-13	BFP-C4	12	-0.00427	0.008842	0.62875	0.899436
IL-13	BFP-C5	9	-0.02124	0.009892	0.031788	0.085191
IL-13	BMI-all	538	0.000749	0.001166	0.520622	0.614024
IL-13	BMI-C1	39	0.007072	0.004572	0.121959	0.185711
IL-13	BMI-C2	81	-0.00178	0.003119	0.568965	0.68573
IL-13	BMI-C3	8	-0.00201	0.0105	0.848256	0.924116
IL-16	BFP-all	382	0.000772	0.001471	0.59967	0.65588
IL-16	BFP-C1	7	0.001766	0.009815	0.85721	0.998836
IL-16	BFP-C2	99	0.000174	0.002865	0.951593	0.958748
IL-16	BFP-C3	14	-0.0119	0.007665	0.120596	0.273897

IL-16	BFP-C4	12	-0.00167	0.007743	0.828911	0.957723
IL-16	BFP-C5	9	0.017143	0.00851	0.043971	0.10337
IL-16	BMI-all	538	0.003165	0.001174	0.007022	0.011762
IL-16	BMI-C1	39	0.001156	0.004266	0.786421	0.829767
IL-16	BMI-C2	81	0.003749	0.003269	0.251426	0.350948
IL-16	BMI-C3	8	-0.01401	0.012174	0.249917	0.523263
IL-17	BFP-all	383	0.000129	0.000965	0.893323	0.906858
IL-17	BFP-C1	7	3.13E-05	0.009392	0.997341	0.999247
IL-17	BFP-C2	99	0.001875	0.001971	0.341483	0.415988
IL-17	BFP-C3	14	0.000517	0.00581	0.929048	0.988035
IL-17	BFP-C4	13	-0.00195	0.005914	0.741215	0.935741
IL-17	BFP-C5	9	-0.01188	0.005723	0.037907	0.095841
IL-17	BMI-all	539	-0.00061	0.000818	0.455756	0.560288
IL-17	BMI-C1	39	0.001249	0.003024	0.679557	0.752568
IL-17	BMI-C2	81	-0.00431	0.002466	0.080707	0.134926
IL-17	BMI-C3	8	-0.00188	0.007274	0.795967	0.881484
IL-18	BFP-all	382	0.000264	0.001376	0.847602	0.867012
IL-18	BFP-C1	7	0.004708	0.00965	0.625621	0.822701
IL-18	BFP-C2	99	-0.00019	0.002747	0.944824	0.958748
IL-18	BFP-C3	14	-0.00623	0.006646	0.348639	0.562864
IL-18	BFP-C4	12	0.008609	0.007599	0.257254	0.621629

IL-18	BFP-C5	9	-0.00886	0.012914	0.492537	0.6234
IL-18	BMI-all	539	0.00247	0.001165	0.034048	0.053052
IL-18	BMI-C1	39	0.001364	0.004186	0.744589	0.804636
IL-18	BMI-C2	81	0.005879	0.003115	0.059112	0.104224
IL-18	BMI-C3	8	-0.00783	0.01114	0.482248	0.73822
IL-2	BFP-all	382	0.002499	0.001455	0.085819	0.132181
IL-2	BFP-C1	7	0.000963	0.012869	0.940376	0.999247
IL-2	BFP-C2	99	0.004059	0.00287	0.157295	0.229104
IL-2	BFP-C3	14	0.007007	0.008026	0.382635	0.602329
IL-2	BFP-C4	12	-0.00048	0.007742	0.950485	0.97048
IL-2	BFP-C5	9	-0.00476	0.009074	0.600236	0.687449
IL-2	BMI-all	539	0.000735	0.001176	0.532076	0.619408
IL-2	BMI-C1	39	0.010433	0.004279	0.014756	0.03042
IL-2	BMI-C2	81	-0.00338	0.003144	0.282844	0.379011
IL-2	BMI-C3	8	-0.01197	0.00988	0.225773	0.513957
IL-4	BFP-all	383	0.000714	0.000927	0.441301	0.509779
IL-4	BFP-C1	7	-0.01172	0.006598	0.075788	0.175096
IL-4	BFP-C2	99	0.001239	0.001845	0.502139	0.570225
IL-4	BFP-C3	14	0.00437	0.004527	0.334385	0.558153
IL-4	BFP-C4	13	-0.00234	0.004953	0.635909	0.899436
IL-4	BFP-C5	9	-0.01001	0.005611	0.074509	0.151276

IL-4	BMI-all	539	-6.6E-05	0.000776	0.931789	0.946074
IL-4	BMI-C1	39	-0.00241	0.002826	0.39412	0.509468
IL-4	BMI-C2	81	-0.0028	0.002139	0.189881	0.282712
IL-4	BMI-C3	8	0.004292	0.006907	0.53438	0.763831
IL-5	BFP-all	381	0.001235	0.001505	0.41189	0.479942
IL-5	BFP-C1	7	-0.01105	0.014196	0.436542	0.704778
IL-5	BFP-C2	99	0.005772	0.003047	0.05814	0.096183
IL-5	BFP-C3	14	0.001146	0.00725	0.874382	0.944897
IL-5	BFP-C4	12	-0.00905	0.010239	0.376665	0.742252
IL-5	BFP-C5	9	-0.01523	0.008676	0.079197	0.151436
IL-5	BMI-all	538	0.001069	0.001223	0.382114	0.478535
IL-5	BMI-C1	39	0.010374	0.004347	0.017004	0.034008
IL-5	BMI-C2	81	0.001781	0.003203	0.578265	0.68573
IL-5	BMI-C3	8	-0.00818	0.010061	0.416301	0.715184
IL-6	BFP-all	383	0.002543	0.000976	0.009214	0.015628
IL-6	BFP-C1	7	0.00059	0.007847	0.940088	0.999247
IL-6	BFP-C2	99	0.002476	0.00184	0.178243	0.247709
IL-6	BFP-C3	14	0.002733	0.005676	0.63014	0.782955
IL-6	BFP-C4	13	0.002976	0.00569	0.601003	0.899436
IL-6	BFP-C5	9	-0.00809	0.00558	0.147054	0.241185
IL-6	BMI-all	539	0.002884	0.000787	0.000247	0.000447
IL-6	BMI-C1	39	0.002611	0.003059	0.393419	0.509468

IL-6	BMI-C2	81	0.001171	0.002154	0.58671	0.689641
IL-6	BMI-C3	8	0.009119	0.007558	0.227616	0.513957
IL-7	BFP-all	382	0.002743	0.001517	0.07067	0.110114
IL-7	BFP-C1	7	0.000156	0.014663	0.991512	0.999247
IL-7	BFP-C2	99	0.007299	0.003205	0.022777	0.04181
IL-7	BFP-C3	14	0.003839	0.008672	0.658	0.80545
IL-7	BFP-C4	12	0.000537	0.009734	0.955995	0.97048
IL-7	BFP-C5	9	-0.00732	0.011026	0.506661	0.63393
IL-7	BMI-all	538	0.001592	0.001211	0.18863	0.255317
IL-7	BMI-C1	39	0.011833	0.004319	0.006153	0.013974
IL-7	BMI-C2	81	-0.00011	0.003182	0.972483	0.987218
IL-7	BMI-C3	8	-0.00844	0.010152	0.405838	0.706264
IL-9	BFP-all	382	0.000592	0.001377	0.66737	0.715421
IL-9	BFP-C1	7	0.002092	0.010928	0.848159	0.998836
IL-9	BFP-C2	99	0.001673	0.002663	0.529874	0.591693
IL-9	BFP-C3	14	-0.00041	0.007591	0.957053	0.992645
IL-9	BFP-C4	12	-0.00477	0.010116	0.636948	0.899436
IL-9	BFP-C5	9	-0.00718	0.011498	0.532107	0.637856
IL-9	BMI-all	539	0.001871	0.001151	0.104162	0.148487
IL-9	BMI-C1	39	0.007157	0.004186	0.08734	0.144489
IL-9	BMI-C2	81	-0.0034	0.003078	0.268893	0.37146
IL-9	BMI-C3	8	0.009689	0.009795	0.322577	0.617505

IL2R-a	BFP-all	381	0.001 579	0.001 367	0.248 263	0.313 842
IL2R-a	BFP-C1	7	- 0.000 63	0.009 956	0.949 797	0.999 247
IL2R-a	BFP-C2	99	0.004	0.002 627	0.127 834	0.192 47
IL2R-a	BFP-C3	14	- 0.003 78	0.006 577	0.565 687	0.742 555
IL2R-a	BFP-C4	12	- 0.003 43	0.007 552	0.649 794	0.899 436
IL2R-a	BFP-C5	9	0.005 02	0.008 3	0.545 326	0.637 856
IL2R-a	BMI-all	539	0.000 542	0.001 146	0.636 469	0.712 76
IL2R-a	BMI-C1	39	0.003 204	0.004 164	0.441 632	0.547 951
IL2R-a	BMI-C2	81	0.008 657	0.003 489	0.013 081	0.026 162
IL2R-a	BMI-C3	8	- 0.020 95	0.016 089	0.192 83	0.478 503
MCSF	BFP-all	381	0.000 504	0.001 666	0.762 207	0.804 219
MCSF	BFP-C1	7	- 0.001 52	0.011 672	0.896 568	0.999 247
MCSF	BFP-C2	99	0.003 518	0.003 203	0.272 017	0.343 871
MCSF	BFP-C3	14	- 0.003 84	0.007 993	0.631 038	0.782 955
MCSF	BFP-C4	12	- 0.007 88	0.011 403	0.489 261	0.841 953
MCSF	BFP-C5	9	0.001 442	0.010 292	0.888 591	0.930 244
MCSF	BMI-all	537	0.001 691	0.001 398	0.226 56	0.300 584
MCSF	BMI-C1	39	0.006 339	0.005 063	0.210 614	0.297 077
MCSF	BMI-C2	80	0.004 828	0.004 049	0.233 15	0.339 587
MCSF	BMI-C3	8	- 0.011 31	0.011 69	0.333 223	0.628 899
MIF	BFP-all	383	0.004 242	0.001 509	0.004 949	0.008 503
MIF	BFP-C1	7	0.012	0.010	0.235	0.432

			531	564	547	375
MIF	BFP-C2	100	0.006046	0.002868	0.035048	0.061795
MIF	BFP-C3	14	0.004023	0.007537	0.593489	0.757405
MIF	BFP-C4	12	0.003999	0.007766	0.60654	0.899436
MIF	BFP-C5	9	-0.01266	0.012699	0.31872	0.44488
MIF	BMI-all	539	0.003173	0.001248	0.010995	0.017967
MIF	BMI-C1	39	0.004504	0.005343	0.39921	0.509468
MIF	BMI-C2	81	-0.00356	0.003515	0.310575	0.408011
MIF	BMI-C3	8	-0.00302	0.010409	0.771413	0.881473
PDGF-BB	BFP-all	383	0.000193	0.000927	0.835439	0.861145
PDGF-BB	BFP-C1	7	-0.00012	0.007623	0.987754	0.999247
PDGF-BB	BFP-C2	99	0.000957	0.001774	0.589421	0.647396
PDGF-BB	BFP-C3	14	-0.00111	0.00442	0.801421	0.917867
PDGF-BB	BFP-C4	13	-0.00152	0.004896	0.756419	0.935741
PDGF-BB	BFP-C5	9	-0.01342	0.006424	0.036635	0.095841
PDGF-BB	BMI-all	539	-0.00031	0.000768	0.691274	0.747597
PDGF-BB	BMI-C1	39	0.00049	0.002797	0.861031	0.901391
PDGF-BB	BMI-C2	81	-0.00157	0.002054	0.445535	0.563223
PDGF-BB	BMI-C3	8	-0.00174	0.006449	0.787611	0.881473
SCF	BFP-all	383	0.002233	0.000914	0.014552	0.024073
SCF	BFP-C1	7	0.005949	0.006433	0.355138	0.610109

SCF	BFP-C2	99	0.000 955	0.001 767	0.589 008	0.647 396
SCF	BFP-C3	14	0.005 048	0.004 404	0.251 717	0.449 735
SCF	BFP-C4	13	- 0.001 44	0.004 886	0.767 669	0.935 741
SCF	BFP-C5	9	- 0.003 87	0.006 353	0.542 48	0.637 856
SCF	BMI-all	539	0.001 375	0.000 767	0.073 047	0.108 759
SCF	BMI-C1	39	0.004 485	0.003 375	0.183 905	0.264 981
SCF	BMI-C2	81	0.002 385	0.002 047	0.244 062	0.347 918
SCF	BMI-C3	8	0.001 758	0.006 582	0.789 379	0.881 473
SCGF-b	BFP-all	383	0.001 691	0.001 385	0.221 985	0.288 796
SCGF-b	BFP-C1	7	0.001 502	0.009 57	0.875 325	0.999 247
SCGF-b	BFP-C2	100	0.002 273	0.002 623	0.386 099	0.461 94
SCGF-b	BFP-C3	14	- 0.005 34	0.007 182	0.457 069	0.660 28
SCGF-b	BFP-C4	12	- 0.004 34	0.007 533	0.564 458	0.899 436
SCGF-b	BFP-C5	9	- 0.006 73	0.008 296	0.417 245	0.548 332
SCGF-b	BMI-all	539	0.000 209	0.001 187	0.860 571	0.893 926
SCGF-b	BMI-C1	39	0.004 213	0.004 179	0.313 436	0.428 252
SCGF-b	BMI-C2	81	0.003 872	0.003 291	0.239 389	0.344 926
SCGF-b	BMI-C3	8	- 0.017 4	0.010 876	0.109 715	0.355 654
TNF-a	BFP-all	382	0.001 861	0.001 438	0.195 553	0.264 688
TNF-a	BFP-C1	7	- 0.007 57	0.010 265	0.460 81	0.718 007
TNF-a	BFP-C2	99	0.004 043	0.002 952	0.170 859	0.243 565
TNF-a	BFP-C3	14	0.011 114	0.008 544	0.193 323	0.375 439

TNF-a	BFP-C4	12	-0.0042	0.007784	0.589956	0.899436
TNF-a	BFP-C5	9	0.000192	0.008563	0.982072	0.982072
TNF-a	BMI-all	539	0.001763	0.00118	0.135243	0.190763
TNF-a	BMI-C1	39	0.006408	0.004291	0.135344	0.203777
TNF-a	BMI-C2	81	0.004455	0.003157	0.158164	0.246442
TNF-a	BMI-C3	8	-0.00655	0.012525	0.60099	0.805326
TNF-b	BFP-all	365	-0.0001	0.002113	0.960997	0.968223
TNF-b	BFP-C1	7	-0.01905	0.015972	0.232991	0.432375
TNF-b	BFP-C2	97	0.009552	0.004082	0.019286	0.035894
TNF-b	BFP-C3	14	-0.01931	0.010112	0.056228	0.163795
TNF-b	BFP-C4	12	-0.01086	0.011679	0.352649	0.738359
TNF-b	BFP-C5	9	-0.00398	0.017288	0.818091	0.876993
TNF-b	BMI-all	523	-0.00041	0.001766	0.817947	0.871334
TNF-b	BMI-C1	39	-0.0022	0.007431	0.767536	0.816268
TNF-b	BMI-C2	75	0.003475	0.004734	0.462975	0.579801
TNF-b	BMI-C3	8	-0.00023	0.014702	0.987635	0.992244
TRAIL	BFP-all	383	0.003766	0.000918	4.07E-05	8.38E-05
TRAIL	BFP-C1	7	0.00247	0.009944	0.803844	0.970406
TRAIL	BFP-C2	99	0.005573	0.00182	0.002192	0.004383
TRAIL	BFP-C3	14	-7.9E-05	0.004427	0.985721	0.992645
TRAIL	BFP-C4	13	0.005	0.004	0.226	0.590

			934	905	398	01
TRAIL	BFP-C5	9	0.002 627	0.007 662	0.731 733	0.803 706
TRAIL	BMI-all	539	0.003 464	0.000 787	1.07E -05	2.05E -05
TRAIL	BMI-C1	39	0.002 194	0.002 831	0.438 286	0.547 951
TRAIL	BMI-C2	81	0.005 674	0.002 056	0.005 785	0.012 709
TRAIL	BMI-C3	8	- 0.002 1	0.006 994	0.763 525	0.881 473
VEGF	BFP-all	384	0.001 335	0.001 038	0.198 437	0.265 906
VEGF	BFP-C1	7	0.003 701	0.011	0.736 574	0.927 81
VEGF	BFP-C2	100	0.003 765	0.002 134	0.077 691	0.126 959
VEGF	BFP-C3	14	0.003 303	0.004 767	0.488 404	0.688 906
VEGF	BFP-C4	13	0.001 514	0.006 406	0.813 148	0.957 473
VEGF	BFP-C5	9	- 0.011 45	0.006 63	0.084 099	0.154 374
VEGF	BMI-all	539	0.000 525	0.000 866	0.544 364	0.619 408
VEGF	BMI-C1	39	0.005 579	0.003 361	0.096 95	0.151 062
VEGF	BMI-C2	81	- 0.001 03	0.002 497	0.681 041	0.766 886
VEGF	BMI-C3	8	0.006 228	0.006 971	0.371 643	0.673 653
Birth weight	BFP-all	388	0.000 393	0.000 276	0.155 215	0.222 292
Birth weight	BFP-C1	7	0.001 181	0.001 73	0.494 901	0.728 755
Birth weight	BFP-C2	101	0.000 688	0.000 494	0.163 308	0.235 304
Birth weight	BFP-C3	14	0.001 853	0.000 848	0.028 888	0.097 315
Birth weight	BFP-C4	13	0.001 182	0.002 227	0.595 634	0.899 436
Birth weight	BFP-C5	9	0.002 877	0.001 987	0.147 591	0.241 185
Birth weight	BMI-all	540	0.000 998	0.000 194	2.81E -07	5.46E -07
Birth weight	BMI-C1	39	0.001 013	0.000 577	0.079 399	0.138 176

Birth weight	BMI-C2	82	0.001564	0.000545	0.004111	0.009181
Birth weight	BMI-C3	8	0.007527	0.002127	0.000402	0.004891
Childhood obesity	BFP-all	387	0.02184	0.001818	2.99E-33	2.51E-32
Childhood obesity	BFP-C1	7	0.069638	0.029746	0.019228	0.06633
Childhood obesity	BFP-C2	100	0.032436	0.004629	2.44E-12	9.61E-12
Childhood obesity	BFP-C3	14	0.02829	0.010836	0.009036	0.039059
Childhood obesity	BFP-C4	13	0.011944	0.00644	0.063667	0.266607
Childhood obesity	BFP-C5	9	0.006011	0.008741	0.491646	0.6234
Childhood obesity	BMI-all	537	0.025206	0.001327	2.15E-80	5.76E-79
Childhood obesity	BMI-C1	39	0.037985	0.005216	3.29E-13	1.1E-11
Childhood obesity	BMI-C2	81	0.042771	0.004962	6.72E-18	1.13E-16
Childhood obesity	BMI-C3	8	0.03015	0.010604	0.004465	0.033242
Childhood BMI	BFP-all	345	0.007891	0.000935	3.18E-17	1.58E-16
Childhood BMI	BFP-C1	7	0.025542	0.013702	0.062313	0.154144
Childhood BMI	BFP-C2	86	0.013878	0.002363	4.28E-09	1.27E-08
Childhood BMI	BFP-C3	14	0.011096	0.006548	0.090147	0.236856
Childhood BMI	BFP-C4	10	0.0022	0.003701	0.552191	0.891488
Childhood BMI	BFP-C5	9	0.001934	0.002942	0.510929	0.63393
Childhood BMI	BMI-all	507	0.010392	0.000655	1.22E-56	9.62E-56
Childhood BMI	BMI-C1	37	0.009668	0.002478	9.53E-05	0.00029
Childhood BMI	BMI-C2	74	0.019034	0.002659	8.15E-13	5.2E-12
Childhood BMI	BMI-C3	8	0.01266	0.004011	0.001598	0.014525
Adult BMI	BFP-all	346	0.013706	0.00048	1.6E-179	2.2E-177
Adult BMI	BFP-C1	7	0.031354	0.008083	0.000105	0.001989
Adult BMI	BFP-C2	86	0.017778	0.001075	1.85E-61	2.48E-59

Adult BMI	BFP-C3	14	0.013307	0.003076	1.52E-05	0.000254
Adult BMI	BFP-C4	10	0.012415	0.002426	3.11E-07	2.09E-05
Adult BMI	BFP-C5	9	0.00855	0.001741	9.11E-07	2.44E-05
Adult BMI	BMI-all	507	0.015281	0.000285	0	0
Adult BMI	BMI-C1	37	0.017872	0.001542	4.82E-31	6.46E-29
Adult BMI	BMI-C2	74	0.020393	0.001201	1.29E-64	1.73E-62
Adult BMI	BMI-C3	8	0.0179	0.003426	1.74E-07	1.17E-05
Adult height	BFP-all	321	0.000838	0.000618	0.174535	0.243621
Adult height	BFP-C1	7	-0.00103	0.003348	0.75871	0.932726
Adult height	BFP-C2	79	0.000869	0.000909	0.339151	0.415988
Adult height	BFP-C3	14	0.007605	0.006407	0.235227	0.425952
Adult height	BFP-C4	10	-0.00262	0.002919	0.369308	0.740406
Adult height	BFP-C5	9	-0.00482	0.005652	0.393404	0.532487
Adult height	BMI-all	478	-0.00068	0.000377	0.070285	0.105822
Adult height	BMI-C1	35	-0.0009	0.001455	0.536352	0.641707
Adult height	BMI-C2	66	-8.2E-06	0.000857	0.992341	0.992341
Adult height	BMI-C3	8	0.010665	0.006045	0.077718	0.285632
Whole body fat-free mass	BFP-all	388	0.004655	0.0004	2.35E-31	1.86E-30
Whole body fat-free mass	BFP-C1	7	0.013729	0.004906	0.005135	0.023728
Whole body fat-free mass	BFP-C2	101	0.007419	0.000822	1.82E-19	1.35E-18
Whole body fat-free mass	BFP-C3	14	0.00467	0.001676	0.005339	0.024809
Whole body fat-free mass	BFP-C4	13	0.000442	0.001426	0.756823	0.935741
Whole body fat-free mass	BFP-C5	9	-0.004	0.002795	0.080543	0.151436

			88			
Whole body fat-free mass	BMI-all	540	0.006905	0.000231	7.6E-197	5.1E-195
Whole body fat-free mass	BMI-C1	39	0.00825	0.000902	5.89E-20	2.63E-18
Whole body fat-free mass	BMI-C2	82	0.009732	0.000915	1.99E-26	5.32E-25
Whole body fat-free mass	BMI-C3	8	0.013206	0.002906	5.51E-06	0.000185
Waist-to-hip ratio (female)	BFP-all	345	-0.00087	0.000566	0.126095	0.18985
Waist-to-hip ratio (female)	BFP-C1	7	0.002255	0.003011	0.45399	0.718007
Waist-to-hip ratio (female)	BFP-C2	86	0.000695	0.000585	0.234767	0.30842
Waist-to-hip ratio (female)	BFP-C3	14	-0.0003	0.002683	0.912049	0.977716
Waist-to-hip ratio (female)	BFP-C4	10	-0.00826	0.006017	0.169625	0.505105
Waist-to-hip ratio (female)	BFP-C5	9	-0.03576	0.009649	0.00021	0.002556
Waist-to-hip ratio (female)	BMI-all	507	-0.00054	0.000285	0.058131	0.089536
Waist-to-hip ratio (female)	BMI-C1	37	0.002302	0.00082	0.004983	0.011511
Waist-to-hip ratio (female)	BMI-C2	74	-0.00015	0.000617	0.809606	0.85423
Waist-to-hip ratio (female)	BMI-C3	8	-0.01532	0.005828	0.008578	0.056852
Waist-to-hip ratio (male)	BFP-all	342	0.00215	0.000368	5E-09	1.24E-08
Waist-to-hip ratio (male)	BFP-C1	7	0.001659	0.002814	0.555517	0.767415
Waist-to-hip ratio (male)	BFP-C2	85	0.00154	0.000687	0.024891	0.045073
Waist-to-hip ratio (male)	BFP-C3	14	0.003173	0.002408	0.187647	0.370847
Waist-to-hip ratio (male)	BFP-C4	10	-0.0004	0.002952	0.893312	0.96189
Waist-to-hip ratio (male)	BFP-C5	9	-0.00218	0.004361	0.61726	0.700956

Waist-to-hip ratio (male)	BMI-all	499	0.000108	0.000246	0.659945	0.730848
Waist-to-hip ratio (male)	BMI-C1	37	0.000777	0.000978	0.427164	0.54
Waist-to-hip ratio (male)	BMI-C2	73	-0.0003	0.000578	0.604568	0.704453
Waist-to-hip ratio (male)	BMI-C3	7	-0.00708	0.003161	0.025026	0.134137
non-high-density lipoprotein cholesterol	BFP-all	388	0.00096	0.000396	0.015192	0.024826
non-high-density lipoprotein cholesterol	BFP-C1	7	-0.0007	0.005906	0.906247	0.999247
non-high-density lipoprotein cholesterol	BFP-C2	101	0.002219	0.000706	0.00168	0.003464
non-high-density lipoprotein cholesterol	BFP-C3	14	0.002146	0.001041	0.039207	0.12218
non-high-density lipoprotein cholesterol	BFP-C4	13	-0.00242	0.002485	0.329248	0.729053
non-high-density lipoprotein cholesterol	BFP-C5	9	-0.00972	0.003089	0.001662	0.008907
non-high-density lipoprotein cholesterol	BMI-all	540	0.001522	0.000241	2.47E-10	5.43E-10
non-high-density lipoprotein cholesterol	BMI-C1	39	0.003053	0.001397	0.028917	0.055355
non-high-density lipoprotein cholesterol	BMI-C2	82	0.001651	0.000629	0.008713	0.018242
non-high-density lipoprotein cholesterol	BMI-C3	8	-0.00234	0.003403	0.491536	0.73822
Low-density lipoprotein (LDL) cholesterol	BFP-all	388	1.28E-05	0.000331	0.96905	0.96905
Low-density lipoprotein (LDL) cholesterol	BFP-C1	7	-0.0039	0.005882	0.507599	0.735245
Low-density lipoprotein (LDL) cholesterol	BFP-C2	101	9.94E-05	0.000714	0.88926	0.916622
Low-density lipoprotein (LDL) cholesterol	BFP-C3	14	0.00094	0.00098	0.337391	0.558153
Low-density lipoprotein (LDL) cholesterol	BFP-C4	13	-0.001	0.002224	0.653033	0.899436
Low-density lipoprotein (LDL) cholesterol	BFP-C5	9	-0.00392	0.00196	0.045287	0.104629
Low-density lipoprotein (LDL) cholesterol	BMI-all	540	0.000283	0.000217	0.193082	0.25873
Low-density lipoprotein (LDL) cholesterol	BMI-C1	39	0.000	0.001	0.589	0.672

			701	299	574	61
Low-density lipoprotein (LDL) cholesterol	BMI-C2	82	-0.00024	0.000652	0.710772	0.793695
Low-density lipoprotein (LDL) cholesterol	BMI-C3	8	-0.00046	0.003348	0.889658	0.933046
High-density lipoprotein (HDL) cholesterol	BFP-all	388	-0.00379	0.000474	1.26E-15	5.27E-15
High-density lipoprotein (HDL) cholesterol	BFP-C1	7	-0.01138	0.002357	1.36E-06	6.09E-05
High-density lipoprotein (HDL) cholesterol	BFP-C2	101	-0.00703	0.000928	3.62E-14	1.62E-13
High-density lipoprotein (HDL) cholesterol	BFP-C3	14	-0.00417	0.001288	0.001216	0.008149
High-density lipoprotein (HDL) cholesterol	BFP-C4	13	0.005575	0.003599	0.12139	0.421274
High-density lipoprotein (HDL) cholesterol	BFP-C5	9	0.018201	0.003538	2.68E-07	1.2E-05
High-density lipoprotein (HDL) cholesterol	BMI-all	540	-0.00467	0.000293	4.17E-57	3.49E-56
High-density lipoprotein (HDL) cholesterol	BMI-C1	39	-0.00851	0.000827	7.78E-25	5.21E-23
High-density lipoprotein (HDL) cholesterol	BMI-C2	82	-0.00689	0.001172	4.1E-09	1.72E-08
High-density lipoprotein (HDL) cholesterol	BMI-C3	8	0.00916	0.003436	0.007679	0.054159
Triglycerides	BFP-all	388	0.002726	0.000456	2.19E-09	5.63E-09
Triglycerides	BFP-C1	7	0.00858	0.001558	3.63E-08	2.43E-06
Triglycerides	BFP-C2	101	0.005693	0.000536	2.64E-26	3.7E-25
Triglycerides	BFP-C3	14	0.003074	0.001048	0.003348	0.01762
Triglycerides	BFP-C4	13	-0.00668	0.002634	0.011255	0.083788
Triglycerides	BFP-C5	9	-0.01856	0.004485	3.51E-05	0.000588
Triglycerides	BMI-all	540	0.003732	0.000251	4.51E-50	2.42E-49
Triglycerides	BMI-C1	39	0.007	0.001	1.05E	2.34E

			224	118	-10	-09
Triglycerides	BMI-C2	82	0.004938	0.000628	3.89E-15	3.07E-14
Triglycerides	BMI-C3	8	-0.00767	0.001627	2.44E-06	0.000109
Total cholesterol	BFP-all	388	-0.00053	0.000371	0.155936	0.222292
Total cholesterol	BFP-C1	7	-0.00475	0.005964	0.425601	0.695494
Total cholesterol	BFP-C2	101	-0.00049	0.000766	0.523372	0.589343
Total cholesterol	BFP-C3	14	6.07E-05	0.001028	0.952905	0.992645
Total cholesterol	BFP-C4	13	-0.0013	0.002147	0.544672	0.890073
Total cholesterol	BFP-C5	9	-0.00314	0.001804	0.081368	0.151436
Total cholesterol	BMI-all	540	-0.00024	0.000245	0.330541	0.421833
Total cholesterol	BMI-C1	39	-9.6E-05	0.001302	0.941498	0.963059
Total cholesterol	BMI-C2	82	-0.00112	0.000845	0.183016	0.275552
Total cholesterol	BMI-C3	8	0.000509	0.003361	0.879551	0.933046
Alkaline phosphatase (ALP)	BFP-all	385	0.000332	4.36E-05	2.85E-14	1E-13
Alkaline phosphatase (ALP)	BFP-C1	7	0.000212	0.000356	0.551801	0.767415
Alkaline phosphatase (ALP)	BFP-C2	101	0.000498	9.95E-05	5.68E-07	1.41E-06
Alkaline phosphatase (ALP)	BFP-C3	14	0.000388	0.00018	0.03114	0.099352
Alkaline phosphatase (ALP)	BFP-C4	13	0.000537	0.000183	0.003374	0.03547
Alkaline phosphatase (ALP)	BFP-C5	9	0.000498	0.000258	0.05372	0.118008
Alkaline phosphatase (ALP)	BMI-all	537	0.000235	3.28E-05	6.85E-13	1.58E-12
Alkaline phosphatase (ALP)	BMI-C1	38	0.000584	0.00016	0.000258	0.00072
Alkaline phosphatase (ALP)	BMI-C2	82	0.000304	6.16E-05	8.07E-07	2.46E-06

Alkaline phosphatase (ALP)	BMI-C3	8	-0.0003	0.000246	0.229718	0.513957
Alanine transaminase (ALT)	BFP-all	379	0.000598	7.12E-05	4.48E-17	2.14E-16
Alanine transaminase (ALT)	BFP-C1	7	0.001449	0.000613	0.018122	0.06633
Alanine transaminase (ALT)	BFP-C2	98	0.00119	0.000106	2.35E-29	3.94E-28
Alanine transaminase (ALT)	BFP-C3	13	0.000739	0.000305	0.015315	0.058633
Alanine transaminase (ALT)	BFP-C4	13	-0.00068	0.000412	0.099082	0.379344
Alanine transaminase (ALT)	BFP-C5	9	-0.00244	0.000746	0.00106	0.006765
Alanine transaminase (ALT)	BMI-all	532	0.000702	4.1E-05	1.47E-65	1.8E-64
Alanine transaminase (ALT)	BMI-C1	38	0.001368	0.000221	5.99E-10	8.92E-09
Alanine transaminase (ALT)	BMI-C2	82	0.000907	9.63E-05	4.33E-21	8.28E-20
Alanine transaminase (ALT)	BMI-C3	8	-0.00152	0.000484	0.001626	0.014525
Gamma-glutamyl transferase (GGT)	BFP-all	385	0.000795	9.19E-05	4.93E-18	2.54E-17
Gamma-glutamyl transferase (GGT)	BFP-C1	7	0.001057	0.000591	0.07344	0.175096
Gamma-glutamyl transferase (GGT)	BFP-C2	101	0.001414	0.000162	2.03E-18	1.23E-17
Gamma-glutamyl transferase (GGT)	BFP-C3	14	0.001307	0.000383	0.000648	0.004568
Gamma-glutamyl transferase (GGT)	BFP-C4	13	-0.00067	0.00036	0.061513	0.266607
Gamma-glutamyl transferase (GGT)	BFP-C5	9	-0.00241	0.000986	0.01458	0.050094
Gamma-glutamyl transferase (GGT)	BMI-all	537	0.000837	6.58E-05	4.7E-37	2.03E-36
Gamma-glutamyl transferase (GGT)	BMI-C1	38	0.001459	0.000283	2.65E-07	1.36E-06
Gamma-glutamyl transferase (GGT)	BMI-C2	82	0.000923	0.000165	2.02E-08	7.3E-08
Gamma-glutamyl transferase (GGT)	BMI-C3	8	-0.00115	0.000595	0.054181	0.226885
HOMA-B (β-cell function)	BFP-all	345	0.001371	0.000246	2.45E-08	5.86E-08

HOMA-B (β-cell function)	BFP-C1	7	0.004005	0.00188	0.03313	0.094455
HOMA-B (β-cell function)	BFP-C2	86	0.00293	0.000448	6.47E-11	2.22E-10
HOMA-B (β-cell function)	BFP-C3	14	0.002973	0.001339	0.02635	0.092918
HOMA-B (β-cell function)	BFP-C4	10	0.000185	0.001545	0.904464	0.96189
HOMA-B (β-cell function)	BFP-C5	9	-0.00414	0.001647	0.012038	0.043598
HOMA-B (β-cell function)	BMI-all	507	0.00147	0.000175	4.39E-17	1.11E-16
HOMA-B (β-cell function)	BMI-C1	37	0.002191	0.000747	0.003351	0.008105
HOMA-B (β-cell function)	BMI-C2	74	0.002006	0.000512	9.07E-05	0.000238
HOMA-B (β-cell function)	BMI-C3	8	-0.00075	0.001399	0.592284	0.805326
HOMA-IR (Insulin resistance)	BFP-all	345	0.002276	0.000315	5.06E-13	1.62E-12
HOMA-IR (Insulin resistance)	BFP-C1	7	0.006917	0.003239	0.032693	0.094455
HOMA-IR (Insulin resistance)	BFP-C2	86	0.004489	0.000552	4.26E-16	2.04E-15
HOMA-IR (Insulin resistance)	BFP-C3	14	0.00343	0.001763	0.051692	0.157426
HOMA-IR (Insulin resistance)	BFP-C4	10	-0.00098	0.002065	0.633759	0.899436
HOMA-IR (Insulin resistance)	BFP-C5	9	-0.00771	0.001695	5.49E-06	0.000123
HOMA-IR (Insulin resistance)	BMI-all	507	0.002833	0.000221	8.67E-38	3.87E-37
HOMA-IR (Insulin resistance)	BMI-C1	37	0.005006	0.000933	8.04E-08	4.69E-07
HOMA-IR (Insulin resistance)	BMI-C2	74	0.003377	0.000576	4.45E-09	1.75E-08
HOMA-IR (Insulin resistance)	BMI-C3	8	-0.00019	0.001694	0.911236	0.946555
Incremental insulin at 30 minutes	BFP-all	345	-0.0015	0.001451	0.30219	0.364807
Incremental insulin at 30 minutes	BFP-C1	7	-0.0297	0.010084	0.003228	0.01602
Incremental insulin at 30 minutes	BFP-C2	86	0.002288	0.002724	0.400765	0.471075

Incremental insulin at 30 minutes	BFP-C3	14	0.004792	0.006461	0.458254	0.66028
Incremental insulin at 30 minutes	BFP-C4	10	-0.00201	0.014924	0.892939	0.96189
Incremental insulin at 30 minutes	BFP-C5	9	-0.01155	0.008365	0.167358	0.25777
Incremental insulin at 30 minutes	BMI-all	507	0.000466	0.001111	0.675035	0.741432
Incremental insulin at 30 minutes	BMI-C1	37	-0.0067	0.003911	0.086901	0.144489
Incremental insulin at 30 minutes	BMI-C2	74	0.005319	0.003169	0.093194	0.152293
Incremental insulin at 30 minutes	BMI-C3	8	0.012416	0.009059	0.170522	0.448037
Insulin at 30 minutes	BFP-all	345	-0.00058	0.001492	0.698809	0.743178
Insulin at 30 minutes	BFP-C1	7	-0.02188	0.010102	0.030296	0.090216
Insulin at 30 minutes	BFP-C2	86	0.002974	0.002856	0.297736	0.372866
Insulin at 30 minutes	BFP-C3	14	0.009	0.006461	0.163655	0.337075
Insulin at 30 minutes	BFP-C4	10	-0.00271	0.016025	0.865925	0.96189
Insulin at 30 minutes	BFP-C5	9	-0.01294	0.008469	0.126531	0.21194
Insulin at 30 minutes	BMI-all	507	0.001365	0.00114	0.231146	0.303236
Insulin at 30 minutes	BMI-C1	37	-0.00633	0.003936	0.108046	0.166416
Insulin at 30 minutes	BMI-C2	74	0.004444	0.003239	0.170049	0.258937
Insulin at 30 minutes	BMI-C3	8	0.020089	0.012805	0.116703	0.359499
Corrected insulin response	BFP-all	344	-0.00036	0.001295	0.780942	0.817548
Corrected insulin response	BFP-C1	7	-0.00712	0.011499	0.535621	0.755508
Corrected insulin response	BFP-C2	85	0.002789	0.002469	0.25866	0.330099
Corrected insulin response	BFP-C3	14	0.008	0.006	0.166	0.337

			36	035	022	075
Corrected insulin response	BFP-C4	10	-0.00753	0.010258	0.462757	0.815913
Corrected insulin response	BFP-C5	9	-0.0028	0.009882	0.776908	0.846388
Corrected insulin response	BMI-all	505	0.000858	0.001057	0.416531	0.516807
Corrected insulin response	BMI-C1	37	-0.00366	0.003655	0.316395	0.428252
Corrected insulin response	BMI-C2	74	-0.00173	0.002966	0.559317	0.681349
Corrected insulin response	BMI-C3	8	0.005866	0.0084	0.48497	0.73822
Insulin sensitivity index	BFP-all	388	-0.00384	0.000518	1.26E-13	4.22E-13
Insulin sensitivity index	BFP-C1	7	-0.01492	0.004825	0.001989	0.011589
Insulin sensitivity index	BFP-C2	101	-0.00761	0.000796	1.18E-21	1.13E-20
Insulin sensitivity index	BFP-C3	14	-0.00578	0.001824	0.001531	0.008921
Insulin sensitivity index	BFP-C4	13	0.003567	0.002714	0.188749	0.547585
Insulin sensitivity index	BFP-C5	9	0.014055	0.004467	0.001652	0.008907
Insulin sensitivity index	BMI-all	540	-0.00429	0.000356	1.59E-33	5.93E-33
Insulin sensitivity index	BMI-C1	39	-0.0091	0.001488	9.42E-10	1.26E-08
Insulin sensitivity index	BMI-C2	82	-0.00452	0.000906	6.06E-07	1.92E-06
Insulin sensitivity index	BMI-C3	8	0.003824	0.003513	0.276464	0.55926
Disposition index	BFP-all	345	0.000836	0.001278	0.512763	0.58229
Disposition index	BFP-C1	7	-0.01065	0.013231	0.420899	0.695494
Disposition index	BFP-C2	86	0.003171	0.002458	0.197089	0.268494

Disposition index	BFP-C3	14	0.004035	0.00611	0.509052	0.708522
Disposition index	BFP-C4	10	-0.00658	0.009532	0.490092	0.841953
Disposition index	BFP-C5	9	0.022694	0.008973	0.01144	0.043598
Disposition index	BMI-all	506	0.000651	0.001071	0.543197	0.619408
Disposition index	BMI-C1	37	-0.00666	0.003879	0.085826	0.144489
Disposition index	BMI-C2	74	-0.00186	0.002889	0.520303	0.639638
Disposition index	BMI-C3	8	0.019576	0.009603	0.041496	0.185349
Fasting proinsulin	BFP-all	388	0.002372	0.000529	7.22E-06	1.54E-05
Fasting proinsulin	BFP-C1	7	0.006155	0.00284	0.030237	0.090216
Fasting proinsulin	BFP-C2	101	0.004831	0.001091	9.43E-06	2.18E-05
Fasting proinsulin	BFP-C3	14	0.003791	0.002434	0.119401	0.273897
Fasting proinsulin	BFP-C4	13	-0.00208	0.004879	0.670128	0.907042
Fasting proinsulin	BFP-C5	9	-0.00339	0.002416	0.160914	0.253675
Fasting proinsulin	BMI-all	540	0.002495	0.000457	4.91E-08	9.97E-08
Fasting proinsulin	BMI-C1	39	0.001193	0.001409	0.397263	0.509468
Fasting proinsulin	BMI-C2	82	0.002322	0.001136	0.041002	0.075264
Fasting proinsulin	BMI-C3	8	-0.00179	0.006667	0.788413	0.881473
Fasting insulin (unadjusted)	BFP-all	363	0.00254	0.00031	2.66E-16	1.19E-15
Fasting insulin (unadjusted)	BFP-C1	7	0.007726	0.002604	0.003005	0.01549
Fasting insulin (unadjusted)	BFP-C2	92	0.004459	0.000509	1.88E-18	1.2E-17
Fasting insulin (unadjusted)	BFP-C3	14	0.004062	0.001591	0.010688	0.043939
Fasting insulin (unadjusted)	BFP-C4	10	-0.00195	0.002445	0.42593	0.781845

Fasting insulin (unadjusted)	BFP-C5	9	-0.007	0.002163	0.001207	0.007349
Fasting insulin (unadjusted)	BMI-all	521	0.002835	0.00019	1.91E-50	1.07E-49
Fasting insulin (unadjusted)	BMI-C1	38	0.004774	0.0008	2.44E-09	2.52E-08
Fasting insulin (unadjusted)	BMI-C2	76	0.003774	0.000474	1.74E-15	1.46E-14
Fasting insulin (unadjusted)	BMI-C3	8	0.000981	0.001557	0.528732	0.763831
Fasting glucose (unadjusted)	BFP-all	364	0.001048	0.000227	3.95E-06	8.83E-06
Fasting glucose (unadjusted)	BFP-C1	7	0.002903	0.001991	0.144821	0.281247
Fasting glucose (unadjusted)	BFP-C2	92	0.002162	0.000376	9.14E-09	2.61E-08
Fasting glucose (unadjusted)	BFP-C3	14	-0.00047	0.001134	0.680416	0.80545
Fasting glucose (unadjusted)	BFP-C4	10	-0.00224	0.002317	0.334236	0.729053
Fasting glucose (unadjusted)	BFP-C5	9	-0.00423	0.000845	5.53E-07	1.85E-05
Fasting glucose (unadjusted)	BMI-all	521	0.001453	0.000153	1.71E-21	4.49E-21
Fasting glucose (unadjusted)	BMI-C1	38	0.002836	0.000508	2.31E-08	1.63E-07
Fasting glucose (unadjusted)	BMI-C2	76	0.001651	0.000396	3.06E-05	8.2E-05
Fasting glucose (unadjusted)	BMI-C3	8	0.001181	0.002318	0.610276	0.809673
HbA1c (unadjusted)	BFP-all	388	0.000263	0.000114	0.021172	0.034182
HbA1c (unadjusted)	BFP-C1	7	0.003006	0.000743	5.17E-05	0.001155
HbA1c (unadjusted)	BFP-C2	101	0.00077	0.000192	6.04E-05	0.000135
HbA1c (unadjusted)	BFP-C3	14	0.000979	0.00045	0.029776	0.097315
HbA1c (unadjusted)	BFP-C4	13	-0.00212	0.001008	0.035406	0.215652
HbA1c (unadjusted)	BFP-C5	9	-0.0026	0.000647	6.02E-05	0.000897
HbA1c (unadjusted)	BMI-all	540	0.000483	8.96E-05	6.97E-08	1.39E-07
HbA1c (unadjusted)	BMI-C1	39	0.001732	0.000327	1.16E-07	6.2E-07

HbA1c (unadjusted)	BMI-C2	82	0.000547	0.00024	0.022698	0.042838
HbA1c (unadjusted)	BMI-C3	8	-0.00141	0.001187	0.233433	0.513957
Leptin (unadjusted)	BFP-all	345	0.005989	0.000437	9.28E-43	9.56E-42
Leptin (unadjusted)	BFP-C1	7	0.014686	0.003461	2.21E-05	0.000591
Leptin (unadjusted)	BFP-C2	86	0.006956	0.000887	4.29E-15	1.98E-14
Leptin (unadjusted)	BFP-C3	14	0.006665	0.002098	0.00149	0.008921
Leptin (unadjusted)	BFP-C4	10	0.004681	0.003398	0.168334	0.505105
Leptin (unadjusted)	BFP-C5	9	0.010985	0.003652	0.002634	0.012169
Leptin (unadjusted)	BMI-all	507	0.00558	0.000353	3.21E-56	2.39E-55
Leptin (unadjusted)	BMI-C1	37	0.006621	0.001207	4.12E-08	2.63E-07
Leptin (unadjusted)	BMI-C2	74	0.006752	0.001027	4.83E-11	2.23E-10
Leptin (unadjusted)	BMI-C3	8	0.006433	0.002685	0.016595	0.096683
Adiponectin (BMI adjusted)	BFP-all	345	0.001482	0.000385	0.000118	0.00023
Adiponectin (BMI adjusted)	BFP-C1	7	-0.00159	0.002319	0.491717	0.728755
Adiponectin (BMI adjusted)	BFP-C2	87	-6.8E-05	0.000616	0.911794	0.932674
Adiponectin (BMI adjusted)	BFP-C3	14	0.000992	0.00172	0.564224	0.742555
Adiponectin (BMI adjusted)	BFP-C4	10	0.008396	0.003146	0.00762	0.072938
Adiponectin (BMI adjusted)	BFP-C5	9	0.012421	0.00399	0.001854	0.009553
Adiponectin (BMI adjusted)	BMI-all	507	0.000617	0.000252	0.014343	0.02288
Adiponectin (BMI adjusted)	BMI-C1	37	-0.00057	0.000959	0.549731	0.651893
Adiponectin (BMI adjusted)	BMI-C2	74	-4E-05	0.000597	0.946716	0.983411
Adiponectin (BMI adjusted)	BMI-C3	8	0.010077	0.004619	0.029131	0.150136
Sex hormone-binding globulin (female)	BFP-all	387	-0.00456	0.000661	5.61E-12	1.75E-11

Sex hormone-binding globulin (female)	BFP-C1	7	-0.01601	0.00208	1.39E-14	1.86E-12
Sex hormone-binding globulin (female)	BFP-C2	101	-0.00758	0.000579	4.35E-39	1.94E-37
Sex hormone-binding globulin (female)	BFP-C3	14	-0.00712	0.00144	7.63E-07	2.58E-05
Sex hormone-binding globulin (female)	BFP-C4	13	0.004933	0.002465	0.045356	0.243108
Sex hormone-binding globulin (female)	BFP-C5	9	0.018514	0.00323	9.95E-09	6.66E-07
Sex hormone-binding globulin (female)	BMI-all	537	-0.00611	0.000327	5.9E-78	1.13E-76
Sex hormone-binding globulin (female)	BMI-C1	39	-0.009	0.001914	2.54E-06	1.07E-05
Sex hormone-binding globulin (female)	BMI-C2	82	-0.00744	0.000678	5.13E-28	3.44E-26
Sex hormone-binding globulin (female)	BMI-C3	8	0.004236	0.003798	0.264643	0.545571
Sex hormone-binding globulin (male)	BFP-all	387	-0.00239	0.000817	0.003435	0.006137
Sex hormone-binding globulin (male)	BFP-C1	7	-0.01041	0.002206	2.38E-06	7.99E-05
Sex hormone-binding globulin (male)	BFP-C2	101	-0.00524	0.000699	6.45E-14	2.79E-13
Sex hormone-binding globulin (male)	BFP-C3	14	-0.00291	0.001766	0.098911	0.250078
Sex hormone-binding globulin (male)	BFP-C4	13	0.007056	0.001736	4.83E-05	0.001078
Sex hormone-binding globulin (male)	BFP-C5	9	0.013399	0.004072	0.001001	0.006707
Sex hormone-binding globulin (male)	BMI-all	537	-0.00447	0.000399	3.92E-29	1.28E-28
Sex hormone-binding globulin (male)	BMI-C1	39	-0.0072	0.001788	5.68E-05	0.00019
Sex hormone-binding globulin (male)	BMI-C2	82	-0.00483	0.000751	1.31E-10	5.85E-10
Sex hormone-binding globulin (male)	BMI-C3	8	0.002765	0.00348	0.426964	0.722818
C-reactive protein	BFP-all	345	0.005	0.000	6.91E	2.32E

			951	356	-63	-61
C-reactive protein	BFP-C1	7	0.012442	0.003802	0.001065	0.008923
C-reactive protein	BFP-C2	85	0.008163	0.000641	3.96E-37	1.33E-35
C-reactive protein	BFP-C3	14	0.003542	0.001579	0.02487	0.090071
C-reactive protein	BFP-C4	10	0.003898	0.002803	0.164351	0.505105
C-reactive protein	BFP-C5	9	0.004204	0.002974	0.157442	0.251157
C-reactive protein	BMI-all	502	0.005656	0.000299	1.32E-79	2.95E-78
C-reactive protein	BMI-C1	37	0.007939	0.001319	1.77E-09	1.97E-08
C-reactive protein	BMI-C2	73	0.006315	0.000946	2.4E-11	1.29E-10
C-reactive protein	BMI-C3	8	-0.00286	0.003697	0.438973	0.724266
Abdominal SAT - female	BFP-all	388	0.014259	0.00069	7.03E-95	4.71E-93
Abdominal SAT - female	BFP-C1	7	0.027223	0.00899	0.002461	0.013189
Abdominal SAT - female	BFP-C2	101	0.013804	0.001208	2.95E-30	5.64E-29
Abdominal SAT - female	BFP-C3	14	0.024113	0.002817	1.14E-17	1.52E-15
Abdominal SAT - female	BFP-C4	13	0.018546	0.003087	1.88E-09	2.52E-07
Abdominal SAT - female	BFP-C5	9	0.010475	0.0051	0.039967	0.099177
Abdominal SAT - female	BMI-all	540	0.011895	0.000538	3.4E-108	1.5E-106
Abdominal SAT - female	BMI-C1	39	0.012629	0.002275	2.85E-08	1.91E-07
Abdominal SAT - female	BMI-C2	82	0.016188	0.001509	7.41E-27	2.48E-25
Abdominal SAT - female	BMI-C3	8	0.013436	0.004049	0.000906	0.010114
Abdominal SAT - male	BFP-all	388	0.013682	0.000724	1.03E-79	4.6E-78
Abdominal SAT - male	BFP-C1	7	0.02083	0.008967	0.020179	0.06633
Abdominal SAT - male	BFP-C2	101	0.015666	0.001321	2.03E-32	4.54E-31
Abdominal SAT - male	BFP-C3	14	0.019089	0.00423	6.41E-06	0.00015
Abdominal SAT - male	BFP-C4	13	0.0144	0.003368	1.91E-05	0.000639

Abdominal SAT - male	BFP-C5	9	0.017485	0.004858	0.000319	0.003229
Abdominal SAT - male	BMI-all	540	0.011218	0.000551	3.19E-92	1.07E-90
Abdominal SAT - male	BMI-C1	39	0.013937	0.002187	1.85E-10	3.55E-09
Abdominal SAT - male	BMI-C2	82	0.016346	0.001706	9.54E-22	2.13E-20
Abdominal SAT - male	BMI-C3	8	0.007823	0.005759	0.174321	0.449212
VAT - female	BFP-all	388	0.01048	0.000728	6.02E-47	8.07E-46
VAT - female	BFP-C1	7	0.019547	0.006427	0.002354	0.013143
VAT - female	BFP-C2	101	0.010509	0.001204	2.57E-18	1.44E-17
VAT - female	BFP-C3	14	0.015632	0.004093	0.000134	0.001378
VAT - female	BFP-C4	13	0.009505	0.005125	0.063657	0.266607
VAT - female	BFP-C5	9	-0.01227	0.003523	0.000495	0.003903
VAT - female	BMI-all	540	0.008227	0.00054	2.36E-52	1.38E-51
VAT - female	BMI-C1	39	0.009551	0.001921	6.59E-07	3.05E-06
VAT - female	BMI-C2	82	0.010123	0.001479	7.59E-12	4.24E-11
VAT - female	BMI-C3	8	0.003566	0.004775	0.455214	0.726175
VAT - male	BFP-all	388	0.010558	0.000708	3.1E-50	4.61E-49
VAT - male	BFP-C1	7	0.00995	0.006494	0.125479	0.248124
VAT - male	BFP-C2	101	0.01292	0.001235	1.3E-25	1.45E-24
VAT - male	BFP-C3	14	0.014483	0.00341	2.17E-05	0.00029
VAT - male	BFP-C4	13	0.007127	0.004136	0.084914	0.33466
VAT - male	BFP-C5	9	-0.00017	0.004749	0.971459	0.979802
VAT - male	BMI-all	540	0.008397	0.000537	5.11E-55	3.43E-54
VAT - male	BMI-C1	39	0.010366	0.001944	9.71E-08	5.42E-07
VAT - male	BMI-C2	82	0.012697	0.001518	6.01E-17	6.19E-16

VAT - male	BMI-C3	8	0.001 555	0.005 457	0.775 735	0.881 473
VAT-abdominal SAT ratio - female	BFP-all	388	- 0.000 58	0.000 803	0.472 231	0.540 846
VAT-abdominal SAT ratio - female	BFP-C1	7	- 0.001 96	0.004 984	0.694 814	0.903 932
VAT-abdominal SAT ratio - female	BFP-C2	101	- 0.000 38	0.001 198	0.754 057	0.795 619
VAT-abdominal SAT ratio - female	BFP-C3	14	- 0.004 8	0.005 086	0.345 088	0.562 864
VAT-abdominal SAT ratio - female	BFP-C4	13	- 0.007 93	0.007 59	0.296 101	0.696 097
VAT-abdominal SAT ratio - female	BFP-C5	9	- 0.034 93	0.004 965	1.98E -12	2.65E -10
VAT-abdominal SAT ratio - female	BMI-all	540	- 0.001 38	0.000 547	0.011 869	0.019 162
VAT-abdominal SAT ratio - female	BMI-C1	39	- 0.000 77	0.001 709	0.654 373	0.730 717
VAT-abdominal SAT ratio - female	BMI-C2	82	- 0.004 07	0.001 571	0.009 551	0.019 69
VAT-abdominal SAT ratio - female	BMI-C3	8	- 0.013 59	0.007 051	0.053 958	0.226 885
VAT-abdominal SAT ratio - male	BFP-all	388	- 0.003 29	0.000 844	9.85E -05	0.000 197
VAT-abdominal SAT ratio - male	BFP-C1	7	- 0.013 73	0.007 272	0.059 019	0.150 33
VAT-abdominal SAT ratio - male	BFP-C2	101	- 0.003 14	0.001 568	0.045 043	0.075 447
VAT-abdominal SAT ratio - male	BFP-C3	14	- 0.007 28	0.003 969	0.066 682	0.186 155
VAT-abdominal SAT ratio - male	BFP-C4	13	- 0.009 47	0.004 817	0.049 401	0.248 837
VAT-abdominal SAT ratio - male	BFP-C5	9	- 0.028 91	0.009 101	0.001 488	0.008 669
VAT-abdominal SAT ratio - male	BMI-all	540	-	0.000	1.92E	3.78E

			0.003 09	594	-07	-07
VAT-abdominal SAT ratio - male	BMI-C1	39	- 0.003 11	0.002 116	0.141 727	0.211 016
VAT-abdominal SAT ratio - male	BMI-C2	82	- 0.004 62	0.001 845	0.012 209	0.024 789
VAT-abdominal SAT ratio - male	BMI-C3	8	- 0.007 47	0.006 127	0.223 029	0.513 957
Internal fat - female	BFP-all	388	0.010 775	0.000 693	1.62E -54	3.62E -53
Internal fat - female	BFP-C1	7	0.018 376	0.005 443	0.000 736	0.007 621
Internal fat - female	BFP-C2	101	0.010 156	0.001 136	3.89E -19	2.74E -18
Internal fat - female	BFP-C3	14	0.014 918	0.003 775	7.74E -05	0.000 943
Internal fat - female	BFP-C4	13	0.013 647	0.005 372	0.011 069	0.083 788
Internal fat - female	BFP-C5	9	0.001 996	0.010 891	0.854 586	0.904 58
Internal fat - female	BMI-all	540	0.008 419	0.000 524	3.84E -58	3.68E -57
Internal fat - female	BMI-C1	39	0.010 996	0.001 769	5.06E -10	8.47E -09
Internal fat - female	BMI-C2	82	0.011 56	0.001 375	4.2E- 17	4.83E -16
Internal fat - female	BMI-C3	8	0.007 901	0.004 369	0.070 527	0.270 016
Internal fat - male	BFP-all	388	0.009 84	0.000 713	2.85E -43	3.18E -42
Internal fat - male	BFP-C1	7	0.013 158	0.005 938	0.026 713	0.083 244
Internal fat - male	BFP-C2	101	0.011 876	0.001 151	5.81E -25	5.99E -24
Internal fat - male	BFP-C3	14	0.010 023	0.003 6	0.005 369	0.024 809
Internal fat - male	BFP-C4	13	0.010 573	0.005 398	0.050 139	0.248 837
Internal fat - male	BFP-C5	9	0.009 74	0.008 421	0.247 415	0.360 366
Internal fat - male	BMI-all	540	0.007 935	0.000 513	5.62E -54	3.58E -53
Internal fat - male	BMI-C1	39	0.010 237	0.001 771	7.52E -09	6.72E -08
Internal fat - male	BMI-C2	82	0.011 436	0.001 361	4.32E -17	4.83E -16
Internal fat - male	BMI-C3	8	0.007 417	0.004 745	0.118 045	0.359 499

Pancreas PDFF - female	BFP-all	388	0.008423	0.000774	1.32E-27	8.83E-27
Pancreas PDFF - female	BFP-C1	7	0.016655	0.007204	0.02079	0.06633
Pancreas PDFF - female	BFP-C2	101	0.009467	0.001297	2.93E-13	1.23E-12
Pancreas PDFF - female	BFP-C3	14	0.008875	0.003157	0.004937	0.024502
Pancreas PDFF - female	BFP-C4	13	0.010885	0.006878	0.113518	0.411118
Pancreas PDFF - female	BFP-C5	9	-0.0005	0.00684	0.941822	0.970801
Pancreas PDFF - female	BMI-all	540	0.006811	0.000583	1.47E-31	4.93E-31
Pancreas PDFF - female	BMI-C1	39	0.008997	0.002614	0.000577	0.001577
Pancreas PDFF - female	BMI-C2	82	0.008303	0.001665	6.16E-07	1.92E-06
Pancreas PDFF - female	BMI-C3	8	0.003254	0.004538	0.473291	0.73822
Pancreas PDFF - male	BFP-all	388	0.006991	0.000746	7.03E-21	4.1E-20
Pancreas PDFF - male	BFP-C1	7	0.015446	0.005918	0.009053	0.040436
Pancreas PDFF - male	BFP-C2	101	0.007519	0.001538	1.02E-06	2.49E-06
Pancreas PDFF - male	BFP-C3	14	0.005568	0.003219	0.083713	0.224352
Pancreas PDFF - male	BFP-C4	13	0.01267	0.004977	0.010909	0.083788
Pancreas PDFF - male	BFP-C5	9	0.000836	0.004649	0.857326	0.90458
Pancreas PDFF - male	BMI-all	540	0.004413	0.00059	7.68E-14	1.87E-13
Pancreas PDFF - male	BMI-C1	39	0.008126	0.002082	9.52E-05	0.00029
Pancreas PDFF - male	BMI-C2	82	0.005353	0.001467	0.000264	0.000668
Pancreas PDFF - male	BMI-C3	8	0.002085	0.004632	0.652683	0.835407
Liver PDFF - female	BFP-all	388	0.007199	0.000789	7.49E-20	4.02E-19
Liver PDFF - female	BFP-C1	7	0.019814	0.006101	0.001163	0.00917
Liver PDFF - female	BFP-C2	101	0.008196	0.001328	6.79E-10	2.07E-09
Liver PDFF - female	BFP-C3	14	0.01081	0.003101	0.00049	0.004107
Liver PDFF - female	BFP-C4	13	-0.000	0.003999	0.926438	0.965131

			37			
Liver PDFF - female	BFP-C5	9	-0.01818	0.004114	9.89E-06	0.000189
Liver PDFF - female	BMI-all	540	0.006506	0.000549	2.12E-32	7.68E-32
Liver PDFF - female	BMI-C1	39	0.007722	0.002351	0.001023	0.002688
Liver PDFF - female	BMI-C2	82	0.009594	0.001448	3.49E-11	1.67E-10
Liver PDFF - female	BMI-C3	8	0.002039	0.004451	0.646936	0.835407
Liver PDFF - male	BFP-all	388	0.005649	0.000736	1.73E-14	6.25E-14
Liver PDFF - male	BFP-C1	7	0.010948	0.0047	0.019839	0.06633
Liver PDFF - male	BFP-C2	101	0.008959	0.001323	1.27E-11	4.86E-11
Liver PDFF - male	BFP-C3	14	0.010789	0.003143	0.000598	0.004448
Liver PDFF - male	BFP-C4	13	-0.00415	0.003456	0.229916	0.59001
Liver PDFF - male	BFP-C5	9	-0.01299	0.0046	0.004756	0.019914
Liver PDFF - male	BMI-all	540	0.005537	0.000547	4.09E-24	1.17E-23
Liver PDFF - male	BMI-C1	39	0.005102	0.001989	0.010319	0.022303
Liver PDFF - male	BMI-C2	82	0.005467	0.001504	0.000279	0.000691
Liver PDFF - male	BMI-C3	8	0.002855	0.007328	0.696839	0.872677
Paraspinal muscle PDFF - female	BFP-all	388	0.009606	0.000728	9.93E-40	8.87E-39
Paraspinal muscle PDFF - female	BFP-C1	7	0.023263	0.007354	0.00156	0.010344
Paraspinal muscle PDFF - female	BFP-C2	101	0.010482	0.001215	6.13E-18	3.29E-17
Paraspinal muscle PDFF - female	BFP-C3	14	0.009084	0.003322	0.006245	0.027894
Paraspinal muscle PDFF - female	BFP-C4	13	0.012859	0.004295	0.002752	0.033522
Paraspinal muscle PDFF - female	BFP-C5	9	0.007687	0.011217	0.493137	0.6234
Paraspinal muscle PDFF - female	BMI-all	540	0.007484	0.000568	1.11E-39	5.12E-39
Paraspinal muscle PDFF - female	BMI-C1	39	0.010282	0.002603	7.83E-05	0.000256

Paraspinal muscle PDFF - female	BMI-C2	82	0.011	0.001597	5.68E-12	3.31E-11
Paraspinal muscle PDFF - female	BMI-C3	8	0.003383	0.004412	0.443208	0.724266
Paraspinal muscle PDFF - male	BFP-all	388	0.008567	0.000771	1.05E-28	7.38E-28
Paraspinal muscle PDFF - male	BFP-C1	7	0.022941	0.00652	0.000434	0.005284
Paraspinal muscle PDFF - male	BFP-C2	101	0.009405	0.001391	1.37E-11	5.09E-11
Paraspinal muscle PDFF - male	BFP-C3	14	0.01322	0.003548	0.000194	0.001859
Paraspinal muscle PDFF - male	BFP-C4	13	0.021183	0.006263	0.000718	0.01203
Paraspinal muscle PDFF - male	BFP-C5	9	0.010092	0.010802	0.350163	0.48373
Paraspinal muscle PDFF - male	BMI-all	540	0.006726	0.000555	9.46E-34	3.62E-33
Paraspinal muscle PDFF - male	BMI-C1	39	0.010371	0.002167	1.71E-06	7.4E-06
Paraspinal muscle PDFF - male	BMI-C2	82	0.00938	0.001412	3.06E-11	1.52E-10
Paraspinal muscle PDFF - male	BMI-C3	8	0.014739	0.005936	0.013024	0.079326
Thigh IMAT - female	BFP-all	388	0.010643	0.000696	1.02E-52	1.71E-51
Thigh IMAT - female	BFP-C1	7	0.01562	0.004146	0.000165	0.002454
Thigh IMAT - female	BFP-C2	101	0.010241	0.001213	3.05E-17	1.57E-16
Thigh IMAT - female	BFP-C3	14	0.014737	0.002887	3.33E-07	2.23E-05
Thigh IMAT - female	BFP-C4	13	0.017427	0.005957	0.003441	0.03547
Thigh IMAT - female	BFP-C5	9	0.007838	0.010441	0.452845	0.589138
Thigh IMAT - female	BMI-all	540	0.007719	0.000544	1.18E-45	5.86E-45
Thigh IMAT - female	BMI-C1	39	0.010076	0.001855	5.6E-08	3.41E-07
Thigh IMAT - female	BMI-C2	82	0.012129	0.001418	1.17E-17	1.74E-16
Thigh IMAT - female	BMI-C3	8	0.009519	0.004473	0.033313	0.159427
Thigh IMAT - male	BFP-all	388	0.010081	0.00071	8.27E-46	1.01E-44
Thigh IMAT - male	BFP-C1	7	0.012546	0.006755	0.063268	0.154144
Thigh IMAT - male	BFP-C2	101	0.011897	0.001122	2.76E-26	3.7E-25

Thigh IMAT - male	BFP-C3	14	0.010324	0.0035	0.003182	0.01762
Thigh IMAT - male	BFP-C4	13	0.012437	0.005341	0.019874	0.126818
Thigh IMAT - male	BFP-C5	9	0.018618	0.008151	0.022365	0.06515
Thigh IMAT - male	BMI-all	540	0.007982	0.000507	9.65E-56	6.8E-55
Thigh IMAT - male	BMI-C1	39	0.010195	0.001796	1.38E-08	1.09E-07
Thigh IMAT - male	BMI-C2	82	0.011149	0.001323	3.45E-17	4.62E-16
Thigh IMAT - male	BMI-C3	8	0.009876	0.004231	0.019588	0.109364
Thigh SAT - female	BFP-all	388	0.012685	0.000768	2.93E-61	7.86E-60
Thigh SAT - female	BFP-C1	7	0.022961	0.006804	0.000739	0.007621
Thigh SAT - female	BFP-C2	101	0.010481	0.001198	2.2E-18	1.28E-17
Thigh SAT - female	BFP-C3	14	0.014548	0.003231	6.7E-06	0.00015
Thigh SAT - female	BFP-C4	13	0.029256	0.007111	3.88E-05	0.00104
Thigh SAT - female	BFP-C5	9	0.033768	0.009981	0.000716	0.005107
Thigh SAT - female	BMI-all	540	0.010572	0.000569	3.67E-77	6.15E-76
Thigh SAT - female	BMI-C1	39	0.009722	0.002012	1.36E-06	6.06E-06
Thigh SAT - female	BMI-C2	82	0.014741	0.001362	2.74E-27	1.23E-25
Thigh SAT - female	BMI-C3	8	0.028844	0.005171	2.44E-08	3.27E-06
Thigh SAT - male	BFP-all	388	0.011642	0.000761	8.84E-53	1.69E-51
Thigh SAT - male	BFP-C1	7	0.012224	0.007269	0.092662	0.204462
Thigh SAT - male	BFP-C2	101	0.012719	0.001211	8.61E-26	1.05E-24
Thigh SAT - male	BFP-C3	14	0.014055	0.004435	0.001531	0.008921
Thigh SAT - male	BFP-C4	13	0.023773	0.005542	1.79E-05	0.000639
Thigh SAT - male	BFP-C5	9	0.029743	0.008072	0.000229	0.002556
Thigh SAT - male	BMI-all	540	0.009925	0.000545	4.78E-74	7.12E-73
Thigh SAT - male	BMI-C1	39	0.008593	0.001898	5.99E-06	2.4E-05

Thigh SAT - male	BMI-C2	82	0.013 79	0.001 696	4.34E -16	4.15E -15
Thigh SAT - male	BMI-C3	8	0.018 4	0.007 035	0.008 91	0.056 852
Iliopsoas muscle index - female	BFP-all	388	0.001 906	0.000 77	0.013 318	0.022 308
Iliopsoas muscle index - female	BFP-C1	7	0.008 191	0.006 859	0.232 412	0.432 375
Iliopsoas muscle index - female	BFP-C2	101	0.002 349	0.001 435	0.101 761	0.160 423
Iliopsoas muscle index - female	BFP-C3	14	0.003 133	0.003 941	0.426 656	0.635 244
Iliopsoas muscle index - female	BFP-C4	13	- 0.005 56	0.004 879	0.254 053	0.621 629
Iliopsoas muscle index - female	BFP-C5	9	- 0.015 04	0.008 912	0.091 494	0.163 469
Iliopsoas muscle index - female	BMI-all	540	0.005 384	0.000 534	6.94E -24	1.94E -23
Iliopsoas muscle index - female	BMI-C1	39	0.006 299	0.002 207	0.004 324	0.010 166
Iliopsoas muscle index - female	BMI-C2	82	0.005 258	0.001 537	0.000 625	0.001 469
Iliopsoas muscle index - female	BMI-C3	8	0.004 181	0.004 022	0.298 515	0.588 25
Iliopsoas muscle index - male	BFP-all	388	0.002 003	0.000 7	0.004 239	0.007 475
Iliopsoas muscle index - male	BFP-C1	7	0.009 416	0.004 07	0.020 691	0.066 33
Iliopsoas muscle index - male	BFP-C2	101	0.006 344	0.001 194	1.08E -07	2.85E -07
Iliopsoas muscle index - male	BFP-C3	14	- 0.003 97	0.005 644	0.481 894	0.686 955
Iliopsoas muscle index - male	BFP-C4	13	0.001 274	0.004 323	0.768 145	0.935 741
Iliopsoas muscle index - male	BFP-C5	9	- 0.013 66	0.004 701	0.003 667	0.015 849
Iliopsoas muscle index - male	BMI-all	540	0.005 412	0.000 504	6.07E -27	1.89E -26
Iliopsoas muscle index - male	BMI-C1	39	0.006 672	0.001 802	0.000 214	0.000 61
Iliopsoas muscle index - male	BMI-C2	82	0.007 834	0.001 341	5.18E -09	1.98E -08
Iliopsoas muscle index - male	BMI-C3	8	0.001 332	0.003 912	0.733 395	0.881 404
Total muscle index - female	BFP-all	388	0.003 754	0.000 781	1.51E -06	3.43E -06

Total muscle index - female	BFP-C1	7	0.017259	0.007284	0.017808	0.06633
Total muscle index - female	BFP-C2	101	0.004328	0.001434	0.002533	0.004991
Total muscle index - female	BFP-C3	14	0.006583	0.005003	0.188191	0.370847
Total muscle index - female	BFP-C4	13	-0.00419	0.003718	0.259785	0.621629
Total muscle index - female	BFP-C5	9	-0.0194	0.005411	0.000337	0.003229
Total muscle index - female	BMI-all	540	0.008844	0.000539	1.36E-60	1.4E-59
Total muscle index - female	BMI-C1	39	0.009924	0.00264	0.000171	0.000497
Total muscle index - female	BMI-C2	82	0.011889	0.001587	6.78E-14	5.05E-13
Total muscle index - female	BMI-C3	8	0.008166	0.003993	0.040869	0.185349
Total muscle index - male	BFP-all	388	0.003252	0.000722	6.74E-06	1.46E-05
Total muscle index - male	BFP-C1	7	0.014395	0.005941	0.015388	0.062487
Total muscle index - male	BFP-C2	101	0.008122	0.001283	2.45E-10	7.64E-10
Total muscle index - male	BFP-C3	14	0.002236	0.005246	0.669885	0.80545
Total muscle index - male	BFP-C4	13	0.003063	0.003787	0.418592	0.779045
Total muscle index - male	BFP-C5	9	-0.01555	0.005032	0.002002	0.009938
Total muscle index - male	BMI-all	540	0.008537	0.000494	6.25E-67	8.38E-66
Total muscle index - male	BMI-C1	39	0.010689	0.001905	2.02E-08	1.5E-07
Total muscle index - male	BMI-C2	82	0.01087	0.001559	3.12E-12	1.9E-11
Total muscle index - male	BMI-C3	8	0.015028	0.003945	0.000139	0.00233
Kidney volume - female	BFP-all	388	0.00466	0.00079	3.64E-09	9.2E-09
Kidney volume - female	BFP-C1	7	0.012139	0.006441	0.059459	0.15033
Kidney volume - female	BFP-C2	101	0.004887	0.001268	0.000117	0.000256
Kidney volume - female	BFP-C3	14	0.01315	0.004492	0.003419	0.01762
Kidney volume - female	BFP-C4	13	0.005508	0.003063	0.07213	0.29289

Kidney volume - female	BFP-C5	9	-0.02278	0.006436	0.000401	0.003584
Kidney volume - female	BMI-all	540	0.00672	0.000549	1.9E-34	7.7E-34
Kidney volume - female	BMI-C1	39	0.008208	0.002007	4.31E-05	0.000148
Kidney volume - female	BMI-C2	82	0.007976	0.001642	1.18E-06	3.52E-06
Kidney volume - female	BMI-C3	8	0.015793	0.004819	0.001048	0.010798
Kidney volume - male	BFP-all	388	0.004205	0.000725	6.73E-09	1.64E-08
Kidney volume - male	BFP-C1	7	0.009447	0.007949	0.234689	0.432375
Kidney volume - male	BFP-C2	101	0.006786	0.00129	1.45E-07	3.73E-07
Kidney volume - male	BFP-C3	14	0.011885	0.002788	2.02E-05	0.00029
Kidney volume - male	BFP-C4	13	-0.00172	0.00312	0.581303	0.899436
Kidney volume - male	BFP-C5	9	-0.01239	0.006651	0.062523	0.129348
Kidney volume - male	BMI-all	540	0.005739	0.000536	8.94E-27	2.72E-26
Kidney volume - male	BMI-C1	39	0.006468	0.002627	0.013803	0.0289
Kidney volume - male	BMI-C2	82	0.008274	0.001408	4.24E-09	1.72E-08
Kidney volume - male	BMI-C3	8	0.017748	0.00425	2.97E-05	0.000663
Pancreas volume - female	BFP-all	388	0.000337	0.000647	0.602039	0.65588
Pancreas volume - female	BFP-C1	7	0.006265	0.006979	0.369315	0.618602
Pancreas volume - female	BFP-C2	101	-0.00053	0.001139	0.643414	0.695302
Pancreas volume - female	BFP-C3	14	0.005007	0.003084	0.104513	0.259348
Pancreas volume - female	BFP-C4	13	0.000376	0.003063	0.902188	0.96189
Pancreas volume - female	BFP-C5	9	-0.00967	0.007257	0.182867	0.278456
Pancreas volume - female	BMI-all	540	0.000653	0.000547	0.233084	0.303236
Pancreas volume - female	BMI-C1	39	-0.003	0.00207	0.09604	0.151062

			44			
Pancreas volume - female	BMI-C2	82	7.05E-05	0.001303	0.956846	0.986288
Pancreas volume - female	BMI-C3	8	0.002767	0.005694	0.626964	0.823658
Pancreas volume - male	BFP-all	388	-0.00064	0.000687	0.354274	0.423863
Pancreas volume - male	BFP-C1	7	0.004637	0.004399	0.291823	0.521391
Pancreas volume - male	BFP-C2	101	-0.00174	0.001355	0.198365	0.268494
Pancreas volume - male	BFP-C3	14	0.000558	0.00311	0.857664	0.94324
Pancreas volume - male	BFP-C4	13	-0.00599	0.004759	0.208461	0.570076
Pancreas volume - male	BFP-C5	9	-0.00456	0.007475	0.541544	0.637856
Pancreas volume - male	BMI-all	540	0.000741	0.00054	0.169968	0.234801
Pancreas volume - male	BMI-C1	39	-0.00071	0.002303	0.759016	0.813665
Pancreas volume - male	BMI-C2	82	0.002833	0.00153	0.064024	0.110039
Pancreas volume - male	BMI-C3	8	-0.00213	0.005222	0.68378	0.864401
Liver volume - female	BFP-all	388	0.007902	0.000743	2.04E-26	1.3E-25
Liver volume - female	BFP-C1	7	0.02049	0.006116	0.000808	0.00773
Liver volume - female	BFP-C2	101	0.006891	0.001346	3.06E-07	7.74E-07
Liver volume - female	BFP-C3	14	0.015822	0.003201	7.69E-07	2.58E-05
Liver volume - female	BFP-C4	13	0.004865	0.002998	0.104597	0.389333
Liver volume - female	BFP-C5	9	-0.01242	0.007021	0.076903	0.151436
Liver volume - female	BMI-all	540	0.008778	0.000547	5.08E-58	4.54E-57
Liver volume - female	BMI-C1	39	0.010774	0.002409	7.71E-06	2.95E-05
Liver volume - female	BMI-C2	82	0.010551	0.001588	3.03E-11	1.52E-10

Liver volume - female	BMI-C3	8	0.014629	0.003931	0.000198	0.002946
Liver volume - male	BFP-all	388	0.005706	0.000715	1.47E-15	5.81E-15
Liver volume - male	BFP-C1	7	0.01521	0.004724	0.001285	0.009566
Liver volume - male	BFP-C2	101	0.009586	0.001158	1.28E-16	6.33E-16
Liver volume - male	BFP-C3	14	0.009753	0.002736	0.000363	0.003247
Liver volume - male	BFP-C4	13	-0.00035	0.003894	0.929119	0.965131
Liver volume - male	BFP-C5	9	-0.00764	0.007311	0.295796	0.417227
Liver volume - male	BMI-all	540	0.007514	0.000491	9.1E-53	5.54E-52
Liver volume - male	BMI-C1	39	0.008586	0.002004	1.84E-05	6.66E-05
Liver volume - male	BMI-C2	82	0.009238	0.001276	4.41E-13	3.11E-12
Liver volume - male	BMI-C3	8	0.013979	0.004523	0.001996	0.016714
Spleen volume - female	BFP-all	388	0.004371	0.000692	2.74E-10	7.8E-10
Spleen volume - female	BFP-C1	7	0.014806	0.007041	0.035483	0.099057
Spleen volume - female	BFP-C2	101	0.002673	0.0012	0.025993	0.046441
Spleen volume - female	BFP-C3	14	0.0107	0.00275	0.0001	0.001118
Spleen volume - female	BFP-C4	13	0.005194	0.003983	0.192157	0.547585
Spleen volume - female	BFP-C5	9	-0.00797	0.005043	0.113976	0.195804
Spleen volume - female	BMI-all	540	0.005211	0.000591	1.17E-18	3.01E-18
Spleen volume - female	BMI-C1	39	0.006907	0.002221	0.001875	0.004742
Spleen volume - female	BMI-C2	82	0.006387	0.001432	8.23E-06	2.3E-05
Spleen volume - female	BMI-C3	8	0.005082	0.004701	0.27963	0.55926
Spleen volume - male	BFP-all	388	0.003523	0.000651	6.14E-08	1.44E-07
Spleen volume - male	BFP-C1	7	0.010497	0.004214	0.012732	0.053315
Spleen volume - male	BFP-C2	101	0.00559	0.001182	2.27E-06	5.43E-06

Spleen volume - male	BFP-C3	14	0.001999	0.003663	0.585292	0.754127
Spleen volume - male	BFP-C4	13	-0.00025	0.003094	0.936382	0.965194
Spleen volume - male	BFP-C5	9	-0.00894	0.003553	0.01183	0.043598
Spleen volume - male	BMI-all	540	0.003268	0.000593	3.57E-08	7.35E-08
Spleen volume - male	BMI-C1	39	0.006232	0.001922	0.001185	0.003054
Spleen volume - male	BMI-C2	82	0.006791	0.001431	2.08E-06	6.07E-06
Spleen volume - male	BMI-C3	8	0.00204	0.006067	0.736696	0.881404

Table S5: Mendelian Randomization results for disease outcomes. Two-Sample Mendelian Randomization (MR) was used to identify any causal effects of higher adiposity through clusters on genetically predicted disease outcomes. Disease data was downloaded from FinnGen Data Freeze 8, except Data Freeze 7 was used for peripheral artery disease. nsnp – number of variants, BHp – Benjamini-Hochberg corrected p-value for each cluster to account for false discovery rate.

Outcome	Exposure	nsnp	method	MR_estimate	MR_estimate_se	MR_estimate_pval	BHp	egger_intercept	egger_intercept_se	egger_intercept_pval
Aortic aneurysm	BFP-all	384	MR Egger	0.490267678	0.287197385	0.088620582		-0.002249678	0.003838764	0.558193144
			Weighted median	0.336930999	0.131339618	0.010307424				
			Inverse variance weighted	0.329974277	0.087503394	0.000162602	0.000195123			
			Simple mode	0.160740054	0.384644743	0.676260092				
			Weighted mode	0.203519412	0.265998395	0.444674149				
Aortic aneurysm	BFP-C1	7	MR Egger	0.970368446	1.34698608	0.503540597		-0.022225371	0.030803349	0.502905907
			Weighted median	0.361179947	0.383368441	0.346130043				
			Inverse variance weighted	0.087110481	0.539056386	0.871622364	0.8716224			
			Simple mode	0.556647292	0.918702736	0.566776822				
			Weight	0.4190057	0.425736436	0.363029076				

			ed mode	53						
Aortic aneurysm	BFP-C2	98	MR Egger	0.560650807	0.580464544	0.336538269		-0.002524681	0.008119666	0.756524832
			Weighted median	0.469127356	0.213809978	0.028225907				
			Inverse variance weighted	0.388339505	0.171911488	0.023886478	0.0298581			
			Simple mode	0.357533266	0.507316398	0.482652076				
			Weighted mode	0.883018007	0.417783629	0.037117183				
Aortic aneurysm	BFP-C3	14	MR Egger	-1.649786172	1.420179742	0.267953253		0.043154196	0.023902248	0.096136554
			Weighted median	0.851034769	0.377312347	0.024100837				
			Inverse variance weighted	0.862660679	0.283506929	0.002343721	0.02343721			
			Simple mode	0.894383195	0.598385229	0.158876275				
			Weighted mode	0.702221138	0.537904891	0.214363534				
Aortic aneurysm	BFP-C4	13	MR Egger	2.439354426	1.738868162	0.18825377		-0.023365002	0.025489083	0.378975854
			Weight	0.5197094	0.526888168	0.323949021				

			ed median	33						
			Inverse variance weighted	0.900893316	0.451828317	0.046164718	0.1998228			
			Simple mode	0.257940363	0.973656233	0.795567316				
			Weighted mode	0.159593139	0.933756931	0.867138583				
Aortic aneurysm	BFP-C5	9	MR Egger	1.393902692	1.22323307	0.29197237		-0.036255711	0.019320829	0.102691236
			Weighted median	-0.401079863	0.469614458	0.393070638				
			Inverse variance weighted	-0.76493311	0.476657987	0.108541349	0.1713811			
			Simple mode	-0.718589531	0.655493206	0.304866409				
			Weighted mode	-0.403795641	0.55816631	0.490023213				
Aortic aneurysm	BMI-all	537	MR Egger	0.402126231	0.1591424	0.011796762		-0.00152606	0.002565406	0.552187848
			Weighted median	0.305839211	0.092816973	0.000983942				
			Inverse variance	0.31425245	0.059164434	1.08721E-07	1.35901E-07			

			weighted							
			Simple mode	0.228853056	0.307763402	0.45744395				
			Weighted mode	0.340425853	0.17070853	0.046637022				
Aortic aneurysm	BMI-C1	39	MR Egger	-0.007786496	0.39175933	0.984249318		0.007612694	0.008080548	0.352251767
			Weighted median	0.239503774	0.245293056	0.328867022				
			Inverse variance weighted	0.317699408	0.184422224	0.084947456	0.08787668			
			Simple mode	0.336821884	0.509042054	0.512170303				
			Weighted mode	0.283985839	0.256413879	0.275026681				
Aortic aneurysm	BMI-C2	81	MR Egger	0.097138636	0.304816862	0.7508112		0.005823363	0.006396966	0.365416647
			Weighted median	0.484923327	0.169341362	0.004188769				
			Inverse variance weighted	0.349776255	0.125938668	0.005480346	0.007148278			
			Simple mode	0.341678893	0.363323125	0.349829548				
			Weight	0.4706320	0.226407502	0.040850335				

			ed mode	04						
Aortic aneurysm	BMI-C3	8	MR Egger	2.029355199	0.845241287	0.053228245		-0.030983613	0.015652911	0.095100951
			Weighted median	0.172398158	0.522429707	0.741405235				
			Inverse variance weighted	0.530301811	0.389972395	0.173879141	0.4006927			
			Simple mode	-0.027177062	1.004733794	0.979175621				
			Weighted mode	1.635684855	0.621504073	0.033823198				
Osteoarthritis (hip)	BFP-all	384	MR Egger	0.874484955	0.230861992	0.000176411		-0.007536706	0.003086485	0.015065415
			Weighted median	0.395421704	0.085483291	3.73301E-06				
			Inverse variance weighted	0.337642418	0.070900278	1.91479E-06	3.02335E-06			
			Simple mode	0.5911619	0.322919255	0.067925124				
			Weighted mode	0.776107103	0.398715324	0.052323218				
Osteoarthritis (hip)	BFP-C1	7	MR Egger	1.827481653	0.618436568	0.031701456		-0.01489129	0.014165512	0.341279822
			Weight	1.3949789	0.243019714	9.45712E-09				

			ed median	66						
			Inverse variance weighted	1.236670594	0.260323919	2.02907E-06	8.69603E-06			
			Simple mode	1.505684742	0.373493303	0.006869703				
			Weighted mode	1.413179076	0.260443173	0.001623511				
Osteoarthritis (hip)	BFP-C2	98	MR Egger	0.25013925	0.447397538	0.577396536		-0.000468255	0.006256427	0.940494728
			Weighted median	0.144813608	0.150213623	0.33502031				
			Inverse variance weighted	0.218165737	0.132215434	0.098927048	0.1059933			
			Simple mode	-0.288845874	0.370936173	0.438055324				
			Weighted mode	-0.249639923	0.391168356	0.524853524				
Osteoarthritis (hip)	BFP-C3	14	MR Egger	0.905776092	1.479968234	0.55194204		-0.011373328	0.024907369	0.656095499
			Weighted median	0.361292026	0.256180835	0.158450895				
			Inverse variance	0.243586254	0.286289342	0.394858567	0.5896976			

			weighted							
			Simple mode	0.867549058	0.401134065	0.049783574				
			Weighted mode	0.366626022	0.355254029	0.32088534				
Osteoarthritis (hip)	BFP-C4	13	MR Egger	0.446580291	1.361926748	0.749141244		-0.005990065	0.019964775	0.769746837
			Weighted median	0.011891913	0.376977092	0.974834544				
			Inverse variance weighted	0.05222035	0.342877751	0.878950168	0.922879			
			Simple mode	-0.67699856	0.779190903	0.401973687				
			Weighted mode	0.836243711	0.737602782	0.279049162				
Osteoarthritis (hip)	BFP-C5	9	MR Egger	3.036997313	0.969078917	0.016519378		-0.033647318	0.015330459	0.064221924
			Weighted median	1.063380208	0.385105852	0.005757753				
			Inverse variance weighted	1.037001619	0.400801927	0.009672657	0.02753962			
			Simple mode	0.410341512	0.785931517	0.615739128				
			Weight	1.0940597	0.613559958	0.11241158				

			ed mode	35						
Osteoarthritis (hip)	BMI-all	537	MR Egger	0.548528849	0.115919599	2.84956E-06		-0.00340933	0.001868345	0.068590806
			Weighted median	0.442609239	0.058626994	4.36661E-14				
			Inverse variance weighted	0.352167684	0.0431972	3.56306E-16	6.68073E-16			
			Simple mode	0.62779839	0.219474394	0.004395226				
			Weighted mode	0.573821224	0.132317759	1.72846E-05				
Osteoarthritis (hip)	BMI-C1	39	MR Egger	0.691576213	0.268252551	0.014051331		-0.009009299	0.005534169	0.112024401
			Weighted median	0.674479019	0.155439894	1.43026E-05				
			Inverse variance weighted	0.306490943	0.129227493	0.017705683	0.02124682			
			Simple mode	0.003224849	0.403451938	0.993664279				
			Weighted mode	0.716603556	0.177997157	0.000261461				
Osteoarthritis (hip)	BMI-C2	81	MR Egger	0.670195471	0.207725104	0.001825324		-0.006033374	0.00435532	0.169863467
			Weighted	0.455621971	0.115859952	8.40588E-05				

			median							
			Inverse variance weighted	0.408126952	0.086286077	2.24599E-06	5.88377E-06			
			Simple mode	0.43269867	0.266033715	0.107780367				
			Weighted mode	0.407717711	0.207193134	0.052554468				
Osteoarthritis (hip)	BMI-C3	8	MR Egger	0.818636512	0.653848423	0.257154061		0.001516224	0.012124036	0.904561454
			Weighted median	0.948949953	0.31589236	0.002664295				
			Inverse variance weighted	0.89189131	0.26932109	0.000927547	0.01391321			
			Simple mode	1.110189966	0.546290566	0.081643261				
			Weighted mode	0.925445812	0.318105836	0.022685144				
Osteoarthritis (knee)	BFP-all	384	MR Egger	1.027438217	0.175635667	1.05839E-08		-0.004716398	0.002347856	0.045260473
			Weighted median	0.637572758	0.058226619	6.65575E-28				
			Inverse variance weighted	0.691430446	0.053780676	7.90923E-38	2.37277E-36			

			Simple mode	0.402988178	0.210299727	0.056076519				
			Weighted mode	0.451609482	0.18817833	0.016876203				
Osteoarthritis (knee)	BFP-C1	7	MR Egger	2.431207121	0.371963226	0.001254612		-0.022158615	0.008515463	0.048123475
			Weighted median	1.730179343	0.196618598	1.37216E-18				
			Inverse variance weighted	1.551637035	0.217453306	9.64393E-13	1.44659E-11			
			Simple mode	1.486172315	0.462007906	0.018211123				
			Weighted mode	1.810038665	0.211369906	0.000139216				
Osteoarthritis (knee)	BFP-C2	98	MR Egger	1.0915704	0.33918354	0.001759557		-0.005842669	0.004743023	0.221015869
			Weighted median	0.771879812	0.121341706	2.00173E-10				
			Inverse variance weighted	0.692594334	0.100985642	6.9663E-12	2.98556E-11			
			Simple mode	1.038178051	0.32622844	0.001963032				
			Weighted mode	0.923945086	0.244844297	0.000277051				
Osteoarthritis (knee)	BFP-C3	14	MR	-	1.779636893	0.343402332		0.03357655	0.029951052	0.284201145

			Egger	1.755457922						
			Weighted median	0.294624134	0.224660192	0.189715507				
			Inverse variance weighted	0.199418852	0.358860786	0.578416122	0.7230202			
			Simple mode	0.417308529	0.383723676	0.296557962				
			Weighted mode	0.297554092	0.306964899	0.350064251				
Osteoarthritis (knee)	BFP-C4	13	MR Egger	1.22695255	0.737887709	0.124554268		-0.006255113	0.010811427	0.57453744
			Weighted median	0.509995902	0.255406625	0.045846934				
			Inverse variance weighted	0.814889862	0.187521046	1.38901E-05	0.000138901			
			Simple mode	0.403567711	0.435382564	0.372232331				
			Weighted mode	0.390029772	0.503398855	0.453458972				
Osteoarthritis (knee)	BFP-C5	9	MR Egger	-0.156702261	1.090467085	0.889785249		0.016875798	0.01721179	0.359513616
			Weighted median	0.682484489	0.276572196	0.01360017				

			Inverse variance weighted	0.849001753	0.369242371	0.021487484	0.0460446 1			
			Simple mode	0.971901028	0.455180378	0.065262439				
			Weighted mode	0.549900094	0.370139526	0.175674553				
Osteoarthritis (knee)	BMI-all	537	MR Egger	0.723597664	0.096058327	2.12846E-13		-0.001210168	0.001548238	0.434770846
			Weighted median	0.658250377	0.046337618	8.4661E-46				
			Inverse variance weighted	0.653899406	0.035709347	6.67336E-75	1.001E-73			
			Simple mode	0.94501978	0.168645736	3.36206E-08				
			Weighted mode	0.929882324	0.144743802	2.92605E-10				
Osteoarthritis (knee)	BMI-C1	39	MR Egger	0.945113934	0.214276844	8.55876E-05		-0.003982353	0.004418763	0.373291552
			Weighted median	0.771662784	0.116505909	3.51079E-11				
			Inverse variance weighted	0.774821845	0.100799801	1.50932E-14	2.26397E-13			
			Simple	0.5133682	0.244258156	0.042259579				

			mode	87						
			Weighted mode	0.918656488	0.122318481	5.06894E-09				
Osteoarthritis (knee)	BMI-C2	81	MR Egger	0.63165415	0.18711403	0.001143876		-0.000759839	0.003924868	0.846989137
			Weighted median	0.485574984	0.088811076	4.56415E-08				
			Inverse variance weighted	0.598669152	0.076877469	6.84476E-15	6.84476E-14			
			Simple mode	0.298060764	0.211353986	0.162343889				
			Weighted mode	0.474704668	0.157261698	0.003406464				
Osteoarthritis (knee)	BMI-C3	8	MR Egger	1.395998508	0.660964611	0.079146875		-0.010586812	0.012240144	0.420310885
			Weighted median	0.949698662	0.231800953	4.1846E-05				
			Inverse variance weighted	0.883786823	0.288216213	0.002166517	0.02166517			
			Simple mode	0.688985188	0.342416462	0.084101557				
			Weighted mode	0.980410947	0.248498599	0.005565143				
Asthma	BFP-all	384	MR Egger	0.214124185	0.167156549	0.200977841		0.002854405	0.002234308	0.202189511

			Weighted median	0.429050184	0.05965308	6.36515E-13				
			Inverse variance weighted	0.4175046 4	0.051006969	2.71746E-16	1.16462E-15			
			Simple mode	0.434833486	0.195613837	0.026804484				
			Weighted mode	0.586013978	0.138033622	2.74075E-05				
Asthma	BFP-C1	7	MR Egger	1.141577142	0.489194967	0.066912195		-0.012840031	0.011195158	0.303314593
			Weighted median	0.690721341	0.179980454	0.000124163				
			Inverse variance weighted	0.631753674	0.209556056	0.002572132	0.005511711			
			Simple mode	0.643280455	0.269739199	0.054408939				
			Weighted mode	0.686131723	0.181084858	0.009083949				
Asthma	BFP-C2	98	MR Egger	0.467838808	0.340298451	0.172397053		0.000893564	0.004758647	0.851447842
			Weighted median	0.472719198	0.111435544	2.21451E-05				
			Inverse variance	0.528856159	0.100556487	1.44609E-07	3.33714E-07			

			weighted							
			Simple mode	0.477076927	0.237432839	0.047282378				
			Weighted mode	0.366229103	0.218426379	0.096826995				
Asthma	BFP-C3	14	MR Egger	-0.8774036	1.000724423	0.397825132		0.01591065	0.016843641	0.363490308
			Weighted median	0.019033355	0.210595087	0.927986114				
			Inverse variance weighted	0.048855407	0.198979189	0.806045899	0.8636206			
			Simple mode	0.27257906	0.380799416	0.486769429				
			Weighted mode	0.224911513	0.347262265	0.528470919				
Asthma	BFP-C4	13	MR Egger	-0.073230761	1.090889707	0.947683314		0.000709783	0.01598069	0.965369601
			Weighted median	0.155637494	0.275057919	0.571505715				
			Inverse variance weighted	-0.026468093	0.273408439	0.922878961	0.922879			
			Simple mode	0.747594677	0.560297261	0.206879097				
			Weight	0.8103945	0.639656783	0.229220621				

			ed mode	56						
Asthma	BFP-C5	9	MR Egger	-0.224379134	0.918646641	0.814042037		0.007022084	0.014496817	0.642898524
			Weighted median	0.169150923	0.24898459	0.496907876				
			Inverse variance weighted	0.194155903	0.296680009	0.512835669	0.6689161			
			Simple mode	0.354662021	0.436633793	0.440138965				
			Weighted mode	0.224125375	0.290793347	0.463014865				
Asthma	BMI-all	537	MR Egger	0.191735339	0.091279366	0.036149402		0.002790004	0.001471352	0.058469731
			Weighted median	0.270620354	0.045602294	2.94997E-09				
			Inverse variance weighted	0.352408068	0.034026115	3.89019E-25	1.45882E-24			
			Simple mode	0.318712071	0.141842723	0.025049982				
			Weighted mode	0.22269235	0.077856764	0.004397689				
Asthma	BMI-C1	39	MR Egger	0.064121603	0.227811563	0.779919902		0.009077294	0.004697821	0.061014366
			Weight	0.2683677	0.108225012	0.013148545				

			ed median	66						
			Inverse variance weighted	0.45230132	0.111214386	4.76362E-05	0.000139457			
			Simple mode	0.484165533	0.217358153	0.031912395				
			Weighted mode	0.288462483	0.103505222	0.008260012				
Asthma	BMI-C2	81	MR Egger	0.203997237	0.163276753	0.215209557		0.002503309	0.003424435	0.466933485
			Weighted median	0.181086348	0.081068847	0.025500493				
			Inverse variance weighted	0.312690687	0.067264311	3.34056E-06	7.70898E-06			
			Simple mode	0.142552811	0.170702992	0.406152582				
			Weighted mode	0.153495187	0.111678919	0.173145369				
Asthma	BMI-C3	8	MR Egger	-0.117171351	0.535595474	0.834082124		0.005480043	0.009922923	0.60072043
			Weighted median	-0.151414312	0.252443975	0.548643715				
			Inverse variance	0.147831848	0.225798665	0.512656756	0.6686827			

			weighted							
			Simple mode	-0.151379528	0.430312547	0.735347935				
			Weighted mode	-0.1965446	0.290331463	0.520164005				
Atherosclerosis (excl. cerebral, coronary and PAD)	BFP-all	384	MR Egger	0.59666186	0.247124769	0.016229728		-0.00137664	0.003303838	0.67714733
			Weighted median	0.499847799	0.099360471	4.88819E-07				
			Inverse variance weighted	0.498600249	0.075319433	3.59664E-11	8.99159E-11			
			Simple mode	0.438191866	0.293774191	0.136628742				
			Weighted mode	0.578416465	0.196760998	0.003484577				
Atherosclerosis (excl. cerebral, coronary and PAD)	BFP-C1	7	MR Egger	0.12138109	0.574168793	0.840919268		0.013443526	0.013140461	0.353200863
			Weighted median	0.545002416	0.280191656	0.051762246				
			Inverse variance weighted	0.655206921	0.240519877	0.006447152	0.01137733			
			Simple mode	0.901251842	0.462431463	0.099204447				
			Weight	0.5447958	0.295425334	0.114723335				

			ed mode	81						
Atherosclerosis (excl. cerebral, coronary and PAD)	BFP-C2	98	MR Egger	0.520225572	0.40971511	0.207251811		0.00436501	0.005731705	0.448192297
			Weighted median	0.866643196	0.166717236	2.01139E-07				
			Inverse variance weighted	0.818121675	0.121612863	1.72884E-11	5.76282E-11			
			Simple mode	0.854954673	0.395270824	0.033002027				
			Weighted mode	0.854954673	0.351704256	0.016898533				
Atherosclerosis (excl. cerebral, coronary and PAD)	BFP-C3	14	MR Egger	0.110996665	1.322443056	0.934493626		0.01205911	0.022257523	0.597873228
			Weighted median	0.752005555	0.306724697	0.014217205				
			Inverse variance weighted	0.813059281	0.256848555	0.001548059	0.02322089			
			Simple mode	0.959748598	0.525815684	0.091016038				
			Weighted mode	0.829230278	0.416918122	0.068174741				
Atherosclerosis (excl. cerebral, coronary and PAD)	BFP-C4	13	MR Egger	0.79280775	1.039312216	0.461616634		-0.014091445	0.015241428	0.375037624
			Weighted	0.0082785	0.368368669	0.982070323				

			median							
			Inverse varianc e weight ed	- 0.1345614 06	0.270632781	0.619040759	0.784439			
			Simple mode	0.3946852 28	0.63698294	0.547095154				
			Weight ed mode	0.2166256 85	0.515026844	0.681473834				
Atherosclerosis (excl. cerebral, coronary and PAD)	BFP-C5	9	MR Egger	- 0.4154949 4	1.370138235	0.770513305		- 0.012211301	0.021612951	0.58971415
			Weight ed median	- 1.0048130 04	0.387987822	0.009603017				
			Inverse varianc e weight ed	- 1.1437140 31	0.444599344	0.010097862	0.0275396 2			
			Simple mode	- 1.0552631 84	0.545172864	0.088941813				
			Weight ed mode	- 1.0552631 84	0.439019681	0.042929433				
Atherosclerosis (excl. cerebral, coronary and PAD)	BMI-all	537	MR Egger	0.4200682 21	0.130799174	0.001399563		- 0.000228322	0.002108767	0.91382004
			Weight ed median	0.3937572 43	0.068353567	8.38178E-09				
			Inverse varianc e	0.4069238 51	0.048640783	5.96586E-17	1.2784E- 16			

			weighted							
			Simple mode	0.540119252	0.207525258	0.009506209				
			Weighted mode	0.409741308	0.131817614	0.001980896				
Atherosclerosis (excl. cerebral, coronary and PAD)	BMI-C1	39	MR Egger	-0.158334941	0.302907872	0.604287742		0.018084952	0.006246442	0.006322586
			Weighted median	0.216689596	0.178062745	0.223631494				
			Inverse variance weighted	0.61506729	0.15606943	8.11477E-05	0.000187264			
			Simple mode	0.532096646	0.44032983	0.23435792				
			Weighted mode	0.147679636	0.182353879	0.423067835				
Atherosclerosis (excl. cerebral, coronary and PAD)	BMI-C2	81	MR Egger	0.348761371	0.249801609	0.166579282		0.003173369	0.005244873	0.546885112
			Weighted median	0.405161524	0.129051661	0.001692193				
			Inverse variance weighted	0.486335386	0.103027851	2.35351E-06	5.88377E-06			
			Simple mode	0.332766366	0.251444704	0.189466283				
			Weight	0.4620360	0.161175114	0.00529967				

			Weighted mode	33						
Atherosclerosis (excl. cerebral, coronary and PAD)	BMI-C3	8	MR Egger	0.530139024	0.650115874	0.445968194		-0.020005466	0.012062047	0.148279683
			Weighted median	-0.485527235	0.355056267	0.171479409				
			Inverse variance weighted	-0.435773645	0.288926204	0.131489937	0.3944698			
			Simple mode	-0.559399868	0.546137406	0.339788836				
			Weighted mode	0.042832492	0.368057526	0.910624145				
Atrial fibrillation	BFP-all	384	MR Egger	0.615830026	0.202875436	0.002565828		-0.000839774	0.002711797	0.756977456
			Weighted median	0.569312982	0.075822289	5.98007E-14				
			Inverse variance weighted	0.555996943	0.061791433	2.29947E-19	1.37968E-18			
			Simple mode	0.472349661	0.279212726	0.09151283				
			Weighted mode	0.554349912	0.325526455	0.089391803				
Atrial fibrillation	BFP-C1	7	MR Egger	1.968554469	0.516085311	0.012444062		-0.013259799	0.011817956	0.312832528
			Weight	1.4325035	0.231562207	6.16091E-10				

			ed median	88						
			Inverse variance weighted	1.442342175	0.219980081	5.50136E-11	5.50136E-10			
			Simple mode	0.87594156	0.435869478	0.091199753				
			Weighted mode	1.449876171	0.265255781	0.001563825				
Atrial fibrillation	BFP-C2	98	MR Egger	0.548827192	0.368265201	0.13942263		0.002978815	0.005149772	0.564324173
			Weighted median	0.693856435	0.138615546	5.56832E-07				
			Inverse variance weighted	0.752232856	0.10900104	5.15821E-12	2.57911E-11			
			Simple mode	0.692185221	0.350190551	0.050926963				
			Weighted mode	0.623073879	0.332367239	0.063849075				
Atrial fibrillation	BFP-C3	14	MR Egger	-2.105704691	1.331859896	0.139856946		0.044491284	0.022411567	0.070460108
			Weighted median	0.34524251	0.286492248	0.228177228				
			Inverse variance	0.4850968	0.294359581	0.099358102	0.2709766			

			weighted							
			Simple mode	0.512896428	0.532665199	0.353173992				
			Weighted mode	0.446789981	0.548268373	0.429800009				
Atrial fibrillation	BFP-C4	13	MR Egger	-0.048891668	1.454573718	0.973788425		0.001490631	0.021326603	0.945531412
			Weighted median	0.140283789	0.325826053	0.666796983				
			Inverse variance weighted	0.049223361	0.365029569	0.892732371	0.922879			
			Simple mode	-0.048888744	0.698386479	0.945344782				
			Weighted mode	0.230422014	0.476357589	0.637293892				
Atrial fibrillation	BFP-C5	9	MR Egger	0.763655091	0.850970603	0.399320478		-0.013341733	0.013438706	0.353885319
			Weighted median	0.065973636	0.302739382	0.827489721				
			Inverse variance weighted	-0.030910406	0.28888663	0.914790254	0.9289417			
			Simple mode	-0.6414383	0.533449885	0.263574441				

				93						
			Weighted mode	0.230356955	0.364487407	0.545018646				
Atrial fibrillation	BMI-all	537	MR Egger	0.519740909	0.118727521	1.44365E-05		5.30866E-05	0.001913887	0.97788179
			Weighted median	0.558307031	0.058780029	2.1346E-21				
			Inverse variance weighted	0.522797836	0.044120328	2.16829E-32	1.08415E-31			
			Simple mode	0.456338644	0.195787378	0.020134609				
			Weighted mode	0.545592442	0.115916681	3.20813E-06				
Atrial fibrillation	BMI-C1	39	MR Egger	0.774300292	0.299922777	0.013928657		-0.000544992	0.006189704	0.930313176
			Weighted median	0.616794787	0.150212667	4.02319E-05				
			Inverse variance weighted	0.751015621	0.139625455	7.49892E-08	4.49936E-07			
			Simple mode	0.664080636	0.328046573	0.050004897				
			Weighted mode	0.647571617	0.160476918	0.000254277				
Atrial fibrillation	BMI-C2	81	MR Egger	0.335353154	0.225168776	0.140379074		0.006307349	0.004722268	0.185495542

			Weighted median	0.649257663	0.12083371	7.73729E-08				
			Inverse variance weighted	0.609235846	0.093480551	7.16094E-11	3.58047E-10			
			Simple mode	0.669893255	0.302967301	0.029883038				
			Weighted mode	0.761777085	0.208516564	0.000460886				
Atrial fibrillation	BMI-C3	8	MR Egger	0.385527807	0.833196482	0.659878426		-0.000291849	0.015455836	0.985546903
			Weighted median	0.470503117	0.312439798	0.132092825				
			Inverse variance weighted	0.371434235	0.342869225	0.278670103	0.4817201			
			Simple mode	0.901601157	0.508533538	0.119521272				
			Weighted mode	0.482458275	0.309384282	0.162862105				
Chronic kidney disease	BFP-all	384	MR Egger	0.35344034	0.271309418	0.19345498		0.000459291	0.003627085	0.899301405
			Weighted median	0.366469516	0.117165917	0.001761304				
			Inverse variance	0.386157414	0.082676485	3.00169E-06	4.28813E-06			

			weighted							
			Simple mode	0.273145517	0.415557363	0.511383359				
			Weighted mode	0.352841748	0.320746378	0.271994901				
Chronic kidney disease	BFP-C1	7	MR Egger	0.715228199	0.981358774	0.498805941		0.004852746	0.02245874	0.837468685
			Weighted median	0.902345696	0.335541705	0.007161905				
			Inverse variance weighted	0.907947112	0.375413029	0.015583425	0.02597237			
			Simple mode	0.580835074	0.563424202	0.342339074				
			Weighted mode	0.904517453	0.374576609	0.052233375				
Chronic kidney disease	BFP-C2	98	MR Egger	0.478424881	0.4799585	0.321365245		0.000943221	0.006716219	0.888607148
			Weighted median	0.359539597	0.210414503	0.087502715				
			Inverse variance weighted	0.542774518	0.142142227	0.000134249	0.000201373			
			Simple mode	0.323644858	0.555301373	0.561361982				
			Weighted	0.174581799	0.484103165	0.719160712				

			mode							
Chronic kidney disease	BFP-C3	14	MR Egger	2.751990243	1.847360038	0.162113248		-0.041471233	0.031090704	0.20700739
			Weighted median	0.542325511	0.416163032	0.192521379				
			Inverse variance weighted	0.337447719	0.379725429	0.374184534	0.5896976			
			Simple mode	0.743362275	0.78060406	0.358320673				
			Weighted mode	0.825373113	0.611091287	0.199849153				
Chronic kidney disease	BFP-C4	13	MR Egger	-2.557536888	1.723476293	0.165903748		0.031967298	0.025286332	0.232285094
			Weighted median	-0.091736643	0.511113841	0.857557854				
			Inverse variance weighted	-0.454878666	0.462880282	0.325748503	0.5748503			
			Simple mode	0.009476729	0.876546666	0.991551553				
			Weighted mode	-0.161013402	0.724505666	0.827865268				
Chronic kidney disease	BFP-C5	9	MR Egger	0.213130096	1.082544689	0.849516698		-0.015455598	0.017093186	0.395942347
			Weighted	-0.6017379	0.462290538	0.193037966				

			median	01						
			Inverse variance weighted	-0.707554295	0.367560367	0.054229158	0.0904727			
			Simple mode	-0.528519228	0.685486509	0.462860414				
			Weighted mode	-0.461394117	0.582854812	0.451418395				
Chronic kidney disease	BMI-all	537	MR Egger	0.396824851	0.142658297	0.005599214		0.000231899	0.00230009	0.919729628
			Weighted median	0.319502446	0.090358413	0.000406318				
			Inverse variance weighted	0.41017443	0.053051138	1.06136E-14	1.87299E-14			
			Simple mode	0.15086591	0.278414	0.588129002				
			Weighted mode	0.201579435	0.184911643	0.276141659				
Chronic kidney disease	BMI-C1	39	MR Egger	0.303309454	0.328636382	0.362021705		0.005328155	0.006777652	0.436793939
			Weighted median	0.32693918	0.216111264	0.130323344				
			Inverse variance weight	0.531139345	0.154177638	0.000571095	0.000951825			

			ed							
			Simple mode	0.494633625	0.413576624	0.239110054				
			Weighted mode	0.403572563	0.225931862	0.082038717				
Chronic kidney disease	BMI-C2	81	MR Egger	0.237332176	0.276782243	0.393779633		0.001083473	0.005811398	0.852578248
			Weighted median	0.19354953	0.156322892	0.215664381				
			Inverse variance weighted	0.28430078	0.113946973	0.012594753	0.0151137			
			Simple mode	-0.001803372	0.368346133	0.996105871				
			Weighted mode	0.069978695	0.270788676	0.796742256				
Chronic kidney disease	BMI-C3	8	MR Egger	0.544423173	0.889879821	0.56312266		-0.003474722	0.016536273	0.84052213
			Weighted median	0.150341355	0.451281573	0.739026246				
			Inverse variance weighted	0.376947091	0.367767027	0.305381294	0.4817201			
			Simple mode	0.190241403	0.841094537	0.827521518				
			Weighted	0.15499293	0.530394836	0.778588213				

			mode							
Colorectal cancer	BFP-all	384	MR Egger	0.003627981	0.304331993	0.990494765		0.002280339	0.004068454	0.575471923
			Weighted median	0.251469227	0.143303735	0.079293724				
			Inverse variance weighted	0.166069511	0.092776059	0.07345328	0.07869994			
			Simple mode	0.252430686	0.482609399	0.601239266				
			Weighted mode	0.395067981	0.326653835	0.227240141				
Colorectal cancer	BFP-C1	7	MR Egger	1.009451904	0.853104481	0.289891749		-0.01989994	0.01951113	0.354549123
			Weighted median	0.396212071	0.42203531	0.347826964				
			Inverse variance weighted	0.218711706	0.355966108	0.53893966	0.5774353			
			Simple mode	0.09209085	0.831776728	0.915452292				
			Weighted mode	0.475491351	0.458059618	0.339255976				
Colorectal cancer	BFP-C2	98	MR Egger	-0.381098355	0.579758717	0.512535546		0.009589095	0.008111222	0.24004588
			Weighted	0.320798698	0.241268288	0.183638644				

			median							
			Inverse variance weighted	0.273223048	0.172931062	0.1141175	0.1180526			
			Simple mode	0.31337616	0.652667897	0.632204287				
			Weighted mode	0.384129792	0.531433234	0.471530614				
Colorectal cancer	BFP-C3	14	MR Egger	2.370360581	1.839284618	0.22177981		-0.038969573	0.030951429	0.231951479
			Weighted median	0.246386182	0.49196077	0.616494391				
			Inverse variance weighted	0.101240317	0.375462896	0.787436196	0.8636206			
			Simple mode	0.258608052	0.914442834	0.781779988				
			Weighted mode	0.291189232	0.81317782	0.726024858				
Colorectal cancer	BFP-C4	13	MR Egger	1.709677636	2.552313367	0.516765605		-0.042398375	0.03745062	0.281662125
			Weighted median	-0.97309948	0.599330947	0.104452028				
			Inverse variance weighted	-1.078900301	0.676493564	0.110747588	0.3668496			

			Simple mode	-0.905683919	0.976263111	0.37184458				
			Weighted mode	-0.971777333	0.795197509	0.245148178				
Colorectal cancer	BFP-C5	9	MR Egger	0.69331091	1.374774325	0.629527293		-0.016723468	0.02171381	0.466387327
			Weighted median	-0.196332127	0.623170183	0.752720814				
			Inverse variance weighted	-0.302522146	0.455058575	0.50617954	6.69E-01			
			Simple mode	-0.557750176	1.00115343	0.59269409				
			Weighted mode	0.485483238	0.950671591	0.623369375				
Colorectal cancer	BMI-all	537	MR Egger	-0.053139914	0.17490926	0.76138758		0.002034941	0.002820383	0.470909481
			Weighted median	0.207700218	0.10446412	0.046784648				
			Inverse variance weighted	0.063986878	0.065090498	0.325585375	0.3255854			
			Simple mode	0.256067208	0.305060666	0.401620417				
			Weight	0.2560672	0.173927919	0.141537453				

			ed mode	08						
Colorectal cancer	BMI-C1	39	MR Egger	0.461896965	0.43221166	0.292135754		-0.008623915	0.008914602	0.339629287
			Weighted median	0.294887004	0.26809958	0.271368745				
			Inverse variance weighted	0.093134863	0.20354244	0.647261583	0.6472616			
			Simple mode	0.057039597	0.492243185	0.908360342				
			Weighted mode	0.239900541	0.249199284	0.341791751				
Colorectal cancer	BMI-C2	81	MR Egger	-0.101902876	0.298496691	0.733718665		0.007674203	0.006268823	0.22452051
			Weighted median	0.249342544	0.190775442	0.191214417				
			Inverse variance weighted	0.230659737	0.123697054	0.062221379	0.06666576			
			Simple mode	0.368758709	0.38788298	0.344622226				
			Weighted mode	0.368758709	0.278810941	0.189732988				
Colorectal cancer	BMI-C3	8	MR Egger	-0.390362952	0.985996613	0.705868244		-0.010460961	0.018307231	0.588469649

			Weighted median	-0.924554469	0.564525068	0.101472548				
			Inverse variance weighted	-0.895006217	0.438442166	0.041217795	0.1545667			
			Simple mode	-0.690051196	0.902152613	0.46933141				
			Weighted mode	-1.048777448	0.65216608	0.151838829				
Depression	BFP-all	384	MR Egger	0.058461157	0.152550052	0.701765322		0.000723188	0.002039009	0.723027801
			Weighted median	0.077060928	0.057461093	0.179888799				
			Inverse variance weighted	0.109991618	0.046451885	0.017891245	0.01987916			
			Simple mode	0.052547857	0.192772994	0.785315833				
			Weighted mode	0.070522961	0.175593934	0.688183835				
Depression	BFP-C1	7	MR Egger	0.978752436	0.425348603	0.069680252		-0.020547166	0.009735413	0.088559072
			Weighted median	0.534403307	0.194279394	0.005946903				
			Inverse varianc	0.163029929	0.222940881	0.464614383	0.5162382			

			e weighted							
			Simple mode	-0.29863709	0.458221043	0.538722102				
			Weighted mode	0.489295307	0.196242887	0.046951061				
Depression	BFP-C2	98	MR Egger	-0.085220638	0.32236675	0.792069806		0.003990474	0.004507172	0.37817423
			Weighted median	0.086815376	0.10579324	0.411866376				
			Inverse variance weighted	0.187324341	0.09559089	0.050037137	0.05843822			
			Simple mode	0.094203247	0.249230783	0.706274312				
			Weighted mode	0.001023654	0.194783218	0.995817649				
Depression	BFP-C3	14	MR Egger	-0.55305317	0.671840352	0.42645627		0.008558598	0.011306127	0.46366184
			Weighted median	0.067989685	0.18849253	0.71832202				
			Inverse variance weighted	-0.054723657	0.13417376	0.683378938	0.8200547			

			Simple mode	0.210754466	0.338870457	0.544740033				
			Weighted mode	0.126082039	0.275943301	0.655274936				
Depression	BFP-C4	13	MR Egger	1.444243996	0.903152628	0.138100658		-0.023729837	0.013227959	0.100328929
			Weighted median	0.014801197	0.249525914	0.952699403				
			Inverse variance weighted	-0.119513828	0.257177619	0.642136871	0.784439			
			Simple mode	-0.019304933	0.446769042	0.966244738				
			Weighted mode	0.004009006	0.324272187	0.990339098				
Depression	BFP-C5	9	MR Egger	0.07794472	0.542434592	0.889791135		-0.002011097	0.008557361	0.820924955
			Weighted median	-0.009084989	0.228975413	0.968350877				
			Inverse variance weighted	-0.041964414	0.1797598	0.815414201	0.8736581			
			Simple mode	0.027324969	0.333114391	0.936639026				
			Weighted mode	0.007314046	0.267215925	0.978834066				

Depression	BMI-all	537	MR Egger	0.12044078	0.08710083	0.167310984		-0.000249114	0.00140398	0.859234631
			Weighted median	0.057232676	0.044755351	0.200971166				
			Inverse variance weighted	0.106094466	0.032360975	0.001043717	0.001159685			
			Simple mode	0.12644053	0.143949106	0.380136706				
			Weighted mode	0.054614633	0.104075877	0.599968501				
Depression	BMI-C1	39	MR Egger	0.306528028	0.223675534	0.178815488		-0.00262194	0.004612403	0.573160875
			Weighted median	0.291523006	0.106425634	0.006158553				
			Inverse variance weighted	0.194406549	0.104542874	0.062944007	0.07180398			
			Simple mode	-0.065722697	0.215142956	0.761664867				
			Weighted mode	0.276312713	0.112932605	0.019154176				
Depression	BMI-C2	81	MR Egger	0.220517309	0.153951549	0.155979859		-0.001328852	0.003229461	0.681836333
			Weighted median	0.044391283	0.083545281	0.595180421				

			Inverse variance weighted	0.162829086	0.063274786	0.010071509	0.01258939			
			Simple mode	0.037114448	0.181942583	0.83887943				
			Weighted mode	-0.000930038	0.115336639	0.993586256				
Depression	BMI-C3	8	MR Egger	0.084980822	0.53767616	0.879600967		0.001885994	0.009959478	0.85604894
			Weighted median	0.193232507	0.233406194	0.407738009				
			Inverse variance weighted	0.176204581	0.221756684	0.426854825	0.6097926			
			Simple mode	-0.066231231	0.408502962	0.875783016				
			Weighted mode	0.080360265	0.262218238	0.768166551				
Diabetic nephropathy	BFP-all	384	MR Egger	1.421281956	0.430057464	0.001040063		-0.006690208	0.005750808	0.245413338
			Weighted median	1.070586028	0.173155281	6.29709E-10				
			Inverse variance weighted	0.944886229	0.131425243	6.50129E-13	1.95039E-12			

			Simple mode	0.8728746	0.683896578	0.202613892				
			Weighted mode	1.722631568	0.523584653	0.001094453				
Diabetic nephropathy	BFP-C1	7	MR Egger	1.548376628	1.022783214	0.190475388		0.015447301	0.023432767	0.538901387
			Weighted median	2.042968603	0.532343912	0.000124199				
			Inverse variance weighted	2.160960519	0.427275324	4.24707E-07	2.12354E-06			
			Simple mode	2.154942455	0.923722813	0.058409398				
			Weighted mode	2.060045437	0.565754715	0.010818926				
Diabetic nephropathy	BFP-C2	98	MR Egger	2.155881775	0.697906025	0.002626255		-0.009014968	0.009772458	0.358587165
			Weighted median	1.522802797	0.297456291	3.06482E-07				
			Inverse variance weighted	1.541434213	0.208200569	1.32539E-13	7.95234E-13			
			Simple mode	1.390413923	0.708100219	0.052441532				
			Weighted mode	1.580186725	0.674538542	0.021192149				
Diabetic nephropathy	BFP-C3	14	MR	6.2469290	2.475187635	0.026721045		-	0.041638645	0.041404882

			Egger	39				0.095087723		
			Weighted median	0.515392785	0.632171708	0.414915825				
			Inverse variance weighted	0.708152486	0.568334302	0.212759263	0.4255185			
			Simple mode	-0.267979011	1.184155606	0.824484435				
			Weighted mode	0.172984054	1.390791837	0.902918552				
Diabetic nephropathy	BFP-C4	13	MR Egger	0.097751492	2.099162748	0.96369309		-0.022650497	0.030784414	0.477262251
			Weighted median	-0.997501387	0.728902631	0.171156422				
			Inverse variance weighted	-1.392850954	0.539182764	0.009787065	0.07340299			
			Simple mode	-2.808456757	1.363899062	0.061865012				
			Weighted mode	-0.358162918	1.018646253	0.731229886				
Diabetic nephropathy	BFP-C5	9	MR Egger	0.088617822	1.574343207	0.956684759		-0.052033135	0.02486413	0.074671878
			Weighted median	-2.220189989	0.675188517	0.001008116				

			Inverse variance weighted	-3.009931515	0.535013478	1.84545E-08	1.84545E-07			
			Simple mode	-5.249919978	1.132055018	0.001671614				
			Weighted mode	-2.205879534	0.817004392	0.027075694				
Diabetic nephropathy	BMI-all	537	MR Egger	1.282050525	0.222526277	1.40953E-08		-0.005844494	0.003589707	0.104086938
			Weighted median	0.95134247	0.129976511	2.49191E-13				
			Inverse variance weighted	0.945873169	0.083094267	5.07429E-30	2.1747E-29			
			Simple mode	0.983331862	0.398164226	0.013834428				
			Weighted mode	1.066307851	0.249010883	2.19337E-05				
Diabetic nephropathy	BMI-C1	39	MR Egger	1.061619927	0.6273669	0.099017347		0.010733253	0.012949673	0.412508064
			Weighted median	1.210451643	0.335936408	0.000314303				
			Inverse variance weighted	1.519966528	0.295063187	2.58652E-07	1.10851E-06			

			Simple mode	1.409845895	0.610317474	0.026413675				
			Weighted mode	1.22581092	0.334244953	0.000747152				
Diabetic nephropathy	BMI-C2	81	MR Egger	1.778853539	0.42862226	8.31862E-05		-0.017892893	0.009014923	0.050637226
			Weighted median	0.946494853	0.229455553	3.708E-05				
			Inverse variance weighted	1.004923462	0.181212218	2.93013E-08	1.25577E-07			
			Simple mode	0.976503614	0.533324193	0.070827442				
			Weighted mode	1.037209437	0.359251534	0.004997589				
Diabetic nephropathy	BMI-C3	8	MR Egger	0.260800038	1.201720572	0.835382394		-0.020236881	0.022296992	0.399070811
			Weighted median	-0.536467271	0.693854436	0.439421917				
			Inverse variance weighted	-0.716199498	0.528739844	0.175564023	0.4006927			
			Simple mode	-2.025273772	1.059752468	0.097602104				
			Weighted mode	-0.585395003	0.704104595	0.433186956				

Diabetic neuropathy	BFP-all	384	MR Egger	0.956356489	0.469605008	0.042388097		-0.00200263	0.006280004	0.749984285
			Weighted median	0.748840399	0.216988387	0.000558385				
			Inverse variance weighted	0.813753827	0.143201183	1.32669E-08	2.84291E-08			
			Simple mode	0.401936133	0.723482971	0.578837994				
			Weighted mode	0.680057918	0.568369938	0.232239383				
Diabetic neuropathy	BFP-C1	7	MR Egger	0.140476638	1.276473023	0.916649898		0.043878459	0.029145731	0.192542378
			Weighted median	1.112228286	0.663293746	0.093576486				
			Inverse variance weighted	1.887650431	0.543106303	0.000509606	0.001698688			
			Simple mode	1.355580864	1.128715357	0.275008833				
			Weighted mode	1.193037953	0.705039817	0.141563954				
Diabetic neuropathy	BFP-C2	98	MR Egger	1.946734717	0.926798854	0.038306436		-0.007755405	0.012977611	0.551514718
			Weighted median	1.008422087	0.389891065	0.009697865				
			Inverse	1.4180706	0.275408019	2.61905E-07	5.61226E-			

			variance weighted	59			07			
			Simple mode	0.757736606	0.977111004	0.439938648				
			Weighted mode	0.757736606	0.864594263	0.382973994				
Diabetic neuropathy	BFP-C3	14	MR Egger	1.998118016	3.022260291	0.521018275		-0.023035189	0.050876271	0.658792244
			Weighted median	0.748849581	0.743962225	0.314141765				
			Inverse variance weighted	0.657284279	0.584676022	0.260934359	0.4892519			
			Simple mode	0.850733988	1.425509227	0.560895675				
			Weighted mode	0.464184426	1.161568862	0.695925618				
Diabetic neuropathy	BFP-C4	13	MR Egger	0.711938179	2.343184875	0.766924719		-0.021373044	0.034376788	0.546789533
			Weighted median	-0.542128977	0.853500786	0.525309232				
			Inverse variance weighted	-0.693973323	0.614058527	0.258416401	0.5168328			
			Simple mode	-0.3515941	1.60198347	0.829969561				

				01						
			Weighted mode	0.01652942	1.455798636	0.991127442				
Diabetic neuropathy	BFP-C5	9	MR Egger	-1.10071324	1.962291784	0.592337192		-0.017783162	0.030956068	0.583634633
			Weighted median	-1.601146292	0.865391019	0.064284757				
			Inverse variance weighted	-2.161064922	0.666016824	0.001175495	0.005877477			
			Simple mode	-1.440660202	1.345146352	0.315411016				
			Weighted mode	-1.306849415	1.056567155	0.251206611				
Diabetic neuropathy	BMI-all	537	MR Egger	1.279855	0.261268964	1.28073E-06		-0.006888955	0.004216143	0.102858207
			Weighted median	0.90537622	0.151527151	2.30076E-09				
			Inverse variance weighted	0.883784262	0.09763578	1.40569E-19	3.83369E-19			
			Simple mode	0.875033539	0.537032997	0.103819051				
			Weighted mode	0.777766153	0.34116505	0.023015414				

Diabetic neuropathy	BMI-C1	39	MR Egger	0.511335083	0.696633135	0.467569321		0.019139007	0.014369141	0.191022941
			Weighted median	0.73313008	0.379790263	0.053562851				
			Inverse variance weighted	1.32976793	0.331556658	6.05439E-05	0.00015136			
			Simple mode	0.870753972	0.732256573	0.241763212				
			Weighted mode	0.605969665	0.425042999	0.162127928				
Diabetic neuropathy	BMI-C2	81	MR Egger	1.736728798	0.480944335	0.00053372		-0.019780415	0.010126132	0.05431226
			Weighted median	0.948343009	0.305094281	0.001881284				
			Inverse variance weighted	0.882533735	0.203714786	1.47625E-05	2.9525E-05			
			Simple mode	1.052555269	0.719323785	0.147315217				
			Weighted mode	1.236234244	0.505717854	0.016703079				
Diabetic neuropathy	BMI-C3	8	MR Egger	1.437001939	1.797990619	0.45462571		-0.033530985	0.0334452	0.354774449
			Weighted median	0.974096791	0.895345451	0.276614367				
			Inverse	-	0.800327367	0.824657483	0.9425136			

			variance weighted	0.177319443						
			Simple mode	0.319794397	1.651145935	0.851926561				
			Weighted mode	0.895024034	1.000552533	0.400748528				
Diabetic retinopathy	BFP-all	384	MR Egger	0.935701322	0.310617228	0.002764538		-0.003946145	0.004153008	0.342616659
			Weighted median	0.774792113	0.121028822	1.53641E-10				
			Inverse variance weighted	0.654627337	0.094753555	4.88989E-12	1.33361E-11			
			Simple mode	0.896463185	0.444722661	0.044520849				
			Weighted mode	1.007745281	0.408729119	0.014117592				
Diabetic retinopathy	BFP-C1	7	MR Egger	1.451387153	1.202074446	0.281271845		0.013158581	0.027531907	0.652857423
			Weighted median	1.916162759	0.336615238	1.25235E-08				
			Inverse variance weighted	1.973566569	0.467944504	2.47001E-05	9.26254E-05			
			Simple mode	1.685518179	0.669637705	0.045467337				

			Weighted mode	1.880448476	0.370566284	0.002277795				
Diabetic retinopathy	BFP-C2	98	MR Egger	1.626214081	0.508095555	0.001859589		-0.004813041	0.007111098	0.500138182
			Weighted median	1.19347768	0.205596409	6.43837E-09				
			Inverse variance weighted	1.297900534	0.150794116	7.49299E-18	5.61975E-17			
			Simple mode	1.27947645	0.524061045	0.016440089				
			Weighted mode	1.078521902	0.460056148	0.021100676				
Diabetic retinopathy	BFP-C3	14	MR Egger	3.483255138	1.504519466	0.039106383		-0.049606871	0.025325043	0.073785872
			Weighted median	0.836829348	0.378618526	0.027090061				
			Inverse variance weighted	0.595553942	0.331618019	0.072509875	0.2175296			
			Simple mode	0.818139944	0.677776392	0.248901237				
			Weighted mode	1.205252925	0.553745699	0.04854144				
Diabetic retinopathy	BFP-C4	13	MR Egger	0.341798011	1.230078326	0.786267783		-0.028281038	0.018031018	0.145070854
			Weight	-	0.414787135	0.005894529				

			ed median	1.142154331						
			Inverse variance weighted	-1.520240511	0.322049327	2.35242E-06	3.52863E-05			
			Simple mode	-1.249627944	0.706824821	0.102462155				
			Weighted mode	-1.052041539	0.516016175	0.06412903				
Diabetic retinopathy	BFP-C5	9	MR Egger	-0.840226522	1.264655093	0.527711079		-0.030681781	0.019905124	0.167120675
			Weighted median	-2.290110522	0.499372644	4.51872E-06				
			Inverse variance weighted	-2.674543658	0.463335725	7.81658E-09	1.17249E-07			
			Simple mode	-2.224700137	0.673132909	0.010778314				
			Weighted mode	-2.131341711	0.48651982	0.0023463				
Diabetic retinopathy	BMI-all	537	MR Egger	0.983056032	0.182414785	1.06313E-07		-0.006098466	0.002942124	0.038668138
			Weighted median	0.68948211	0.07926608	3.36819E-18				
			Inverse	0.6321743	0.068179617	1.82361E-20	5.47084E-			

			variance weighted	99			20			
			Simple mode	0.696769736	0.277267978	0.01226382				
			Weighted mode	0.855218357	0.237447763	0.000345527				
Diabetic retinopathy	BMI-C1	39	MR Egger	0.787765361	0.746649928	0.298235857		0.011434287	0.015410895	0.462794964
			Weighted median	1.030964322	0.226111626	5.12665E-06				
			Inverse variance weighted	1.276141748	0.350368939	0.000270234	0.000506689			
			Simple mode	1.117430241	0.448314106	0.017160557				
			Weighted mode	1.075954794	0.246118977	9.23708E-05				
Diabetic retinopathy	BMI-C2	81	MR Egger	0.881690145	0.286112748	0.002833742		-0.009461269	0.006012459	0.119574076
			Weighted median	0.358955764	0.156288053	0.021632605				
			Inverse variance weighted	0.471983956	0.119718681	8.06562E-05	0.000142158			
			Simple mode	0.384666154	0.360456878	0.289108221				

			Weighted mode	0.246462921	0.301426844	0.41598328			
Diabetic retinopathy	BMI-C3	8	MR Egger	0.225410934	1.166970903	0.853207178		-0.028818978	0.021647289	0.231438582
			Weighted median	-0.775775704	0.440214096	0.07802384				
			Inverse variance weighted	-1.166285118	0.546543189	0.03284848	0.1531711			
			Simple mode	-1.20609725	0.715703886	0.135819967				
			Weighted mode	-0.686962	0.482922687	0.197874705				
Deep vein thrombosis	BFP-all	384	MR Egger	0.902839632	0.275601103	0.001149572		-0.004058163	0.00368434	0.271388765
			Weighted median	0.693949517	0.113761942	1.06059E-09				
			Inverse variance weighted	0.613744391	0.084095974	2.91768E-13	9.72559E-13			
			Simple mode	0.830877124	0.351228912	0.018496866				
			Weighted mode	0.894725038	0.255159239	0.000507801				
Deep vein thrombosis	BFP-C1	7	MR Egger	2.040879878	0.69384694	0.032204471		-0.027382434	0.015889455	0.145440456

			Weighted median	1.272567253	0.341844353	0.000197142				
			Inverse variance weighted	0.9544097	0.289739273	0.000987621	0.002693513			
			Simple mode	1.005497516	0.611657391	0.151303441				
			Weighted mode	1.260784921	0.375470791	0.015269687				
Deep vein thrombosis	BFP-C2	98	MR Egger	0.264812807	0.526020976	0.61581777		0.003913117	0.007355586	0.595960184
			Weighted median	0.564970287	0.211912532	0.007674733				
			Inverse variance weighted	0.532010104	0.155739417	0.000635419	0.00086648			
			Simple mode	0.543688984	0.460520579	0.240649951				
			Weighted mode	0.601306177	0.365665745	0.103327771				
Deep vein thrombosis	BFP-C3	14	MR Egger	0.748542119	1.364954048	0.593472649		-0.002795903	0.022972707	0.905146617
			Weighted median	0.807886688	0.375563549	0.031465799				
			Inverse variance	0.585763462	0.270933233	0.030616523	0.1530826			

			weighted							
			Simple mode	0.946612135	0.644969421	0.165964091				
			Weighted mode	1.02000109	0.618239288	0.12291409				
Deep vein thrombosis	BFP-C4	13	MR Egger	3.473334003	1.963484332	0.104581745		-0.03659339	0.028798455	0.230061359
			Weighted median	1.514269836	0.483537114	0.001738259				
			Inverse variance weighted	1.065423068	0.527085063	0.04324361	0.1998228			
			Simple mode	1.740477064	0.704000219	0.029371234				
			Weighted mode	1.664487059	0.528922965	0.008422709				
Deep vein thrombosis	BFP-C5	9	MR Egger	-0.004790812	1.49422115	0.997531265		0.01588408	0.023573894	0.522058189
			Weighted median	0.817567874	0.506625045	0.106580932				
			Inverse variance weighted	0.942238351	0.489584736	0.054283622	0.0904727			
			Simple mode	1.816267091	1.003753273	0.1079772				
			Weight	0.6588427	0.675750056	0.358125752				

			ed mode	47						
Deep vein thrombosis	BMI-all	537	MR Egger	0.74958724	0.152126243	1.1128E-06		-0.004904759	0.002452129	0.045983135
			Weighted median	0.573422645	0.07955314	5.67607E-13				
			Inverse variance weighted	0.467134132	0.056740272	1.82811E-16	3.65623E-16			
			Simple mode	0.632666893	0.243103084	0.009511796				
			Weighted mode	0.662314864	0.135937031	1.45521E-06				
Deep vein thrombosis	BMI-C1	39	MR Egger	0.575406486	0.369656604	0.128079423		-0.001936349	0.007622219	0.800871109
			Weighted median	0.731240497	0.206158531	0.000389672				
			Inverse variance weighted	0.492590359	0.172118282	0.004210708	0.006015297			
			Simple mode	0.705035389	0.365139524	0.060985568				
			Weighted mode	0.684534888	0.218824134	0.003369271				
Deep vein thrombosis	BMI-C2	81	MR Egger	0.621904517	0.281409405	0.030002121		-0.001976647	0.005903094	0.738626177
			Weighted	0.5814611	0.159174759	0.000259221				

			median							
			Inverse variance weighted	0.536109968	0.115728574	3.61316E-06	7.74249E-06			
			Simple mode	0.69728024	0.348446776	0.048771618				
			Weighted mode	0.69728024	0.210466558	0.001387578				
Deep vein thrombosis	BMI-C3	8	MR Egger	2.1915609	0.833220362	0.039047606		-0.043780691	0.015470631	0.029960812
			Weighted median	0.638419744	0.447660516	0.153832334				
			Inverse variance weighted	0.079302335	0.523877603	0.879679364	0.9425136			
			Simple mode	0.355652746	0.665320879	0.609506325				
			Weighted mode	0.677324191	0.421211575	0.151862213				
Gallstones	BFP-all	384	MR Egger	0.831977203	0.165852749	8.08273E-07		-0.00294241	0.002217711	0.185374619
			Weighted median	0.668148559	0.063267173	4.53046E-26				
			Inverse variance weighted	0.622440196	0.050701069	1.20874E-34	1.81312E-33			

			Simple mode	0.848835606	0.255210292	0.00096607				
			Weighted mode	0.970639289	0.210090657	5.24758E-06				
Gallstones	BFP-C1	7	MR Egger	1.748327656	0.651734278	0.043685072		-0.020505906	0.014918936	0.22769622
			Weighted median	1.208074495	0.180586486	2.23586E-11				
			Inverse variance weighted	0.934228168	0.291386538	0.001345251	0.003363127			
			Simple mode	1.225891013	0.24387075	0.002387869				
			Weighted mode	1.219915812	0.203628639	0.000972259				
Gallstones	BFP-C2	98	MR Egger	1.028384703	0.300749609	0.000922729		-0.00324856	0.004207994	0.442012301
			Weighted median	0.922110454	0.108751167	2.26971E-17				
			Inverse variance weighted	0.806721558	0.089295439	1.65059E-19	1.65059E-18			
			Simple mode	1.051402341	0.256658347	8.70089E-05				
			Weighted mode	1.051402341	0.228152181	1.23604E-05				
Gallstones	BFP-C3	14	MR	-	0.920859548	0.671084467		0.013236914	0.015499313	0.409812371

			Egger	0.400832675						
			Weighted median	0.412175775	0.219015469	0.059843044				
			Inverse variance weighted	0.369774963	0.181942606	0.042116063	0.1579352			
			Simple mode	-0.082066918	0.447508145	0.857324162				
			Weighted mode	0.5158800 2	0.398713977	0.218229291				
Gallstones	BFP-C4	13	MR Egger	3.686099114	0.867684672	0.00136987		-0.048931098	0.01271531	0.002707886
			Weighted median	0.448912885	0.283647623	0.113502889				
			Inverse variance weighted	0.463373624	0.332917802	0.163966023	0.4471801			
			Simple mode	0.421411813	0.545239726	0.45454088				
			Weighted mode	0.5324404 4	0.480623067	0.289653721				
Gallstones	BFP-C5	9	MR Egger	-1.472304401	0.56311119	0.034681034		0.022586382	0.008880877	0.038480373
			Weighted ed	-0.0068104	0.278224457	0.980471233				

			median	13						
			Inverse variance weighted	-0.124972633	0.223110077	0.575384699	0.7105785			
			Simple mode	0.01604907	0.424034036	0.970735872				
			Weighted mode	0.00436584	0.516107883	0.993457788				
Gallstones	BMI-all	537	MR Egger	0.615263187	0.092251713	6.41858E-11		-0.00210651	0.001487734	0.157381477
			Weighted median	0.511245178	0.045366964	1.86451E-29				
			Inverse variance weighted	0.494043144	0.034396493	8.8086E-47	6.60645E-46			
			Simple mode	0.518416395	0.158326447	0.001127411				
			Weighted mode	0.588526521	0.103852794	2.37626E-08				
Gallstones	BMI-C1	39	MR Egger	0.597513949	0.264425826	0.029819716		0.002310016	0.005453025	0.674294757
			Weighted median	0.843087244	0.11637023	4.32853E-13				
			Inverse variance weighted	0.696301355	0.123305917	1.63327E-08	1.22495E-07			

			Simple mode	0.699711451	0.194255409	0.000901429				
			Weighted mode	0.821104093	0.118281661	2.94716E-08				
Gallstones	BMI-C2	81	MR Egger	0.630447276	0.16742569	0.000318224		-0.0029719	0.003518584	0.400868335
			Weighted median	0.493132806	0.091396363	6.83175E-08				
			Inverse variance weighted	0.501779918	0.069338781	4.59931E-13	2.75959E-12			
			Simple mode	0.423720141	0.189238536	0.027927923				
			Weighted mode	0.508099352	0.107359676	9.41308E-06				
Gallstones	BMI-C3	8	MR Egger	1.563865644	0.535989139	0.026710509		-0.012711607	0.009928219	0.247689187
			Weighted median	1.248822655	0.285389713	1.2096E-05				
			Inverse variance weighted	0.949004022	0.248675812	0.000135506	0.004065191			
			Simple mode	0.801384296	0.579892286	0.209476131				
			Weighted mode	1.384938019	0.29312626	0.002145723				
Gout	BFP-all	384	MR	0.4089864	0.293385927	0.164121284		0.000933727	0.003922498	0.811974559

			Egger	45						
			Weighted median	0.529758603	0.123864354	1.89492E-05				
			Inverse variance weighted	0.475495357	0.089401386	1.04536E-07	2.02161E-07			
			Simple mode	0.592798447	0.425077359	0.16395508				
			Weighted mode	0.853541306	0.305871425	0.005525526				
Gout	BFP-C1	7	MR Egger	1.814900183	0.8711996	0.091686071		-0.024686987	0.019950347	0.270870398
			Weighted median	1.117557146	0.364381184	0.002162188				
			Inverse variance weighted	0.835334896	0.379532995	0.027739237	0.03618161			
			Simple mode	1.374000331	0.604013703	0.063250985				
			Weighted mode	1.199575035	0.41276943	0.027115697				
Gout	BFP-C2	98	MR Egger	1.149462131	0.538653188	0.035394541		-0.006830584	0.007538025	0.367125039
			Weighted median	0.832776035	0.226952825	0.00024314				
			Inverse varianc	0.683456971	0.160084829	1.9605E-05	0.000032675			

			e weighted							
			Simple mode	1.24564922	0.557167377	0.027665285				
			Weighted mode	0.970732769	0.540407578	0.075560156				
Gout	BFP-C3	14	MR Egger	-0.242941728	1.512660758	0.875076066		0.008114865	0.0254588	0.75539955
			Weighted median	0.274626198	0.377978877	0.467492514				
			Inverse variance weighted	0.22949973	0.291448008	0.431020502	0.5896976			
			Simple mode	0.367182376	0.706276378	0.611882489				
			Weighted mode	0.381383443	0.603018428	0.538053583				
Gout	BFP-C4	13	MR Egger	1.777742317	2.116581694	0.418838887		-0.037926119	0.031016597	0.246968785
			Weighted median	-1.318607511	0.584355042	0.024038226				
			Inverse variance weighted	-0.72021628	0.565019235	0.202424057	0.4871924			
			Simple mode	-2.0315186	0.983734015	0.061215325				

				51						
			Weighted mode	-2.031518651	1.0054145	0.066217453				
Gout	BFP-C5	9	MR Egger	-2.520161875	1.449022777	0.125552492		0.031828313	0.022870808	0.206646753
			Weighted median	-0.766041196	0.51774461	0.138987107				
			Inverse variance weighted	-0.623347491	0.519857067	0.230498875	0.3457483			
			Simple mode	-0.376785744	0.73541422	0.622251293				
			Weighted mode	-0.991474385	0.627845306	0.152950797				
Gout	BMI-all	537	MR Egger	0.635800313	0.194648285	0.001159018		-0.002202719	0.003138188	0.483041825
			Weighted median	0.574494505	0.090614266	2.29764E-10				
			Inverse variance weighted	0.508989689	0.072408042	2.07329E-12	3.10994E-12			
			Simple mode	0.620567519	0.287465225	0.031312176				
			Weighted mode	0.693631615	0.177520742	0.000105267				

Gout	BMI-C1	39	MR Egger	0.459968744	0.335211363	0.178271194		0.002800767	0.006910768	0.68760874
			Weighted median	0.664041453	0.235541824	0.004814227				
			Inverse variance weighted	0.579775518	0.156292598	0.000207626	0.000444914			
			Simple mode	0.383544178	0.437342611	0.38600405				
			Weighted mode	0.640733764	0.266399267	0.021141517				
Gout	BMI-C2	81	MR Egger	0.706758204	0.313013762	0.026710619		-0.004722283	0.006571889	0.474533281
			Weighted median	0.52556606	0.171215848	0.002143377				
			Inverse variance weighted	0.502032661	0.129233717	0.000102461	0.000161781			
			Simple mode	0.57183409	0.373929826	0.130145854				
			Weighted mode	0.591149725	0.260990491	0.026215889				
Gout	BMI-C3	8	MR Egger	2.254125987	1.199969268	0.109384308		-0.033237318	0.022278192	0.186319735
			Weighted median	0.884773638	0.488662779	0.070202535				
			Inverse	0.6505018	0.578284039	0.260638608	0.4817201			

			variance weighted	96						
			Simple mode	0.475021333	0.745913411	0.54448987				
			Weighted mode	0.914691784	0.499473678	0.109734205				
Heart failure	BFP-all	384	MR Egger	0.516358986	0.175575716	0.003471312		-0.000683205	0.002347228	0.771156537
			Weighted median	0.569235933	0.075137126	3.56479E-14				
			Inverse variance weighted	0.467690796	0.053501977	2.29911E-18	1.14956E-17			
			Simple mode	0.677300618	0.263819351	0.010628532				
			Weighted mode	0.717126387	0.201536685	0.000420134				
Heart failure	BFP-C1	7	MR Egger	0.819475688	0.489751135	0.155135752		0.005112495	0.011215999	0.667631533
			Weighted median	0.851273026	0.230006124	0.000214677				
			Inverse variance weighted	1.022349907	0.190396729	7.89215E-08	4.73529E-07			
			Simple mode	0.779896234	0.423926769	0.11542683				

			Weighted mode	0.801094229	0.255536459	0.020197076				
Heart failure	BFP-C2	98	MR Egger	0.547393012	0.307378693	0.078101279		-0.000205261	0.004299604	0.962022931
			Weighted median	0.581668839	0.129485237	7.05033E-06				
			Inverse variance weighted	0.533382444	0.090917236	4.44604E-09	1.21256E-08			
			Simple mode	0.657689015	0.384837315	0.090647723				
			Weighted mode	0.732616253	0.354024778	0.041168103				
Heart failure	BFP-C3	14	MR Egger	-0.539471366	1.044332398	0.614842772		0.018379277	0.017576251	0.316312188
			Weighted median	0.339693564	0.257852731	0.187706691				
			Inverse variance weighted	0.530590111	0.2092427	0.011220231	0.08415173			
			Simple mode	0.278237265	0.443311198	0.541111945				
			Weighted mode	0.278237265	0.442684383	0.540547697				
Heart failure	BFP-C4	13	MR Egger	-0.6492935	1.175315879	0.591693888		0.012302917	0.01723581	0.49021949

				57						
			Weighted median	0.577408724	0.332837012	0.082774599				
			Inverse variance weighted	0.160372899	0.301411065	0.594674954	0.784439			
			Simple mode	0.711488274	0.647773726	0.293597023				
			Weighted mode	0.738760789	0.532400168	0.190485599				
Heart failure	BFP-C5	9	MR Egger	0.032133962	0.681049665	0.9636853		-0.000884606	0.010745641	0.936694929
			Weighted median	0.093882471	0.296841689	0.751796686				
			Inverse variance weighted	-0.020606012	0.231070093	0.928941677	0.9289417			
			Simple mode	-0.064688734	0.409550456	0.878409836				
			Weighted mode	0.058788259	0.355593501	0.872790071				
Heart failure	BMI-all	537	MR Egger	0.417225697	0.095744375	1.57628E-05		0.001492993	0.001543419	0.333817023
			Weighted median	0.49941363	0.050514134	4.75862E-23				
			Inverse	0.5031919	0.035623039	2.64655E-45	1.58793E-			

			variance weighted	18			44			
			Simple mode	0.541436742	0.150937145	0.000364893				
			Weighted mode	0.469634943	0.099392161	2.94279E-06				
Heart failure	BMI-C1	39	MR Egger	0.46606701	0.219531413	0.040506296		0.001175462	0.004528243	0.796623223
			Weighted median	0.389162343	0.135661757	0.004122638				
			Inverse variance weighted	0.516320523	0.102240639	4.4169E-07	1.65634E-06			
			Simple mode	0.600903706	0.288237095	0.043863153				
			Weighted mode	0.380108204	0.145032388	0.012541802				
Heart failure	BMI-C2	81	MR Egger	0.297471021	0.163559859	0.072742476		0.004484691	0.003432101	0.195109324
			Weighted median	0.464826642	0.094178816	7.99148E-07				
			Inverse variance weighted	0.492037422	0.067978308	4.54835E-13	2.75959E-12			
			Simple mode	0.547721821	0.194493819	0.006118732				

			Weighted mode	0.510210127	0.15181966	0.0011941				
Heart failure	BMI-C3	8	MR Egger	0.806908279	0.512343017	0.166338713		-0.005166887	0.009504893	0.60630685
			Weighted median	0.581795214	0.280404721	0.038001272				
			Inverse variance weighted	0.557414909	0.227703097	0.014365563	0.08619338			
			Simple mode	0.722364458	0.394044346	0.109429799				
			Weighted mode	0.593510838	0.315937872	0.10238104				
Hypertension	BFP-all	384	MR Egger	0.562982033	0.181096681	0.002019677		8.40595E-05	0.002420606	0.972315853
			Weighted median	0.625291723	0.053544574	1.65201E-31				
			Inverse variance weighted	0.568971518	0.055139498	5.79639E-25	5.49794E-24			
			Simple mode	0.99804719	0.224548373	1.15418E-05				
			Weighted mode	1.06415953	0.27738197	0.000145951				
Hypertension	BFP-C1	7	MR Egger	1.679367046	0.431322741	0.011483982		-0.012233472	0.009871565	0.270243531
			Weight	1.4244910	0.150763921	3.43761E-21				

			ed median	45						
			Inverse variance weighted	1.193644981	0.187920289	2.12715E-10	1.59536E-09			
			Simple mode	1.302405301	0.248687924	0.001943806				
			Weighted mode	1.394895979	0.151334675	9.2007E-05				
Hypertension	BFP-C2	98	MR Egger	0.551065675	0.29018925	0.060568889		0.004878171	0.004058076	0.232285585
			Weighted median	0.906842659	0.095110519	1.50435E-21				
			Inverse variance weighted	0.884163465	0.086371577	1.35732E-24	2.03597E-23			
			Simple mode	1.076089222	0.282435582	0.000243845				
			Weighted mode	1.00071997	0.25612313	0.000172829				
Hypertension	BFP-C3	14	MR Egger	-1.083719149	0.899848651	0.251677124		0.025506782	0.015143196	0.117918611
			Weighted median	0.287569264	0.178610974	0.107391017				
			Inverse variance	0.401440092	0.191948878	0.036493058	0.1563988			

			weighted							
			Simple mode	0.089805192	0.291533096	0.762928555				
			Weighted mode	0.079666751	0.243540961	0.748785765				
Hypertension	BFP-C4	13	MR Egger	1.252104596	1.117298814	0.286305738		-0.022132005	0.016380446	0.203790988
			Weighted median	-0.39321603	0.243463304	0.106290422				
			Inverse variance weighted	-0.20482613	0.302476342	0.498301821	0.7474527			
			Simple mode	-0.482104897	0.445559941	0.300507915				
			Weighted mode	-0.380495042	0.380968145	0.337626203				
Hypertension	BFP-C5	9	MR Egger	-0.092620602	0.677288119	0.89507677		-0.017000388	0.010687898	0.155720792
			Weighted median	-0.921188843	0.209356886	1.08207E-05				
			Inverse variance weighted	-1.10590842	0.251033105	1.05575E-05	6.3345E-05			
			Simple mode	-0.8037138	0.352526174	0.052081891				

				51						
			Weighted mode	-0.767844917	0.223031381	0.008787918				
Hypertension	BMI-all	537	MR Egger	0.603720956	0.086853077	1.05932E-11		-0.000578542	0.001399992	0.679591641
			Weighted median	0.545403996	0.036005072	7.81352E-52				
			Inverse variance weighted	0.570403654	0.032275729	6.78964E-70	6.78964E-69			
			Simple mode	0.441391343	0.131566151	0.000850139				
			Weighted mode	0.583781352	0.082655434	5.0878E-12				
Hypertension	BMI-C1	39	MR Egger	0.484824607	0.222459627	0.035743682		0.007770131	0.00458707	0.098683896
			Weighted median	0.725109531	0.098101847	1.45296E-13				
			Inverse variance weighted	0.817142642	0.107432543	2.82491E-14	2.82491E-13			
			Simple mode	0.772492297	0.184799579	0.000164864				
			Weighted mode	0.705130966	0.093327671	4.41389E-09				
Hypertension	BMI-C2	81	MR Egger	0.435041007	0.143232734	0.003233846		0.004050796	0.003004795	0.181475644

			Weighted median	0.544088889	0.07471662	3.28827E-13				
			Inverse variance weighted	0.610870587	0.059500466	9.96012E-25	1.49402E-23			
			Simple mode	0.610132692	0.174548836	0.0007748				
			Weighted mode	0.427739078	0.102426704	7.50272E-05				
Hypertension	BMI-C3	8	MR Egger	0.602712614	0.511146277	0.282977198		-0.012330671	0.009482031	0.241168691
			Weighted median	0.30722987	0.21357489	0.150289063				
			Inverse variance weighted	0.007250852	0.23810625	0.975706439	0.9757064			
			Simple mode	-0.270071394	0.336814484	0.449004929				
			Weighted mode	0.476214311	0.232954717	0.080209919				
Ischemic heart disease	BFP-all	384	MR Egger	0.052865404	0.16837531	0.753712471		0.002551727	0.002250678	0.257606556
			Weighted median	0.2767139	0.058467039	2.21423E-06				
			Inverse varianc	0.23467044	0.051363224	4.90427E-06	6.68764E-06			

			e weighted							
			Simple mode	0.135930881	0.248636597	0.584899751				
			Weighted mode	0.390158807	0.160767242	0.01569091				
Ischemic heart disease	BFP-C1	7	MR Egger	0.601121757	0.52984312	0.308017785		-0.003534452	0.012125453	0.782382165
			Weighted median	0.540897536	0.16600562	0.001120785				
			Inverse variance weighted	0.46077561	0.203600811	0.023627523	0.03457335			
			Simple mode	0.622775468	0.260723209	0.054126072				
			Weighted mode	0.559149461	0.17123436	0.017132471				
Ischemic heart disease	BFP-C2	98	MR Egger	0.068943527	0.277182232	0.80410101		0.006408833	0.003876405	0.101537955
			Weighted median	0.387538203	0.106358766	0.000268759				
			Inverse variance weighted	0.506508077	0.083096319	1.09141E-09	3.27424E-09			
			Simple mode	0.281452395	0.30444237	0.357528038				
			Weight	0.1928825	0.276050356	0.48639746				

			Weighted mode	56						
Ischemic heart disease	BFP-C3	14	MR Egger	1.12008799	1.186981531	0.363963515		-0.014167361	0.019977373	0.491767893
			Weighted median	0.240383116	0.197834341	0.224338347				
			Inverse variance weighted	0.295255755	0.232356817	0.203835476	0.4255185			
			Simple mode	0.175489425	0.346636887	0.621148334				
			Weighted mode	0.310136665	0.315001983	0.342813062				
Ischemic heart disease	BFP-C4	13	MR Egger	0.93616795	1.075667213	0.402722148		-0.017324625	0.015765754	0.295291022
			Weighted median	-0.230863767	0.246521509	0.349023384				
			Inverse variance weighted	-0.204657253	0.283947105	0.471058191	0.7437761			
			Simple mode	-0.666487294	0.406196511	0.126769407				
			Weighted mode	-0.560648621	0.436162006	0.222901537				
Ischemic heart disease	BFP-C5	9	MR Egger	-1.582698078	1.036365144	0.170559031		0.011361774	0.016349064	0.50948989

			Weighted median	-1.117528945	0.260671526	1.81008E-05				
			Inverse variance weighted	-0.905239226	0.340231299	0.007798806	0.02753962			
			Simple mode	-1.457916874	0.349660543	0.003124153				
			Weighted mode	-1.316491952	0.282762881	0.001632296				
Ischemic heart disease	BMI-all	537	MR Egger	0.296093338	0.080229445	0.000246662		-0.000303889	0.001293271	0.814316391
			Weighted median	0.308994394	0.043309359	9.70781E-13				
			Inverse variance weighted	0.278594362	0.029820586	9.42191E-21	3.14064E-20			
			Simple mode	0.315892579	0.151504115	0.037538021				
			Weighted mode	0.377496062	0.087211177	1.79151E-05				
Ischemic heart disease	BMI-C1	39	MR Egger	0.326327805	0.189066866	0.092692161		0.003278206	0.003898773	0.405844348
			Weighted median	0.368985069	0.102521967	0.000319341				
			Inverse varianc	0.466515212	0.088813082	1.49824E-07	7.49119E-07			

			e weighted							
			Simple mode	0.432806882	0.19463096	0.032186418				
			Weighted mode	0.386324945	0.098038342	0.000336727				
Ischemic heart disease	BMI-C2	81	MR Egger	-0.027342035	0.147331451	0.853248394		0.007624734	0.003091545	0.015816334
			Weighted median	0.301365945	0.07942482	0.000148028				
			Inverse variance weighted	0.303483562	0.062846316	1.37235E-06	4.11704E-06			
			Simple mode	0.312298476	0.190843443	0.105681773				
			Weighted mode	0.312298476	0.136906809	0.025201882				
Ischemic heart disease	BMI-C3	8	MR Egger	0.503411741	0.552140868	0.397054962		-0.005581185	0.010235236	0.605215125
			Weighted median	0.573921286	0.219941123	0.009069281				
			Inverse variance weighted	0.2336777	0.232659964	0.315198209	0.4817201			
			Simple mode	0.340334177	0.51638735	0.530927194				

			Weighted mode	0.627373151	0.236334804	0.032722747				
Intrahepatic liver and bile duct cancer	BFP-all	384	MR Egger	0.522041193	0.817175876	0.523312608		-0.002714609	0.010922931	0.803862629
			Weighted median	0.025377561	0.390431828	0.948175035				
			Inverse variance weighted	0.328626135	0.248883334	0.186700725	0.1931387			
			Simple mode	-0.443452867	1.289143365	0.731042325				
			Weighted mode	-0.344525122	0.963931687	0.720976867				
Intrahepatic liver and bile duct cancer	BFP-C1	7	MR Egger	-0.045761817	2.415435341	0.985617297		0.037728192	0.055309388	0.525467838
			Weighted median	1.465975038	1.229306934	0.233056797				
			Inverse variance weighted	1.451357002	1.008617839	0.150162902	0.1732649			
			Simple mode	3.074818309	1.94898233	0.165720554				
			Weighted mode	0.796140612	1.2990237	0.562452705				
Intrahepatic liver and bile duct cancer	BFP-C2	98	MR	1.5304072	1.630991412	0.350429941		-0.01041006	0.022812718	0.649185526

			Egger	45						
			Weighted median	1.257300901	0.746418164	0.092095556				
			Inverse variance weighted	0.819797869	0.482935317	0.089596238	0.09955138			
			Simple mode	2.180318121	1.831003847	0.236646819				
			Weighted mode	1.350924547	1.524436385	0.377712451				
Intrahepatic liver and bile duct cancer	BFP-C3	14	MR Egger	-1.131422358	5.959168082	0.852590666		0.047541208	0.100282295	0.643956701
			Weighted median	0.219828333	1.408859299	0.876007127				
			Inverse variance weighted	1.636733037	1.154263097	0.156193809	0.3904845			
			Simple mode	-1.719540781	2.92957684	0.56728567				
			Weighted mode	-1.253273761	2.37128201	0.606043243				
Intrahepatic liver and bile duct cancer	BFP-C4	13	MR Egger	6.736801979	4.46282659	0.159338421		-0.134082652	0.065403805	0.06496737
			Weighted median	-0.936702566	1.498407127	0.531884286				

			Inverse variance weighted	-2.094465889	1.166128503	0.072481003	0.2718038			
			Simple mode	-0.680402726	2.379053271	0.779757408				
			Weighted mode	-0.585197681	2.00123951	0.774961699				
Intrahepatic liver and bile duct cancer	BFP-C5	9	MR Egger	-4.082704481	3.731243916	0.310086169		0.024778007	0.058823815	0.686223772
			Weighted median	-2.348232914	1.706305195	0.168756873				
			Inverse variance weighted	-2.603956958	1.264079848	0.039402135	0.07880427			
			Simple mode	-2.031180769	2.44386543	0.43000386				
			Weighted mode	-2.092741482	2.120018956	0.352488302				
Intrahepatic liver and bile duct cancer	BMI-all	537	MR Egger	0.963890753	0.459385942	0.036354455		-0.011012767	0.007404516	0.137523632
			Weighted median	0.329130995	0.283834861	0.246217312				
			Inverse variance	0.32967787	0.17088982	0.05370805	0.05556005			

			weighted							
			Simple mode	0.253885029	0.931363604	0.785269566				
			Weighted mode	0.776055536	0.61251456	0.205705764				
Intrahepatic liver and bile duct cancer	BMI-C1	39	MR Egger	1.257792611	1.031274102	0.230315661		-0.000402129	0.021273265	0.985020022
			Weighted median	1.193145342	0.794840705	0.133326459				
			Inverse variance weighted	1.240601675	0.486280831	0.010735142	0.01400236			
			Simple mode	2.207453209	1.254014981	0.086403152				
			Weighted mode	1.512436972	0.753195018	0.051787863				
Intrahepatic liver and bile duct cancer	BMI-C2	81	MR Egger	0.633049953	0.927079233	0.496703388		-0.00648186	0.019451198	0.739838166
			Weighted median	-0.104108072	0.556765116	0.851670498				
			Inverse variance weighted	0.351802484	0.381481188	0.356423536	0.368714			
			Simple mode	0.071765294	1.093284841	0.947826691				
			Weighted	0.206631754	0.859470457	0.81062162				

			mode							
Intrahepatic liver and bile duct cancer	BMI-C3	8	MR Egger	1.794647127	2.857078558	0.553054061		-0.03572152	0.052876841	0.524487009
			Weighted median	0.362308884	1.551792744	0.815390542				
			Inverse variance weighted	0.065140666	1.246525865	0.958323299	0.9757064			
			Simple mode	-2.760644811	2.716164433	0.343285716				
			Weighted mode	0.516958518	1.719480275	0.77241592				
Non-alcoholic fatty liver disease	BFP-all	384	MR Egger	1.142357106	0.53284882	0.032674262		-0.009071705	0.007125243	0.203728969
			Weighted median	0.707791658	0.219502392	0.001261804				
			Inverse variance weighted	0.496358201	0.16286644	0.002306464	0.002661305			
			Simple mode	1.205686969	0.801482431	0.133322859				
			Weighted mode	1.352922646	0.525023572	0.01034311				
Non-alcoholic fatty liver disease	BFP-C1	7	MR Egger	2.677384622	1.394698033	0.112973905		-0.021184418	0.032027442	0.537585207
			Weighted	2.050947174	0.675809295	0.002406939				

			median							
			Inverse variance weighted	1.839445117	0.583373049	0.001615355	0.003727742			
			Simple mode	2.138655302	1.058059566	0.089738731				
			Weighted mode	2.092705146	0.766509015	0.034178				
Non-alcoholic fatty liver disease	BFP-C2	98	MR Egger	2.251626545	0.903176703	0.014378506		-0.012135112	0.012633119	0.339176925
			Weighted median	1.750988702	0.403666262	1.43972E-05				
			Inverse variance weighted	1.42326918	0.268472271	1.14941E-07	2.87352E-07			
			Simple mode	2.042099217	0.963160498	0.036540149				
			Weighted mode	1.97232264	0.788734683	0.014074296				
Non-alcoholic fatty liver disease	BFP-C3	14	MR Egger	3.575815726	2.744063082	0.216987096		-0.055946667	0.046175518	0.248981079
			Weighted median	0.692094845	0.723973126	0.339088609				
			Inverse variance weighted	0.318020372	0.54785175	0.561586801	0.7230202			

			Simple mode	0.401577158	1.204516018	0.744153715				
			Weighted mode	0.847747874	1.058218534	0.437466376				
Non-alcoholic fatty liver disease	BFP-C4	13	MR Egger	3.673816341	3.031831976	0.250995934		-0.065309109	0.044419139	0.169498535
			Weighted median	-0.324432065	1.008778331	0.747749018				
			Inverse variance weighted	-0.628681124	0.830498138	0.449054167	0.7437761			
			Simple mode	0.893646534	1.892125528	0.645187488				
			Weighted mode	0.516078033	1.770898841	0.775705105				
Non-alcoholic fatty liver disease	BFP-C5	9	MR Egger	-4.232157925	2.157886707	0.090652417		0.008826517	0.034074472	0.803059702
			Weighted median	-3.75133467	0.929928644	5.48347E-05				
			Inverse variance weighted	-3.70644467	0.733219792	4.30333E-07	3.2275E-06			
			Simple mode	-3.633352759	1.363809066	0.028621958				
			Weighted	-3.7213676	1.153291038	0.012116114				

			mode	11						
Non-alcoholic fatty liver disease	BMI-all	537	MR Egger	0.763402255	0.284149959	0.007442398		-0.000756859	0.004581021	0.86883628
			Weighted median	0.773556093	0.174908345	9.75036E-06				
			Inverse variance weighted	0.719825831	0.105615892	9.3932E-12	1.28089E-11			
			Simple mode	0.33761902	0.494126683	0.494735211				
			Weighted mode	0.657057337	0.294133094	0.025902808				
Non-alcoholic fatty liver disease	BMI-C1	39	MR Egger	0.534183943	0.600129286	0.379155613		0.011874489	0.01237647	0.343564848
			Weighted median	0.796403725	0.460442854	0.083693315				
			Inverse variance weighted	1.041946412	0.282972277	0.000231275	0.00046255			
			Simple mode	0.693142499	0.714868316	0.338372969				
			Weighted mode	0.747023554	0.436848002	0.095414861				
Non-alcoholic fatty liver disease	BMI-C2	81	MR Egger	0.481511453	0.495099534	0.333743072		0.004974678	0.010392447	0.63348701
			Weighted median	0.671701751	0.330675797	0.04222457				

			Inverse variance weighted	0.697300853	0.203897693	0.000626542	0.000939813			
			Simple mode	0.361902282	0.640372891	0.573557957				
			Weighted mode	0.601826171	0.478618238	0.212258446				
Non-alcoholic fatty liver disease	BMI-C3	8	MR Egger	3.685055815	1.62985759	0.064462371		-0.079235527	0.030231966	0.039537536
			Weighted median	-0.413385556	1.004036135	0.680541891				
			Inverse variance weighted	-0.141654732	0.921093856	0.877775569	0.9425136			
			Simple mode	-1.555400007	1.72268049	0.396586796				
			Weighted mode	2.117583237	1.033824178	0.079730063				
Osteoporosis	BFP-all	384	MR Egger	-0.121053851	0.279148832	0.664784912		0.001159259	0.003731408	0.756216463
			Weighted median	-0.067683934	0.126870328	0.593695074				
			Inverse variance weight	-0.0384601	0.085028168	0.651037125	0.6510371			

			ed							
			Simple mode	0.014231476	0.456278383	0.975133994				
			Weighted mode	-0.393539921	0.398083283	0.323489417				
Osteoporosis	BFP-C1	7	MR Egger	-0.571078103	1.013835999	0.597563636		-0.001775134	0.023192903	0.941959464
			Weighted median	-0.868930701	0.398474126	0.029209667				
			Inverse variance weighted	-0.641595668	0.386436959	0.096857037	0.1210713			
			Simple mode	-1.083122135	0.719371578	0.182866939				
			Weighted mode	-0.949922823	0.420813931	0.064783885				
Osteoporosis	BFP-C2	98	MR Egger	-0.032547242	0.520012961	0.950223689		-0.001878309	0.00727029	0.796686703
			Weighted median	-0.212608747	0.227232661	0.349456806				
			Inverse variance weighted	-0.160838412	0.153627548	0.295128494	0.2951285			
			Simple mode	-0.4980007	0.581977264	0.394269909				

				21						
			Weighted mode	-0.357504093	0.55018896	0.517367482				
Osteoporosis	BFP-C3	14	MR Egger	1.381704996	1.541314733	0.387644996		-0.024503801	0.025938489	0.363451027
			Weighted median	-0.054964983	0.438595332	0.900269829				
			Inverse variance weighted	-0.045049683	0.307690625	0.883595941	0.9140648			
			Simple mode	-0.040436264	0.682633295	0.953665279				
			Weighted mode	-0.446199523	0.70932828	0.540216246				
Osteoporosis	BFP-C4	13	MR Egger	0.509490635	1.91071466	0.794671689		0.000692497	0.028005349	0.98071535
			Weighted median	1.341403069	0.544999722	0.013843792				
			Inverse variance weighted	0.555089109	0.479011924	0.246529128	0.5168328			
			Simple mode	1.5287647	0.887107512	0.110475665				
			Weighted mode	1.479868242	0.705470165	0.057779902				
Osteoporosis	BFP-C5	9	MR	-	1.214818033	0.821949912		0.007327075	0.019166085	0.713585491

			Egger	0.283830359						
			Weighted median	-0.028427994	0.54781395	0.958613538				
			Inverse variance weighted	0.153010829	0.412376944	0.710603246	0.8085036			
			Simple mode	-0.048325936	0.848199512	0.955962659				
			Weighted mode	-0.192816239	0.725667604	0.79718262				
Osteoporosis	BMI-all	537	MR Egger	-0.197180201	0.171396646	0.250479897		0.000156396	0.002762583	0.95487525
			Weighted median	-0.1733718	0.096077988	0.071154474				
			Inverse variance weighted	-0.188173219	0.06369103	0.003132069	0.003355788			
			Simple mode	0.146426538	0.290806132	0.614805986				
			Weighted mode	-0.281454434	0.215578739	0.192256874				
Osteoporosis	BMI-C1	39	MR Egger	-0.22824193	0.33737313	0.502913904		-0.003906524	0.006955047	0.577719452
			Weight	-	0.266585007	0.144665889				

			Weighted median	0.388849937						
			Inverse variance weighted	-0.395359065	0.15904916	0.012927277	0.0161591			
			Simple mode	-0.037747067	0.44666032	0.93309497				
			Weighted mode	-0.423951665	0.285453689	0.145744015				
Osteoporosis	BMI-C2	81	MR Egger	-0.187634584	0.279101652	0.503366711		-0.000991027	0.005854692	0.866016476
			Weighted median	-0.242438477	0.180851686	0.180070644				
			Inverse variance weighted	-0.230649077	0.114719928	0.044374	0.04930444			
			Simple mode	-0.692092926	0.395123331	0.083677291				
			Weighted mode	-0.540221347	0.275672104	0.053519168				
Osteoporosis	BMI-C3	8	MR Egger	1.080041183	1.467257453	0.489439172		-0.0077563	0.027186912	0.785010284
			Weighted median	0.781929137	0.524073492	0.135693399				
			Inverse	0.7050072	0.607506763	0.24584824	0.4817201			

			variance weighted							
			Simple mode	1.008540539	0.892465659	0.295680309				
			Weighted mode	0.818816021	0.568061801	0.192663848				
Peripheral artery disease	BFP-all	386	MR Egger	0.604795368	0.253373713	0.017471128		-0.001524759	0.003399346	0.6540131
			Weighted median	0.638882005	0.10775166	3.04399E-09				
			Inverse variance weighted	0.496602739	0.077488067	1.46704E-10	3.38548E-10			
			Simple mode	0.774326407	0.333904459	0.020917512				
			Weighted mode	0.82745133	0.236496007	0.000522				
Peripheral artery disease	BFP-C1	7	MR Egger	0.677521919	0.591472417	0.30385892		-0.003054582	0.013539329	0.830437924
			Weighted median	0.631845349	0.296857133	0.033299851				
			Inverse variance weighted	0.556252038	0.246793413	0.024201347	0.03457335			
			Simple mode	0.637410881	0.465255709	0.219732235				

			Weighted mode	0.637410881	0.296616673	0.075216443				
Peripheral artery disease	BFP-C2	99	MR Egger	0.668899847	0.434211811	0.126696479		0.003228544	0.006144606	0.600485204
			Weighted median	0.963053151	0.179832011	8.54178E-08				
			Inverse variance weighted	0.886456736	0.130270328	1.01226E-11	3.79599E-11			
			Simple mode	0.982949738	0.458006804	0.034330552				
			Weighted mode	1.029471871	0.412605225	0.01426539				
Peripheral artery disease	BFP-C3	14	MR Egger	0.610512272	1.38947261	0.668191235		0.004386542	0.023387171	0.854354169
			Weighted median	1.083988138	0.320837988	0.000728519				
			Inverse variance weighted	0.865880724	0.266835268	0.001174502	0.02322089			
			Simple mode	1.384789135	0.511642742	0.017967951				
			Weighted mode	1.215969376	0.441068456	0.016322085				
Peripheral artery disease	BFP-C4	13	MR Egger	0.733602716	1.185785857	0.548734809		-0.016203998	0.017388746	0.371406972
			Weight	0.0508357	0.394153876	0.897377877				

			Weighted median	74						
			Inverse variance weighted	-0.332860818	0.308697222	0.280910588	0.5267074			
			Simple mode	0.321784104	0.64943475	0.629207999				
			Weighted mode	0.217615279	0.547278094	0.697884				
Peripheral artery disease	BFP-C5	9	MR Egger	-1.323862906	1.643910483	0.447126012		-0.0028023	0.025894673	0.916858497
			Weighted median	-1.627554536	0.41893541	0.000102339				
			Inverse variance weighted	-1.49126956	0.520809155	0.004191601	0.01796401			
			Simple mode	-1.658495349	0.598875172	0.024321053				
			Weighted mode	-1.632962625	0.460136663	0.007521893				
Peripheral artery disease	BMI-all	535	MR Egger	0.542982946	0.136779229	8.18048E-05		-0.00178489	0.002209577	0.41956782
			Weighted median	0.435877474	0.070728229	7.1511E-10				
			Inverse varianc	0.440514632	0.051148433	7.14995E-18	1.64999E-17			

			e weighted							
			Simple mode	0.290256867	0.218588796	0.18478933				
			Weighted mode	0.367865126	0.134347334	0.006384523				
Peripheral artery disease	BMI-C1	39	MR Egger	-0.008284046	0.32941222	0.980072088		0.0159635	0.006789916	0.024157701
			Weighted median	0.209732165	0.181307103	0.247362854				
			Inverse variance weighted	0.674778252	0.164238352	3.98185E-05	0.000132728			
			Simple mode	0.634562708	0.387472351	0.109740416				
			Weighted mode	0.226201385	0.183603079	0.225513523				
Peripheral artery disease	BMI-C2	81	MR Egger	0.436016484	0.242793304	0.076344395		0.002269336	0.005136627	0.65984458
			Weighted median	0.497490532	0.1342852	0.000211615				
			Inverse variance weighted	0.533656207	0.100014482	9.51298E-08	3.56737E-07			
			Simple mode	0.333505716	0.26435147	0.210759065				

			Weighted mode	0.461844511	0.190517886	0.0175995				
Peripheral artery disease	BMI-C3	7	MR Egger	1.032284573	0.703533505	0.202217918		-0.031267026	0.013619184	0.070138661
			Weighted median	0.007147427	0.384919022	0.985185211				
			Inverse variance weighted	-0.414776916	0.313258923	0.185479614	0.4006927			
			Simple mode	-0.810335989	0.677252249	0.276629574				
			Weighted mode	0.003075121	0.398614485	0.994094865				
Polycystic ovary syndrome	BFP-all	384	MR Egger	1.314216177	0.629220014	0.037401373		-0.005296686	0.008411022	0.529246654
			Weighted median	0.774964347	0.298335572	0.009387058				
			Inverse variance weighted	0.936833856	0.191649465	1.01727E-06	1.69545E-06			
			Simple mode	1.299423074	0.95631408	0.175015106				
			Weighted mode	1.011134744	0.698989036	0.148836007				
Polycystic ovary syndrome	BFP-C1	7	MR Egger	-0.1791517	1.864280564	0.927176345		0.03539982	0.042625763	0.444100458

				13						
			Weighted median	0.81333851	0.882690779	0.356825531				
			Inverse variance weighted	1.227795675	0.757710095	0.105145746	0.1261749			
			Simple mode	0.969460962	1.718767377	0.593165197				
			Weighted mode	0.610212816	1.00120549	0.56455818				
Polycystic ovary syndrome	BFP-C2	98	MR Egger	4.058745696	1.147629632	0.000626003		-0.037983123	0.016050454	0.019966288
			Weighted median	1.46474943	0.510544795	0.004117809				
			Inverse variance weighted	1.465361689	0.340731732	1.70312E-05	3.0055E-05			
			Simple mode	0.923070363	1.199833512	0.443565565				
			Weighted mode	1.339005895	1.070700086	0.214092728				
Polycystic ovary syndrome	BFP-C3	14	MR Egger	-0.776215403	3.481512187	0.827321646		0.028957918	0.05857702	0.629980341
			Weighted median	0.977370691	0.937665473	0.297251868				
			Inverse	0.9101925	0.695569158	0.190684138	0.4255185			

			variance weighted	42							
			Simple mode	2.111968985	1.617039347	0.21416635					
			Weighted mode	1.636466986	1.501495343	0.295551712					
Polycystic ovary syndrome	BFP-C4	13	MR Egger	2.417474313	3.903078899	0.54828229			-0.04044434	0.057125048	0.493676491
			Weighted median	-1.07116209	1.247207329	0.39042413					
			Inverse variance weighted	-0.24980644	0.998864266	0.802517381	0.891686				
			Simple mode	-2.679666688	2.755463074	0.35000124					
			Weighted mode	-2.725954053	2.291246677	0.25716145					
Polycystic ovary syndrome	BFP-C5	9	MR Egger	-3.221896197	2.741287417	0.278289734			0.023192168	0.043266428	0.608540111
			Weighted median	-2.254456231	1.197257134	0.059697986					
			Inverse variance weighted	-1.840046133	0.932174482	0.04838983	0.0904727				

			Simple mode	-2.232663948	2.064567837	0.311028322				
			Weighted mode	-2.824430373	1.780804395	0.151390603				
Polycystic ovary syndrome	BMI-all	537	MR Egger	1.187633864	0.354076493	0.000852438		-0.004406038	0.005709163	0.44060415
			Weighted median	0.909123103	0.203226968	7.69721E-06				
			Inverse variance weighted	0.934005966	0.131728532	1.3374E-12	2.11168E-12			
			Simple mode	1.528166768	0.754761032	0.043393171				
			Weighted mode	1.297338917	0.565605164	0.022192707				
Polycystic ovary syndrome	BMI-C1	39	MR Egger	-0.493599788	0.76146284	0.520841781		0.033478903	0.015708095	0.039771034
			Weighted median	0.295609867	0.546032424	0.588247162				
			Inverse variance weighted	0.937321827	0.359270238	0.009081775	0.01238424			
			Simple mode	1.043549983	1.098438757	0.348100409				
			Weighted	0.072390547	0.582785315	0.901799975				

			mode							
Polycystic ovary syndrome	BMI-C2	81	MR Egger	1.406990397	0.780971561	0.075425201		-0.002291416	0.01640378	0.88926178
			Weighted median	1.49390523	0.418652288	0.000359212				
			Inverse variance weighted	1.307688682	0.321377874	4.7214E-05	8.85263E-05			
			Simple mode	1.816571726	0.887185132	0.043884031				
			Weighted mode	1.96920978	0.574829084	0.000970356				
Polycystic ovary syndrome	BMI-C3	8	MR Egger	1.816043258	3.390338572	0.61146239		-0.023112235	0.062714	0.725128843
			Weighted median	1.119651761	1.189189244	0.34643573				
			Inverse variance weighted	0.696363987	1.408642622	0.621058264	0.7763228			
			Simple mode	-0.629665894	2.395965291	0.800269663				
			Weighted mode	1.071011465	1.368082533	0.459372244				
Psoriasis	BFP-all	384	MR Egger	0.191518131	0.276831815	0.489469856		0.003606891	0.003700541	0.330329349
			Weighted ed	0.303648593	0.123417061	0.013880337				

			median							
			Inverse variance weighted	0.448492133	0.084413673	1.07819E-07	2.02161E-07			
			Simple mode	1.006634416	0.413353752	0.015335587				
			Weighted mode	0.241927996	0.292854164	0.409259405				
Psoriasis	BFP-C1	7	MR Egger	0.357627371	0.801037378	0.673940348		-0.006554287	0.018326862	0.735219009
			Weighted median	0.196520087	0.335590328	0.558147571				
			Inverse variance weighted	0.097286251	0.30902895	0.752903836	0.778866			
			Simple mode	0.346101836	0.521145729	0.531292623				
			Weighted mode	0.240129973	0.371251203	0.541690887				
Psoriasis	BFP-C2	98	MR Egger	1.32097185	0.540931482	0.016435066		-0.010251375	0.007563615	0.178485212
			Weighted median	0.547787566	0.201756874	0.006625826				
			Inverse variance weighted	0.620923946	0.161405199	0.000119579	0.000188808			

			Simple mode	0.49420391	0.527388524	0.351046009				
			Weighted mode	0.408470438	0.401735794	0.311795162				
Psoriasis	BFP-C3	14	MR Egger	2.381603758	2.318363185	0.324551986		-0.033532524	0.03902844	0.407076359
			Weighted median	-0.060892844	0.453843046	0.893266889				
			Inverse variance weighted	0.429873961	0.458534534	0.348503851	0.5808398			
			Simple mode	-0.749785697	0.693709925	0.299414234				
			Weighted mode	-0.731602494	0.652393292	0.282399683				
Psoriasis	BFP-C4	13	MR Egger	-1.806195001	1.489663193	0.250724818		0.023883957	0.021842713	0.297562712
			Weighted median	-0.442041819	0.51286788	0.388741484				
			Inverse variance weighted	-0.234167084	0.393280662	0.551562909	0.784439			
			Simple mode	-0.337481591	0.946854757	0.727711413				
			Weight	-	0.771727798	0.635639069				

			ed mode	0.375151384						
Psoriasis	BFP-C5	9	MR Egger	-1.580620142	1.062387998	0.180403955		0.020552534	0.01676403	0.259842035
			Weighted median	-0.657134934	0.461235693	0.154236138				
			Inverse variance weighted	-0.355504907	0.360686675	0.324312943	0.4633042			
			Simple mode	-0.49688083	0.661212723	0.473894888				
			Weighted mode	-0.652193388	0.602408268	0.310516347				
Psoriasis	BMI-all	537	MR Egger	0.596594448	0.160245576	0.000217737		-0.003153243	0.002583181	0.222744118
			Weighted median	0.394539866	0.082760647	1.86767E-06				
			Inverse variance weighted	0.415016027	0.059622589	3.38543E-12	4.83633E-12			
			Simple mode	0.29785997	0.325769527	0.360956009				
			Weighted mode	0.215677369	0.272239635	0.428574999				
Psoriasis	BMI-C1	39	MR Egger	-0.3028848	0.559971855	0.591823136		0.018750185	0.011550407	0.113008398

				38						
			Weighted median	0.023977914	0.212710327	0.910248051				
			Inverse variance weighted	0.498599198	0.269826199	0.064623586	0.07180398			
			Simple mode	0.404648143	0.474893985	0.399507546				
			Weighted mode	0.03699043	0.201940054	0.855634338				
Psoriasis	BMI-C2	81	MR Egger	0.681646022	0.259865271	0.010453727		-0.005972157	0.005448857	0.27639199
			Weighted median	0.413673045	0.161383855	0.010368647				
			Inverse variance weighted	0.422255344	0.107471071	8.52949E-05	0.000142158			
			Simple mode	0.347205872	0.430316206	0.422138203				
			Weighted mode	0.701390261	0.283298873	0.015405257				
Psoriasis	BMI-C3	8	MR Egger	0.570516769	0.850738903	0.527422409		-0.009461075	0.015778508	0.570694844
			Weighted median	-0.363852836	0.457432105	0.426366445				
			Inverse varianc	0.113547596	0.36038777	0.75270804	0.9032496			

			e weighted							
			Simple mode	0.188773931	0.819296666	0.824362258				
			Weighted mode	-0.337374593	0.517531113	0.535280647				
Pulmonary embolism	BFP-all	384	MR Egger	0.900860763	0.264358889	0.000724776		-0.006760049	0.003534076	0.056518607
			Weighted median	0.466739139	0.116459631	6.13043E-05				
			Inverse variance weighted	0.419286642	0.080913257	2.19604E-07	3.87537E-07			
			Simple mode	0.1959765 4	0.390326426	0.615897159				
			Weighted mode	0.487735783	0.275406184	0.077361151				
Pulmonary embolism	BFP-C1	7	MR Egger	0.884384798	0.68942676	0.2558127		-0.006181533	0.0157707	0.711234954
			Weighted median	0.6358882 7	0.336974456	0.059153352				
			Inverse variance weighted	0.638795033	0.287623927	0.026355132	0.0359388 2			
			Simple mode	0.422872696	0.543116861	0.465800943				
			Weight	0.6314673	0.366783828	0.135917282				

			ed mode	98						
Pulmonary embolism	BFP-C2	98	MR Egger	0.627883713	0.48081538	0.194716405		-0.001584285	0.006727162	0.814317951
			Weighted median	0.601438106	0.204380716	0.003253277				
			Inverse variance weighted	0.519774351	0.142321622	0.000260093	0.000371562			
			Simple mode	0.749179826	0.513172477	0.147549124				
			Weighted mode	0.788628142	0.459987776	0.08963877				
Pulmonary embolism	BFP-C3	14	MR Egger	-1.512309778	1.346085181	0.283204489		0.025815802	0.022654135	0.276705085
			Weighted median	-0.137685141	0.354109815	0.697408966				
			Inverse variance weighted	-0.00923822	0.268720284	0.972575269	0.9725753			
			Simple mode	-0.398614498	0.590436419	0.511430253				
			Weighted mode	-0.360847366	0.481896188	0.467306424				
Pulmonary embolism	BFP-C4	13	MR Egger	4.379008326	1.736028234	0.028352737		-0.056561678	0.025446102	0.048128771

			Weighted median	0.937362574	0.525716436	0.074583154				
			Inverse variance weighted	0.654529095	0.523413612	0.211116695	0.4871924			
			Simple mode	-0.990655043	1.234280301	0.437803582				
			Weighted mode	1.599750496	0.7744459	0.061154576				
Pulmonary embolism	BFP-C5	9	MR Egger	2.187014675	1.191138583	0.1089711		-0.019644073	0.018800407	0.330815006
			Weighted median	1.456635517	0.479110169	0.002363477				
			Inverse variance weighted	1.016327047	0.406656214	0.012446381	0.03111595			
			Simple mode	1.816050188	0.679767117	0.028292993				
			Weighted mode	1.678947363	0.601804829	0.023563631				
Pulmonary embolism	BMI-all	537	MR Egger	0.347964236	0.141699655	0.014380097		-0.001022081	0.002284619	0.654785424
			Weighted median	0.172200487	0.086538205	0.046604356				
			Inverse varianc	0.289125598	0.052699143	4.10339E-08	5.35224E-08			

			e weighted							
			Simple mode	-0.048847171	0.283630303	0.863328583				
			Weighted mode	0.143966185	0.17990652	0.423933392				
Pulmonary embolism	BMI-C1	39	MR Egger	0.116042371	0.294394273	0.695715825		0.008517916	0.006070825	0.168926392
			Weighted median	0.167192904	0.212424853	0.431241787				
			Inverse variance weighted	0.480313148	0.140531646	0.000631227	0.000996675			
			Simple mode	0.445556426	0.407414224	0.281005925				
			Weighted mode	0.251396467	0.224469757	0.269761022				
Pulmonary embolism	BMI-C2	81	MR Egger	0.490093822	0.248532754	0.052115557		-0.003781295	0.005217524	0.470758901
			Weighted median	0.503867065	0.156481575	0.001282009				
			Inverse variance weighted	0.326133036	0.102579109	0.001476149	0.002108785			
			Simple mode	0.503654333	0.355089629	0.159961873				

			Weighted mode	0.669280749	0.223721504	0.00368871				
Pulmonary embolism	BMI-C3	8	MR Egger	1.793188213	1.339232764	0.229076529		-0.021160161	0.02480075	0.426287936
			Weighted median	0.503135209	0.551115149	0.36127396				
			Inverse variance weighted	0.769411413	0.583092314	0.186989918	0.4006927			
			Simple mode	-0.650035822	1.309480222	0.634813414				
			Weighted mode	1.687727405	0.593617497	0.02493281				
Gastro-esophageal reflux disease	BFP-all	384	MR Egger	0.244425669	0.177521425	0.169355772		2.05359E-05	0.002373224	0.993100366
			Weighted median	0.253322284	0.074003826	0.00061912				
			Inverse variance weighted	0.245888573	0.054083076	5.4546E-06	7.1147E-06			
			Simple mode	0.354006005	0.283442133	0.212444947				
			Weighted mode	0.316736625	0.221199204	0.152986082				
Gastro-esophageal reflux disease	BFP-C1	7	MR Egger	0.559079507	0.597711267	0.392538206		0.002702275	0.013675038	0.851135343

			Weighted median	0.504854528	0.21644354	0.01967441				
			Inverse variance weighted	0.666419223	0.228543714	0.003546213	0.007092427			
			Simple mode	0.362974816	0.444114472	0.444990312				
			Weighted mode	0.46787192	0.253264592	0.114206594				
Gastro-esophageal reflux disease	BFP-C2	98	MR Egger	-0.15230389	0.348205139	0.662805867		0.005200003	0.004871538	0.28845671
			Weighted median	0.031900996	0.135370639	0.813699083				
			Inverse variance weighted	0.202558772	0.103639081	0.05064646	0.05843822			
			Simple mode	-0.295537596	0.384021593	0.443415637				
			Weighted mode	-0.189757894	0.310907631	0.543067067				
Gastro-esophageal reflux disease	BFP-C3	14	MR Egger	-2.241536855	0.826643663	0.018898933		0.041114934	0.013910249	0.012015621
			Weighted median	0.261940022	0.242539839	0.280147697				

			Inverse variance weighted	0.152598429	0.194389253	0.432444926	0.5896976			
			Simple mode	0.697312043	0.522628546	0.20502695				
			Weighted mode	0.673902853	0.524918242	0.221614382				
Gastro-esophageal reflux disease	BFP-C4	13	MR Egger	2.678404337	1.040715231	0.025882962		-0.033343828	0.01525256	0.051315052
			Weighted median	0.758491668	0.318192556	0.017137216				
			Inverse variance weighted	0.482558164	0.312282672	0.122283198	0.3668496			
			Simple mode	0.776438629	0.628619642	0.240412327				
			Weighted mode	1.058243245	0.427768026	0.029285062				
Gastro-esophageal reflux disease	BFP-C5	9	MR Egger	1.101154055	0.649361582	0.133752807		-0.020749585	0.01025527	0.08272541
			Weighted median	0.128751077	0.289501208	0.656511878				
			Inverse variance weighted	-0.134487122	0.251037678	0.592148728	0.7105785			
			Simple	-	0.416769608	0.488020129				

			mode	0.302942172						
			Weighted mode	0.102193646	0.33292342	0.766713055				
Gastro-esophageal reflux disease	BMI-all	537	MR Egger	0.07049185	0.09401957	0.45373051		0.001613488	0.00151587	0.287629131
			Weighted median	0.13333649	0.049592638	0.007174487				
			Inverse variance weighted	0.163376812	0.034996309	3.03553E-06	3.64263E-06			
			Simple mode	0.170034215	0.193956108	0.381062238				
			Weighted mode	0.100209811	0.136885839	0.464446721				
Gastro-esophageal reflux disease	BMI-C1	39	MR Egger	0.224779331	0.217887092	0.308941023		0.003383984	0.004495235	0.456337128
			Weighted median	0.343607969	0.14044761	0.014424256				
			Inverse variance weighted	0.369418824	0.102165413	0.000299318	0.000528209			
			Simple mode	0.291182165	0.28372575	0.311248302				
			Weighted mode	0.34720466	0.15716691	0.033266295				
Gastro-esophageal reflux disease	BMI-C2	81	MR	-	0.183069	0.062614452		0.00886875	0.003842456	0.023610519

			Egger	0.345746036						
			Weighted median	-0.112676398	0.100955096	0.264377571				
			Inverse variance weighted	0.038925662	0.077771459	0.616713577	0.6167136			
			Simple mode	-0.059307619	0.208830333	0.777144847				
			Weighted mode	-0.149709599	0.135274339	0.271735657				
Gastro-esophageal reflux disease	BMI-C3	8	MR Egger	0.988832927	0.490177739	0.090237963		-0.009304963	0.009097747	0.345868321
			Weighted median	0.728712285	0.274793127	0.008005026				
			Inverse variance weighted	0.539736667	0.217875347	0.013239067	0.08619338			
			Simple mode	0.149128422	0.444680687	0.747179378				
			Weighted mode	0.700209221	0.28974304	0.04631904				
Rheumatoid arthritis	BFP-all	384	MR Egger	0.427817013	0.239010554	0.074253809		-0.001626625	0.003194516	0.610910867
			Weighted median	0.466659504	0.094776487	8.4884E-07				

			Inverse variance weighted	0.311908836	0.072800183	1.83191E-05	2.28989E-05			
			Simple mode	0.502334423	0.310703242	0.106752123				
			Weighted mode	0.648741757	0.23267741	0.005565181				
Rheumatoid arthritis	BFP-C1	7	MR Egger	0.791380299	0.622633869	0.25964219		0.001969615	0.014250681	0.895466655
			Weighted median	0.890339932	0.313632923	0.004528408				
			Inverse variance weighted	0.8695795	0.252534417	0.000574428	0.001723284			
			Simple mode	0.797551075	0.507835736	0.167355778				
			Weighted mode	0.897458931	0.358832062	0.046461836				
Rheumatoid arthritis	BFP-C2	98	MR Egger	0.219729706	0.414290982	0.597076454		0.001968793	0.00579261	0.734689346
			Weighted median	0.36783798	0.177803642	0.038566017				
			Inverse variance weighted	0.354190377	0.122434235	0.003816991	0.004978683			
			Simple	-	0.45646269	0.689632826				

			mode	0.182835814						
			Weighted mode	0.332300836	0.382769178	0.387455353				
Rheumatoid arthritis	BFP-C3	14	MR Egger	0.927707048	1.764717142	0.608676216		-0.014475466	0.029696454	0.634724596
			Weighted median	0.499495804	0.337230496	0.138561191				
			Inverse variance weighted	0.084841735	0.342068347	0.804114536	0.8636206			
			Simple mode	0.563719843	0.536041684	0.312123525				
			Weighted mode	0.51073173	0.441841609	0.268518033				
Rheumatoid arthritis	BFP-C4	13	MR Egger	-0.508586632	1.279848363	0.698689294		0.005528987	0.018757499	0.773664674
			Weighted median	0.050603835	0.426499221	0.905553204				
			Inverse variance weighted	-0.144495982	0.322083287	0.653699132	0.784439			
			Simple mode	0.560940254	0.87466038	0.533370449				
			Weighted mode	0.780151064	0.892669072	0.399296333				

Rheumatoid arthritis	BFP-C5	9	MR Egger	0.577210792	1.494563856	0.710813829		-0.006891112	0.02358002	0.77857216
			Weighted median	0.331306164	0.441259548	0.452761441				
			Inverse variance weighted	0.166413834	0.477858796	0.727653213	0.8085036			
			Simple mode	0.616805488	0.79655102	0.460996467				
			Weighted mode	0.455082531	0.504504108	0.393383289				
Rheumatoid arthritis	BMI-all	537	MR Egger	0.324284328	0.146196649	0.026964574		-0.001865655	0.00235643	0.428869295
			Weighted median	0.231420085	0.072010634	0.001310396				
			Inverse variance weighted	0.216837785	0.054349595	6.6161E-05	7.63396E-05			
			Simple mode	0.380135303	0.239545485	0.113124471				
			Weighted mode	0.292792893	0.13576674	0.031481825				
Rheumatoid arthritis	BMI-C1	39	MR Egger	0.325084922	0.571746484	0.573074786		0.003379522	0.011785696	0.775905114
			Weighted median	0.550330584	0.190422575	0.003851817				
			Inverse	0.4696531	0.266374374	0.077878222	0.0834409			

			variance weighted	94			5			
			Simple mode	0.52902872	0.403907391	0.198134692				
			Weighted mode	0.426036068	0.199253086	0.038994786				
Rheumatoid arthritis	BMI-C2	81	MR Egger	-0.04060064	0.232704277	0.861940012		0.00731025	0.004881256	0.138217978
			Weighted median	0.182949827	0.144132493	0.204328019				
			Inverse variance weighted	0.276750446	0.096910311	0.004293695	0.005855038			
			Simple mode	-0.168890242	0.310100268	0.58752227				
			Weighted mode	0.037988715	0.18626075	0.838907009				
Rheumatoid arthritis	BMI-C3	8	MR Egger	1.264061066	0.701371882	0.121569739		-0.015068131	0.013006569	0.29068231
			Weighted median	0.738814977	0.395392714	0.061684067				
			Inverse variance weighted	0.536177923	0.317870388	0.091645354	0.3054845			

			Simple mode	0.540514539	0.664940578	0.443053328				
			Weighted mode	0.750636621	0.434688821	0.127837799				
Stroke	BFP-all	384	MR Egger	0.105803887	0.153425896	0.490858766		0.001625999	0.002050795	0.428349798
			Weighted median	0.207759216	0.067984493	0.002243266				
			Inverse variance weighted	0.221657704	0.046755536	2.12897E-06	3.19346E-06			
			Simple mode	0.18261221	0.209088801	0.383007843				
			Weighted mode	0.18261221	0.151794203	0.229709799				
Stroke	BFP-C1	7	MR Egger	-0.020073509	0.390049488	0.960948522		0.012151048	0.008920272	0.231294084
			Weighted median	0.331323403	0.189147161	0.079830572				
			Inverse variance weighted	0.462782239	0.162752222	0.00446242	0.008367037			
			Simple mode	0.302893149	0.295484796	0.344868417				
			Weighted mode	0.313032219	0.1965981	0.162436371				

Stroke	BFP-C2	98	MR Egger	-0.328250773	0.256332529	0.203431387		0.010639752	0.003584452	0.003780811
			Weighted median	0.220937372	0.111717248	0.047968451				
			Inverse variance weighted	0.398269089	0.079160255	4.87483E-07	9.74966E-07			
			Simple mode	0.309109997	0.283716843	0.278631992				
			Weighted mode	0.043954723	0.225865206	0.846108415				
Stroke	BFP-C3	14	MR Egger	0.512376099	0.911406005	0.584340703		-0.003282258	0.015338235	0.834146336
			Weighted median	0.366116298	0.20944279	0.080455511				
			Inverse variance weighted	0.321266587	0.175099809	0.066540867	0.2175296			
			Simple mode	0.720118307	0.376120127	0.077810005				
			Weighted mode	0.548221372	0.344541375	0.135587236				
Stroke	BFP-C4	13	MR Egger	-0.42267269	0.774420667	0.596103632		0.005414295	0.011356364	0.642861404
			Weighted	-0.1101171	0.253844028	0.664434845				

			median	33						
			Inverse variance weighted	-0.066352023	0.196234842	0.73526854	0.8483868			
			Simple mode	-0.19734477	0.38852614	0.620708089				
			Weighted mode	-0.142710387	0.340072333	0.68215846				
Stroke	BFP-C5	9	MR Egger	0.184066873	0.764890865	0.816726545		-0.014028185	0.012078451	0.283537777
			Weighted median	-0.447886547	0.269676999	0.096748416				
			Inverse variance weighted	-0.65148014	0.265407502	0.014102534	0.03254431			
			Simple mode	-0.486153983	0.405546423	0.264921744				
			Weighted mode	-0.429430428	0.274490598	0.15633889				
Stroke	BMI-all	537	MR Egger	0.141721193	0.087154839	0.104520382		0.001543821	0.001404884	0.272307964
			Weighted median	0.144731732	0.049862146	0.003700348				
			Inverse variance	0.230622883	0.032425423	1.14034E-12	1.90057E-12			

			weighted							
			Simple mode	0.086807911	0.145280818	0.5504139				
			Weighted mode	0.086807911	0.086675217	0.317022109				
Stroke	BMI-C1	39	MR Egger	0.112709043	0.193388619	0.563555831		0.006152837	0.003987083	0.131294676
			Weighted median	0.154219135	0.119767945	0.197867999				
			Inverse variance weighted	0.375904307	0.09280713	5.11341E-05	0.000139457			
			Simple mode	0.206176339	0.222397971	0.359746224				
			Weighted mode	0.174643052	0.116730504	0.142881902				
Stroke	BMI-C2	81	MR Egger	-0.070346587	0.169394843	0.679061412		0.005472731	0.00355394	0.127579611
			Weighted median	0.099960955	0.089118971	0.26200813				
			Inverse variance weighted	0.167164278	0.0706367	0.017955439	0.0207178 1			
			Simple mode	0.198610764	0.191207755	0.302065619				
			Weight	0.0453355	0.120123927	0.706871128				

			ed mode	16						
Stroke	BMI-C3	8	MR Egger	-0.082672533	0.450798712	0.860531461		-0.00240781	0.008369268	0.783257606
			Weighted median	-0.076433281	0.241767855	0.751893761				
			Inverse variance weighted	-0.198842566	0.200425097	0.321146734	0.4817201			
			Simple mode	-0.665034361	0.39520078	0.136296879				
			Weighted mode	0.012320021	0.28847108	0.967126996				
Type 2 diabetes	BFP-all	384	MR Egger	0.935120992	0.251244828	0.000227405		-0.002053961	0.003358916	0.541235504
			Weighted median	0.818376451	0.064552784	7.86727E-37				
			Inverse variance weighted	0.788815208	0.076612324	7.33059E-25	5.49794E-24			
			Simple mode	1.05167444	0.261140391	6.80616E-05				
			Weighted mode	0.929241336	0.21245584	1.57581E-05				
Type 2 diabetes	BFP-C1	7	MR Egger	2.946084654	0.595781149	0.004303794		-0.0133571	0.013651621	0.372798727

			Weighted median	2.378634029	0.217018195	5.91531E-28				
			Inverse variance weighted	2.416313183	0.247678245	1.74126E-22	5.22379E-21			
			Simple mode	1.722808166	0.648112872	0.037614749				
			Weighted mode	2.766238028	0.226275747	1.82347E-05				
Type 2 diabetes	BFP-C2	98	MR Egger	1.669528639	0.296445123	1.77264E-07		-0.002677218	0.004147196	0.520111494
			Weighted median	1.428859334	0.108779562	2.06491E-39				
			Inverse variance weighted	1.486812784	0.087872866	3.2019E-64	9.6057E-63			
			Simple mode	1.567964414	0.29496645	6.78949E-07				
			Weighted mode	1.567964414	0.324210891	4.9718E-06				
Type 2 diabetes	BFP-C3	14	MR Egger	0.35365106	0.731109852	0.63729278		-0.000182061	0.01230445	0.988437838
			Weighted median	0.311118966	0.188085474	0.09809987				
			Inverse variance	0.343051106	0.140236973	0.014436111	0.08661667			

			weighted							
			Simple mode	0.497591815	0.365800997	0.19686272				
			Weighted mode	0.698498559	0.356247745	0.071697895				
Type 2 diabetes	BFP-C4	13	MR Egger	0.752866619	0.797176974	0.365239258		-0.030257256	0.01168483	0.025162977
			Weighted median	-1.439233184	0.237981401	1.46955E-09				
			Inverse variance weighted	-1.239338517	0.253584252	1.02236E-06	3.06708E-05			
			Simple mode	-1.742218294	0.419989495	0.001350813				
			Weighted mode	-1.612499678	0.376214578	0.001057514				
Type 2 diabetes	BFP-C5	9	MR Egger	-2.377896108	0.793321158	0.020015783		-0.011353794	0.012485637	0.393398308
			Weighted median	-3.012679761	0.282014416	1.22631E-26				
			Inverse variance weighted	-3.05672331	0.265591005	1.1866E-30	3.55979E-29			
			Simple mode	-3.1384001	0.494121961	0.000220263				

				7						
			Weighted mode	-2.895531867	0.38699688	7.04658E-05				
Type 2 diabetes	BMI-all	537	MR Egger	1.078811231	0.100999856	2.84682E-24		-0.003868017	0.001628356	0.017880752
			Weighted median	0.87930899	0.042069632	5.21325E-97				
			Inverse variance weighted	0.856128362	0.037750404	7.2781E-114	2.1834E-112			
			Simple mode	0.819709074	0.167521747	1.31455E-06				
			Weighted mode	0.88703584	0.136622411	1.92549E-10				
Type 2 diabetes	BMI-C1	39	MR Egger	1.285029101	0.196057357	1.12023E-07		0.003664604	0.004042881	0.370571781
			Weighted median	1.515595341	0.110092683	4.05157E-43				
			Inverse variance weighted	1.441753119	0.092213627	4.20972E-55	1.26292E-53			
			Simple mode	1.540696221	0.277963678	2.41238E-06				
			Weighted mode	1.464322214	0.133620971	2.52331E-13				
Type 2 diabetes	BMI-C2	81	MR Egger	1.00898014	0.147731259	1.57786E-09		-0.003034806	0.003101401	0.330800741

			Weighted median	0.956713265	0.072312068	5.86214E-40				
			Inverse variance weighted	0.877394072	0.061150563	1.09504E-46	3.28513E-45			
			Simple mode	0.929765934	0.155680548	6.14331E-08				
			Weighted mode	0.961246398	0.093405447	2.62464E-16				
Type 2 diabetes	BMI-C3	8	MR Egger	0.486076775	0.659366305	0.488821492		-0.025585916	0.012235313	0.081468219
			Weighted median	-0.742108595	0.323852598	0.021934446				
			Inverse variance weighted	-0.749094943	0.356733297	0.035739934	0.1531711			
			Simple mode	-1.055581097	0.479972796	0.063801428				
			Weighted mode	0.152480699	0.274295601	0.595592738				
Venous thromboembolism	BFP-all	384	MR Egger	0.763808779	0.210175053	0.000317035		-0.003689104	0.002809462	0.18993755
			Weighted median	0.555020472	0.088223793	3.15322E-10				
			Inverse varianc	0.500969876	0.064145111	5.72124E-15	2.14546E-14			

			e weighted							
			Simple mode	0.288437143	0.291400828	0.322882366				
			Weighted mode	0.546126623	0.187555666	0.003803681				
Venous thromboembolism	BFP-C1	7	MR Egger	0.899251143	0.485731614	0.123337124		-0.011052949	0.011110888	0.365514537
			Weighted median	0.543120827	0.244090058	0.026075615				
			Inverse variance weighted	0.460127629	0.202679146	0.023193825	0.03457335			
			Simple mode	0.486852289	0.334017914	0.195228764				
			Weighted mode	0.552265225	0.267002704	0.08407476				
Venous thromboembolism	BFP-C2	98	MR Egger	0.456224927	0.386721335	0.241024728		0.000817156	0.005408659	0.880227286
			Weighted median	0.547234582	0.144369491	0.000150336				
			Inverse variance weighted	0.51201217	0.114348025	7.54638E-06	1.41495E-05			
			Simple mode	0.45781351	0.397895665	0.252730808				
			Weight	0.6217848	0.320521978	0.055294711				

			Weighted mode	92						
Venous thromboembolism	BFP-C3	14	MR Egger	-0.645568032	1.036276029	0.54496259		0.014667523	0.017440487	0.416789512
			Weighted median	0.283910871	0.279015156	0.308893565				
			Inverse variance weighted	0.208402929	0.204524535	0.308220544	0.5439186			
			Simple mode	0.454783065	0.527516843	0.404244358				
			Weighted mode	0.393433874	0.450000012	0.397820672				
Venous thromboembolism	BFP-C4	13	MR Egger	3.807568702	1.613331776	0.037809498		-0.043842064	0.023649561	0.090752716
			Weighted median	1.565780947	0.361089117	1.44919E-05				
			Inverse variance weighted	0.920839841	0.462806168	0.046625324	0.1998228			
			Simple mode	0.904221137	0.748168702	0.250101222				
			Weighted mode	1.535149749	0.396381646	0.002216171				
Venous thromboembolism	BFP-C5	9	MR Egger	1.353101189	1.141484567	0.274540296		-0.006723597	0.018011083	0.719970987
			Weight	1.1404663	0.347390722	0.001027269				

			ed median	87						
			Inverse variance weighted	0.952276393	0.365999028	0.009272105	0.02753962			
			Simple mode	1.159468363	0.506851347	0.05145802				
			Weighted mode	1.098225735	0.392186675	0.023188447				
Venous thromboembolism	BMI-all	537	MR Egger	0.494864955	0.104975425	3.10048E-06		-0.002640402	0.001692187	0.119268686
			Weighted median	0.369453419	0.060256579	8.71433E-10				
			Inverse variance weighted	0.342818447	0.039097415	1.8136E-18	4.534E-18			
			Simple mode	0.510613441	0.18564486	0.006151831				
			Weighted mode	0.394085543	0.116550363	0.000774204				
Venous thromboembolism	BMI-C1	39	MR Egger	0.183708245	0.272455055	0.504330962		0.004312542	0.005617586	0.447545465
			Weighted median	0.271183259	0.150793158	0.072116958				
			Inverse variance weight	0.368171517	0.12773692	0.003948289	0.005922433			

			ed							
			Simple mode	0.636086315	0.295517973	0.037776776				
			Weighted mode	0.289030032	0.143846653	0.051647786				
Venous thromboembolism	BMI-C2	81	MR Egger	0.495638577	0.19900198	0.01484932		-0.001614557	0.004174917	0.699997448
			Weighted median	0.380711271	0.115240956	0.000954482				
			Inverse variance weighted	0.425564048	0.081834666	1.98977E-07	6.63257E-07			
			Simple mode	0.422328151	0.24533258	0.089033922				
			Weighted mode	0.452773045	0.163797887	0.007079954				
Venous thromboembolism	BMI-C3	8	MR Egger	1.859487745	1.079023675	0.135603615		-0.030567156	0.019996017	0.177213392
			Weighted median	0.675933468	0.38012003	0.075369272				
			Inverse variance weighted	0.381616352	0.522966852	0.465564883	0.6348612			
			Simple mode	-0.722103224	0.946363382	0.470373567				
			Weighted mode	0.863826526	0.364604033	0.049663285				

Table S6: Correlated and shared variants between body fat percentage and BMI - increasing clusters. Correlation between adiposity increasing cluster variants were investigated using $r^2 \geq 0.8$. There was no correlation between unfavorable adiposity clusters (BFP-C1, BFP-C2, BFP-C3, BMI-C1, BMI-C2) and favorable adiposity clusters (BFP-C4, BFP-C5, BMI-C3). c – correlated variants, s – shared variants.

	**BFP-C1**	**BFP-C2**	**BFP-C3**	**BFP-C4**	**BFP-C5**	**Total n cluster variants**
**BMI-C1**	3c, 1s	4c				**39**
**BMI-C2**		5c, 4s	2c			**82**
**BMI-C3**				1c		**8**
**Total n cluster variants**	**7**	**101**	**14**	**13**	**9**	

Table S7: F-statistics for each BFP/BMI-increasing cluster. The F-statistics for each BFP/BMI-increasing cluster were calculated based on the beta and se of their respective GWAS. All F-statistics were > 50, where F > 10 indicates a strong instrument for Mendelian Randomization (MR).

**Cluster**	**F-statistic**
BFP-all	58
BFP-C1	177
BFP-C2	69
BFP-C3	107
BFP-C4	71
BFP-C5	87
BMI-all	72
BMI-C1	120
BMI-C2	121
BMI-C3	83

Table S8: eQTL analysis results. Shown are the number of independent eQTLs identified by FUMA, that were found in subcutaneous adipose, visceral (omentum) adipose and brain tissue within each adiposity cluster. Data sources used for tissues were MuTHER and GTEx version 8.

**Tissue**	**BFP-C1**	**BFP-C2**	**BFP-C3**	**BFP-C4**	**BFP-C5**	**BMI-C1**	**BMI-C2**	**BMI-C3**
Subcutaneous adipose	3	13	10	10	5	13	10	4
Visceral (omentum) adipose	2	13	9	6	2	13	6	4
Brain	2	35	9	5	4	14	20	5

Table S9: Pathway analysis results for all adiposity-increasing clusters. The PANTHER Overrepresentation Test was performed using PANTHER pathways. The reference gene list was comprised of 20,589 genes from Homo sapiens. The number of genes input for each adiposity-increasing cluster were as follows: BFP-C1: 34, BFP-C2: 597, BFP-C3: 107, BFP-C4: 105, BFP-C5: 37, BMI-C1: 188, BMI-C2: 478, BMI-C3: 114. Expected number of genes was calculated by multiplying the percentage of mapped reference genes by the number of genes input for each cluster. Fold enrichment was calculated by dividing the number of mapped input genes by the expected number. pval - probability of input genes occurring by chance based on the reference list, determined by Fischer's exact test. BHp – Benjamini-Hochberg corrected p-value to account for false discovery rate.

Cluster	PANTHER Pathways	Mapped reference genes	Mapped input genes	Expected number of genes	Over/Underrepresented	Fold Enrichment	pval	BHp
BFP-C1	Cholesterol biosynthesis (P00014)	13	1	0.02	+	46.58	2.28E-02	1.00E+00
	General transcription by RNA polymerase I (P00022)	16	1	0.03	+	37.85	2.77E-02	1.00E+00
	p53 pathway by glucose deprivation (P04397)	23	1	0.04	+	26.33	3.88E-02	1.00E+00
	Endogenous cannabinoid signaling (P05730)	24	1	0.04	+	25.23	4.04E-02	1.00E+00
	Histamine H2 receptor mediated signaling pathway (P04386)	27	1	0.04	+	22.43	4.52E-02	1.00E+00
	Beta3 adrenergic receptor signaling pathway (P04379)	29	1	0.05	+	20.88	4.83E-02	9.67E-01
	Cortocotropin releasing factor receptor signaling pathway (P04380)	33	1	0.05	+	18.35	5.46E-02	9.71E-01
	5HT4 type receptor mediated signaling pathway (P04376)	34	1	0.06	+	17.81	5.62E-02	8.99E-01
	Opioid prodynorphin pathway (P05916)	35	1	0.06	+	17.3	5.77E-02	8.40E-01
	GABA-B receptor II signaling (P05731)	36	1	0.06	+	16.82	5.93E-02	7.90E-01
	Opioid proopiomelanocortin pathway (P05917)	36	1	0.06	+	16.82	5.93E-02	7.30E-01
	Opioid proenkephalin pathway (P05915)	37	1	0.06	+	16.37	6.08E-02	6.95E-01

	Angiotensin II-stimulated signaling through G proteins and beta-arrestin (P05911)	37	1	0.06	+	16.37	6.08E-02	6.49E-01
	Heterotrimeric G-protein signaling pathway-rod outer segment phototransduction (P00028)	37	1	0.06	+	16.37	6.08E-02	6.08E-01
	Enkephalin release (P05913)	38	1	0.06	+	15.94	6.24E-02	5.87E-01
	Cytoskeletal regulation by Rho GTPase (P00016)	86	2	0.14	+	14.08	9.24E-03	1.00E+00
	Histamine H1 receptor mediated signaling pathway (P04385)	45	1	0.07	+	13.46	7.32E-02	6.16E-01
	Blood coagulation (P00011)	46	1	0.08	+	13.16	7.47E-02	5.98E-01
	Beta2 adrenergic receptor signaling pathway (P04378)	47	1	0.08	+	12.88	7.62E-02	5.81E-01
	Beta1 adrenergic receptor signaling pathway (P04377)	47	1	0.08	+	12.88	7.62E-02	5.54E-01
	Metabotropic glutamate receptor group II pathway (P00040)	48	1	0.08	+	12.62	7.78E-02	5.41E-01
	5HT1 type receptor mediated signaling pathway (P04373)	48	1	0.08	+	12.62	7.78E-02	5.18E-01
	PI3 kinase pathway (P00048)	57	1	0.09	+	10.62	9.14E-02	5.85E-01
	Ubiquitin proteasome pathway (P00060)	58	1	0.1	+	10.44	9.29E-02	5.72E-01
	Oxytocin receptor mediated signaling pathway (P04391)	59	1	0.1	+	10.26	9.44E-02	5.59E-01
	Muscarinic acetylcholine receptor 1 and 3 signaling pathway (P00042)	59	1	0.1	+	10.26	9.44E-02	5.39E-01
	Thyrotropin-releasing hormone receptor signaling pathway (P04394)	61	1	0.1	+	9.93	9.74E-02	5.37E-01
	Muscarinic acetylcholine receptor 2 and 4 signaling pathway (P00043)	61	1	0.1	+	9.93	9.74E-02	5.19E-01
	5HT2 type receptor mediated signaling	67	1	0.11	+	9.04	1.06E-01	5.49E-01

	pathway (P04374)							
	Metabotropic glutamate receptor group III pathway (P00039)	70	1	0.12	+	8.65	1.11E-01	5.54E-01
	Huntington disease (P00029)	152	2	0.25	+	7.97	2.66E-02	1.00E+00
	Heterotrimeric G-protein signaling pathway-Gq alpha and Go alpha mediated pathway (P00027)	125	1	0.21	+	4.84	1.88E-01	9.13E-01
	Heterotrimeric G-protein signaling pathway-Gi alpha and Gs alpha mediated pathway (P00026)	163	1	0.27	+	3.72	2.38E-01	1.00E+00
BFP-C2	Tetrahydrofolate biosynthesis (P02742)	5	2	0.14	+	13.79	1.52E-02	2.69E-01
	5-Hydroxytryptamine biosynthesis (P04371)	3	1	0.09	+	11.5	1.08E-01	7.52E-01
	JAK/STAT signaling pathway (P00038)	17	4	0.49	+	8.11	2.55E-03	4.08E-01
	Interferon-gamma signaling pathway (P00035)	30	5	0.87	+	5.75	2.82E-03	2.25E-01
	Mannose metabolism (P02752)	6	1	0.17	+	5.75	1.81E-01	1.00E+00
	p53 pathway by glucose deprivation (P04397)	23	3	0.67	+	4.5	3.58E-02	4.09E-01
	Formyltetrahydrofolate biosynthesis (P02743)	8	1	0.23	+	4.31	2.27E-01	1.00E+00
	p38 MAPK pathway (P05918)	41	5	1.19	+	4.21	9.24E-03	1.85E-01
	Insulin/IGF pathway-mitogen activated protein kinase kinase/MAP kinase cascade (P00032)	33	4	0.96	+	4.18	1.98E-02	3.16E-01
	Pentose phosphate pathway (P02762)	9	1	0.26	+	3.83	2.49E-01	1.00E+00
	Ubiquitin proteasome pathway (P00060)	58	6	1.68	+	3.57	9.19E-03	2.10E-01
	Ras Pathway (P04393)	76	7	2.2	+	3.18	8.93E-03	2.38E-01
	Fructose galactose metabolism (P02744)	11	1	0.32	+	3.14	2.90E-01	1.00E+00
	Interleukin signaling pathway (P00036)	89	8	2.58	+	3.1	6.10E-03	2.44E-01
	Oxidative stress response (P00046)	56	5	1.62	+	3.08	2.85E-02	3.80E-01

	Cell cycle (P00013)	23	2	0.67	+	3	1.56E-01	1.00E+00
	p53 pathway feedback loops 2 (P04398)	51	4	1.48	+	2.7	6.91E-02	5.52E-01
	EGF receptor signaling pathway (P00018)	142	11	4.12	+	2.67	4.13E-03	2.20E-01
	Salvage pyrimidine ribonucleotides (P02775)	13	1	0.38	+	2.65	3.30E-01	1.00E+00
	Heme biosynthesis (P02746)	13	1	0.38	+	2.65	3.30E-01	1.00E+00
	Cholesterol biosynthesis (P00014)	13	1	0.38	+	2.65	3.30E-01	1.00E+00
	VEGF signaling pathway (P00056)	68	5	1.97	+	2.54	5.50E-02	4.63E-01
	De novo pyrimidine ribonucleotides biosythesis (P02740)	14	1	0.41	+	2.46	3.49E-01	1.00E+00
	PI3 kinase pathway (P00048)	57	4	1.65	+	2.42	9.30E-02	7.08E-01
	Hypoxia response via HIF activation (P00030)	29	2	0.84	+	2.38	2.17E-01	1.00E+00
	DNA replication (P00017)	29	2	0.84	+	2.38	2.17E-01	1.00E+00
	p53 pathway (P00059)	89	6	2.58	+	2.32	5.19E-02	4.88E-01
	General transcription by RNA polymerase I (P00022)	16	1	0.46	+	2.16	3.85E-01	1.00E+00
	Metabotropic glutamate receptor group II pathway (P00040)	48	3	1.39	+	2.16	1.73E-01	1.00E+00
	Inflammation mediated by chemokine and cytokine signaling pathway (P00031)	261	16	7.57	+	2.11	8.71E-03	2.79E-01
	Angiogenesis (P00005)	181	11	5.25	+	2.1	2.46E-02	3.58E-01
	CCKR signaling map (P06959)	173	10	5.02	+	1.99	4.02E-02	4.02E-01
	Opioid prodynorphin pathway (P05916)	35	2	1.01	+	1.97	2.80E-01	1.00E+00
	Opioid proopiomelanocortin pathway (P05917)	36	2	1.04	+	1.92	2.91E-01	1.00E+00
	Integrin signalling pathway (P00034)	200	11	5.8	+	1.9	5.41E-02	4.81E-01
	Opioid proenkephalin pathway (P05915)	37	2	1.07	+	1.86	3.01E-01	1.00E+00
	Angiotensin II-stimulated signaling	37	2	1.07	+	1.86	3.01E-01	1.00E+00

	through G proteins and beta-arrestin (P05911)							
	Enkephalin release (P05913)	38	2	1.1	+	1.82	3.12E-01	1.00E+00
	Dopamine receptor mediated signaling pathway (P05912)	58	3	1.68	+	1.78	2.47E-01	1.00E+00
	Insulin/IGF pathway-protein kinase B signaling cascade (P00033)	40	2	1.16	+	1.72	3.32E-01	1.00E+00
	Glycolysis (P00024)	20	1	0.58	+	1.72	4.51E-01	1.00E+00
	Muscarinic acetylcholine receptor 2 and 4 signaling pathway (P00043)	61	3	1.77	+	1.7	4.30E-01	1.00E+00
	PDGF signaling pathway (P00047)	145	7	4.2	+	1.66	2.08E-01	1.00E+00
	Axon guidance mediated by semaphorins (P00007)	21	1	0.61	+	1.64	4.67E-01	1.00E+00
	FGF signaling pathway (P00021)	127	6	3.68	+	1.63	2.81E-01	1.00E+00
	Endothelin signaling pathway (P00019)	85	4	2.46	+	1.62	3.20E-01	1.00E+00
	Cytoskeletal regulation by Rho GTPase (P00016)	86	4	2.49	+	1.6	3.24E-01	1.00E+00
	Huntington disease (P00029)	152	7	4.41	+	1.59	2.22E-01	1.00E+00
	Alzheimer disease-amyloid secretase pathway (P00003)	67	3	1.94	+	1.54	4.51E-01	1.00E+00
	T cell activation (P00053)	92	4	2.67	+	1.5	3.50E-01	1.00E+00
	Blood coagulation (P00011)	46	2	1.33	+	1.5	3.94E-01	1.00E+00
	5HT1 type receptor mediated signaling pathway (P04373)	48	2	1.39	+	1.44	6.53E-01	1.00E+00
	Apoptosis signaling pathway (P00006)	125	5	3.62	+	1.38	4.20E-01	1.00E+00
	Axon guidance mediated by Slit/Robo (P00008)	25	1	0.72	+	1.38	5.25E-01	1.00E+00
	TGF-beta signaling pathway (P00052)	102	4	2.96	+	1.35	5.46E-01	1.00E+00
	Gonadotropin-releasing hormone receptor pathway (P06664)	237	9	6.87	+	1.31	4.33E-01	1.00E+00
	Adrenaline and noradrenaline biosynthesis (P00001)	29	1	0.84	+	1.19	5.76E-01	1.00E+00

	Transcription regulation by bZIP transcription factor (P00055)	60	2	1.74	+	1.15	6.94E-01	1.00E+00
	Toll receptor signaling pathway (P00054)	61	2	1.77	+	1.13	6.98E-01	1.00E+00
	De novo purine biosynthesis (P02738)	31	1	0.9	+	1.11	6.00E-01	1.00E+00
	Parkinson disease (P00049)	101	3	2.93	+	1.02	7.69E-01	1.00E+00
	FAS signaling pathway (P00020)	34	1	0.99	+	1.01	1.00E+00	1.00E+00
	Nicotine pharmacodynamics pathway (P06587)	35	1	1.01	-	0.99	1.00E+00	1.00E+00
	Metabotropic glutamate receptor group III pathway (P00039)	70	2	2.03	-	0.99	1.00E+00	1.00E+00
	GABA-B receptor II signaling (P05731)	36	1	1.04	-	0.96	1.00E+00	1.00E+00
	Heterotrimeric G-protein signaling pathway-rod outer segment phototransduction (P00028)	37	1	1.07	-	0.93	1.00E+00	1.00E+00
	General transcription regulation (P00023)	40	1	1.16	-	0.86	1.00E+00	1.00E+00
	Heterotrimeric G-protein signaling pathway-Gi alpha and Gs alpha mediated pathway (P00026)	163	4	4.73	-	0.85	1.00E+00	1.00E+00
	Alzheimer disease-presenilin pathway (P00004)	131	3	3.8	-	0.79	1.00E+00	1.00E+00
	Ionotropic glutamate receptor pathway (P00037)	49	1	1.42	-	0.7	1.00E+00	1.00E+00
	Nicotinic acetylcholine receptor signaling pathway (P00044)	110	2	3.19	-	0.63	7.73E-01	1.00E+00
	Heterotrimeric G-protein signaling pathway-Gq alpha and Go alpha mediated pathway (P00027)	125	2	3.62	-	0.55	5.90E-01	1.00E+00
	B cell activation (P00010)	71	1	2.06	-	0.49	7.25E-01	1.00E+00
	Wnt signaling pathway (P00057)	314	3	9.1	-	0.33	3.87E-02	4.13E-01
	Cadherin signaling pathway (P00012)	166	1	4.81	-	0.21	9.61E-02	6.99E-01
BFP-C3	Alanine biosynthesis (P02724)	2	1	0.01	+	96.21	1.54E-02	6.17E-01

	Valine biosynthesis (P02785)	3	1	0.02	+	64.14	2.05E-02	4.69E-01
	Leucine biosynthesis (P02749)	3	1	0.02	+	64.14	2.05E-02	4.10E-01
	O-antigen biosynthesis (P02757)	4	1	0.02	+	48.11	2.56E-02	4.55E-01
	Isoleucine biosynthesis (P02748)	4	1	0.02	+	48.11	2.56E-02	4.09E-01
	N-acetylglucosamine metabolism (P02756)	7	1	0.04	+	27.49	4.06E-02	5.91E-01
	JAK/STAT signaling pathway (P00038)	17	2	0.09	+	22.64	4.28E-03	3.42E-01
	5-Hydroxytryptamine degredation (P04372)	21	2	0.11	+	18.33	6.24E-03	3.33E-01
	Endothelin signaling pathway (P00019)	85	5	0.44	+	11.32	1.04E-04	1.67E-02
	GABA-B receptor II signaling (P05731)	36	2	0.19	+	10.69	1.65E-02	5.28E-01
	Hypoxia response via HIF activation (P00030)	29	1	0.15	+	6.64	1.44E-01	1.00E+00
	Interferon-gamma signaling pathway (P00035)	30	1	0.16	+	6.41	1.49E-01	1.00E+00
	Synaptic vesicle trafficking (P05734)	31	1	0.16	+	6.21	1.53E-01	1.00E+00
	Cortocotropin releasing factor receptor signaling pathway (P04380)	33	1	0.17	+	5.83	1.62E-01	1.00E+00
	Insulin/IGF pathway-mitogen activated protein kinase kinase/MAP kinase cascade (P00032)	33	1	0.17	+	5.83	1.62E-01	1.00E+00
	VEGF signaling pathway (P00056)	68	2	0.35	+	5.66	5.09E-02	5.82E-01
	Axon guidance mediated by netrin (P00009)	35	1	0.18	+	5.5	1.70E-01	1.00E+00
	Opioid proopiomelanocortin pathway (P05917)	36	1	0.19	+	5.35	1.75E-01	1.00E+00
	Angiotensin II-stimulated signaling through G proteins and beta-arrestin (P05911)	37	1	0.19	+	5.2	1.79E-01	1.00E+00
	Insulin/IGF pathway-protein kinase B signaling cascade (P00033)	40	1	0.21	+	4.81	1.92E-01	1.00E+00
	Interleukin signaling pathway (P00036)	89	2	0.46	+	4.32	8.06E-02	8.06E-01

	T cell activation (P00053)	92	2	0.48	+	4.18	8.52E-02	8.02E-01
	PDGF signaling pathway (P00047)	145	3	0.75	+	3.98	4.15E-02	5.53E-01
	Huntington disease (P00029)	152	3	0.79	+	3.8	4.65E-02	5.73E-01
	p53 pathway feedback loops 2 (P04398)	51	1	0.27	+	3.77	2.37E-01	1.00E+00
	Heterotrimeric G-protein signaling pathway-Gi alpha and Gs alpha mediated pathway (P00026)	163	3	0.85	+	3.54	5.50E-02	5.86E-01
	Oxidative stress response (P00046)	56	1	0.29	+	3.44	2.56E-01	1.00E+00
	PI3 kinase pathway (P00048)	57	1	0.3	+	3.38	2.60E-01	1.00E+00
	Toll receptor signaling pathway (P00054)	61	1	0.32	+	3.15	2.75E-01	1.00E+00
	Heterotrimeric G-protein signaling pathway-Gq alpha and Go alpha mediated pathway (P00027)	125	2	0.65	+	3.08	1.40E-01	1.00E+00
	Integrin signalling pathway (P00034)	200	3	1.04	+	2.89	8.82E-02	7.84E-01
	EGF receptor signaling pathway (P00018)	142	2	0.74	+	2.71	1.71E-01	1.00E+00
	B cell activation (P00010)	71	1	0.37	+	2.71	3.12E-01	1.00E+00
	Ras Pathway (P04393)	76	1	0.39	+	2.53	3.30E-01	1.00E+00
	p53 pathway (P00059)	89	1	0.46	+	2.16	3.73E-01	1.00E+00
	Angiogenesis (P00005)	181	2	0.94	+	2.13	2.44E-01	1.00E+00
	TGF-beta signaling pathway (P00052)	102	1	0.53	+	1.89	4.14E-01	1.00E+00
	Gonadotropin-releasing hormone receptor pathway (P06664)	237	2	1.23	+	1.62	3.51E-01	1.00E+00
	Apoptosis signaling pathway (P00006)	125	1	0.65	+	1.54	4.81E-01	1.00E+00
	FGF signaling pathway (P00021)	127	1	0.66	+	1.52	4.86E-01	1.00E+00
	Inflammation mediated by chemokine and cytokine signaling pathway (P00031)	261	2	1.36	+	1.47	3.95E-01	1.00E+00
	CCKR signaling map (P06959)	173	1	0.9	+	1.11	5.96E-01	1.00E+00
BFP-C4	Circadian clock system (P00015)	10	1	0.05	+	19.61	5.44E-02	1.00E+00
	GABA-B receptor II signaling (P05731)	36	2	0.18	+	10.89	1.59E-02	1.00E+00

	Hedgehog signaling pathway (P00025)	20	1	0.1	+	9.8	1.01E-01	1.00E+00
	Alzheimer disease-amyloid secretase pathway (P00003)	67	3	0.34	+	8.78	5.43E-03	8.69E-01
	Endogenous cannabinoid signaling (P05730)	24	1	0.12	+	8.17	1.19E-01	1.00E+00
	Axon guidance mediated by netrin (P00009)	35	1	0.18	+	5.6	1.67E-01	1.00E+00
	Nicotinic acetylcholine receptor signaling pathway (P00044)	110	3	0.56	+	5.35	1.99E-02	1.00E+00
	Angiotensin II-stimulated signaling through G proteins and beta-arrestin (P05911)	37	1	0.19	+	5.3	1.76E-01	1.00E+00
	General transcription regulation (P00023)	40	1	0.2	+	4.9	1.88E-01	1.00E+00
	Metabotropic glutamate receptor group II pathway (P00040)	48	1	0.24	+	4.09	2.21E-01	1.00E+00
	5HT1 type receptor mediated signaling pathway (P04373)	48	1	0.24	+	4.09	2.21E-01	1.00E+00
	Ionotropic glutamate receptor pathway (P00037)	49	1	0.25	+	4	2.25E-01	1.00E+00
	Dopamine receptor mediated signaling pathway (P05912)	58	1	0.3	+	3.38	2.60E-01	1.00E+00
	Muscarinic acetylcholine receptor 1 and 3 signaling pathway (P00042)	59	1	0.3	+	3.32	2.63E-01	1.00E+00
	Transcription regulation by bZIP transcription factor (P00055)	60	1	0.31	+	3.27	2.67E-01	1.00E+00
	Muscarinic acetylcholine receptor 2 and 4 signaling pathway (P00043)	61	1	0.31	+	3.21	2.71E-01	1.00E+00
	5HT2 type receptor mediated signaling pathway (P04374)	67	1	0.34	+	2.93	2.93E-01	1.00E+00
	Huntington disease (P00029)	152	2	0.78	+	2.58	1.84E-01	1.00E+00
	Endothelin signaling pathway (P00019)	85	1	0.43	+	2.31	3.55E-01	1.00E+00

	Cytoskeletal regulation by Rho GTPase (P00016)	86	1	0.44	+	2.28	3.58E-01	1.00E+00
	Inflammation mediated by chemokine and cytokine signaling pathway (P00031)	261	3	1.33	+	2.25	1.51E-01	1.00E+00
	Parkinson disease (P00049)	101	1	0.52	+	1.94	4.06E-01	1.00E+00
	TGF-beta signaling pathway (P00052)	102	1	0.52	+	1.92	4.09E-01	1.00E+00
	Heterotrimeric G-protein signaling pathway-Gi alpha and Gs alpha mediated pathway (P00026)	163	1	0.83	+	1.2	5.67E-01	1.00E+00
	CCKR signaling map (P06959)	173	1	0.88	+	1.13	5.89E-01	1.00E+00
BFP-C5	Phenylethylamine degradation (P02766)	5	1	0.01	+	> 100	1.07E-02	1.00E+00
	PI3 kinase pathway (P00048)	57	1	0.1	+	9.76	9.90E-02	1.00E+00
	VEGF signaling pathway (P00056)	68	1	0.12	+	8.18	1.17E-01	1.00E+00
	Endothelin signaling pathway (P00019)	85	1	0.15	+	6.55	1.43E-01	1.00E+00
	Interleukin signaling pathway (P00036)	89	1	0.16	+	6.25	1.50E-01	1.00E+00
	Angiogenesis (P00005)	181	2	0.33	+	6.15	4.26E-02	1.00E+00
	CCKR signaling map (P06959)	173	1	0.31	+	3.22	2.69E-01	1.00E+00
	Gonadotropin-releasing hormone receptor pathway (P06664)	237	1	0.43	+	2.35	3.49E-01	1.00E+00
BMI-C1	Succinate to proprionate conversion (P02777)	5	1	0.05	+	21.9	5.31E-02	1.00E+00
	Methylmalonyl pathway (P02755)	6	1	0.05	+	18.25	6.17E-02	1.00E+00
	Mannose metabolism (P02752)	6	1	0.05	+	18.25	6.17E-02	1.00E+00
	Cholesterol biosynthesis (P00014)	13	2	0.12	+	16.85	7.91E-03	6.33E-01
	N-acetylglucosamine metabolism (P02756)	7	1	0.06	+	15.65	7.01E-02	1.00E+00
	TCA cycle (P00051)	11	1	0.1	+	9.96	1.03E-01	1.00E+00
	Heme biosynthesis (P02746)	13	1	0.12	+	8.42	1.20E-01	1.00E+00
	Ubiquitin proteasome pathway (P00060)	58	4	0.53	+	7.55	2.41E-03	3.85E-01

	General transcription by RNA polymerase I (P00022)	16	1	0.15	+	6.84	1.43E-01	1.00E+00
	p53 pathway by glucose deprivation (P04397)	23	1	0.21	+	4.76	1.96E-01	1.00E+00
	Cell cycle (P00013)	23	1	0.21	+	4.76	1.96E-01	1.00E+00
	Parkinson disease (P00049)	101	3	0.92	+	3.25	6.85E-02	1.00E+00
	Axon guidance mediated by netrin (P00009)	35	1	0.32	+	3.13	2.79E-01	1.00E+00
	Heterotrimeric G-protein signaling pathway-Gq alpha and Go alpha mediated pathway (P00027)	125	3	1.14	+	2.63	1.10E-01	1.00E+00
	Cytoskeletal regulation by Rho GTPase (P00016)	86	2	0.79	+	2.55	1.89E-01	1.00E+00
	Notch signaling pathway (P00045)	45	1	0.41	+	2.43	3.42E-01	1.00E+00
	Blood coagulation (P00011)	46	1	0.42	+	2.38	3.48E-01	1.00E+00
	Heterotrimeric G-protein signaling pathway-Gi alpha and Gs alpha mediated pathway (P00026)	163	3	1.49	+	2.02	1.91E-01	1.00E+00
	PI3 kinase pathway (P00048)	57	1	0.52	+	1.92	4.10E-01	1.00E+00
	EGF receptor signaling pathway (P00018)	142	2	1.3	+	1.54	3.75E-01	1.00E+00
	B cell activation (P00010)	71	1	0.65	+	1.54	4.81E-01	1.00E+00
	PDGF signaling pathway (P00047)	145	2	1.32	+	1.51	3.85E-01	1.00E+00
	Huntington disease (P00029)	152	2	1.39	+	1.44	4.07E-01	1.00E+00
	Interleukin signaling pathway (P00036)	89	1	0.81	+	1.23	5.59E-01	1.00E+00
	T cell activation (P00053)	92	1	0.84	+	1.19	5.71E-01	1.00E+00
	TGF-beta signaling pathway (P00052)	102	1	0.93	+	1.07	6.09E-01	1.00E+00
BMI-C2	JAK/STAT signaling pathway (P00038)	17	7	0.39	+	17.74	7.34E-07	1.17E-04
	Leucine biosynthesis (P02749)	3	1	0.07	+	14.36	8.77E-02	6.38E-01
	5-Hydroxytryptamine biosynthesis (P04371)	3	1	0.07	+	14.36	8.77E-02	6.10E-01

	Isoleucine biosynthesis (P02748)	4	1	0.09	+	10.77	1.08E-01	6.94E-01
	Tetrahydrofolate biosynthesis (P02742)	5	1	0.12	+	8.61	1.29E-01	7.62E-01
	Pyrimidine Metabolism (P02771)	11	2	0.26	+	7.83	3.40E-02	4.53E-01
	Fructose galactose metabolism (P02744)	11	2	0.26	+	7.83	3.40E-02	4.18E-01
	Mannose metabolism (P02752)	6	1	0.14	+	7.18	1.48E-01	8.48E-01
	Interferon-gamma signaling pathway (P00035)	30	4	0.7	+	5.74	7.08E-03	2.27E-01
	Cell cycle (P00013)	23	3	0.53	+	5.62	2.05E-02	4.10E-01
	Pentose phosphate pathway (P02762)	9	1	0.21	+	4.79	2.05E-01	9.65E-01
	Circadian clock system (P00015)	10	1	0.23	+	4.31	2.23E-01	9.92E-01
	Axon guidance mediated by semaphorins (P00007)	21	2	0.49	+	4.1	9.51E-02	6.34E-01
	Ras Pathway (P04393)	76	7	1.76	+	3.97	2.79E-03	1.49E-01
	PI3 kinase pathway (P00048)	57	5	1.32	+	3.78	1.32E-02	3.02E-01
	EGF receptor signaling pathway (P00018)	142	12	3.3	+	3.64	2.12E-04	1.69E-02
	Opioid proopiomelanocortin pathway (P05917)	36	3	0.84	+	3.59	5.81E-02	5.81E-01
	5HT1 type receptor mediated signaling pathway (P04373)	48	4	1.11	+	3.59	3.01E-02	4.38E-01
	Opioid proenkephalin pathway (P05915)	37	3	0.86	+	3.49	6.18E-02	4.95E-01
	Interleukin signaling pathway (P00036)	89	7	2.07	+	3.39	6.24E-03	2.49E-01
	Dopamine receptor mediated signaling pathway (P05912)	58	4	1.35	+	2.97	5.21E-02	5.56E-01
	Toll receptor signaling pathway (P00054)	61	4	1.42	+	2.82	6.01E-02	5.66E-01
	Insulin/IGF pathway-mitogen activated protein kinase kinase/MAP kinase cascade (P00032)	33	2	0.77	+	2.61	1.88E-01	9.71E-01
	p53 pathway feedback loops 2 (P04398)	51	3	1.18	+	2.53	1.24E-01	7.62E-01
	5HT3 type receptor mediated signaling pathway (P04375)	17	1	0.39	+	2.53	3.39E-01	1.00E+00

	CCKR signaling map (P06959)	173	10	4.02	+	2.49	9.17E-03	2.44E-01
	Nicotine pharmacodynamics pathway (P06587)	35	2	0.81	+	2.46	2.05E-01	1.00E+00
	Opioid prodynorphin pathway (P05916)	35	2	0.81	+	2.46	2.05E-01	9.93E-01
	PDGF signaling pathway (P00047)	145	8	3.37	+	2.38	2.37E-02	4.21E-01
	T cell activation (P00053)	92	5	2.14	+	2.34	6.99E-02	5.33E-01
	Angiotensin II-stimulated signaling through G proteins and beta-arrestin (P05911)	37	2	0.86	+	2.33	2.21E-01	1.00E+00
	Enkephalin release (P05913)	38	2	0.88	+	2.27	2.30E-01	9.43E-01
	Ubiquitin proteasome pathway (P00060)	58	3	1.35	+	2.23	1.61E-01	8.87E-01
	Glycolysis (P00024)	20	1	0.46	+	2.15	3.83E-01	1.00E+00
	Angiogenesis (P00005)	181	9	4.2	+	2.14	4.23E-02	4.84E-01
	p38 MAPK pathway (P05918)	41	2	0.95	+	2.1	2.55E-01	1.00E+00
	Gonadotropin-releasing hormone receptor pathway (P06664)	237	11	5.5	+	2	3.00E-02	4.80E-01
	p53 pathway by glucose deprivation (P04397)	23	1	0.53	+	1.87	4.24E-01	1.00E+00
	Blood coagulation (P00011)	46	2	1.07	+	1.87	2.97E-01	1.00E+00
	Inflammation mediated by chemokine and cytokine signaling pathway (P00031)	261	11	6.06	+	1.82	6.08E-02	5.40E-01
	Metabotropic glutamate receptor group II pathway (P00040)	48	2	1.11	+	1.79	3.14E-01	1.00E+00
	Apoptosis signaling pathway (P00006)	125	5	2.9	+	1.72	2.24E-01	9.68E-01
	FGF signaling pathway (P00021)	127	5	2.95	+	1.7	2.29E-01	9.65E-01
	Endothelin signaling pathway (P00019)	85	3	1.97	+	1.52	4.55E-01	1.00E+00
	Adrenaline and noradrenaline biosynthesis (P00001)	29	1	0.67	+	1.49	4.98E-01	1.00E+00
	Hypoxia response via HIF activation	29	1	0.67	+	1.49	4.98E-01	1.00E+00

	(P00030)							
	p53 pathway (P00059)	89	3	2.07	+	1.45	4.68E-01	1.00E+00
	Muscarinic acetylcholine receptor 2 and 4 signaling pathway (P00043)	61	2	1.42	+	1.41	6.55E-01	1.00E+00
	Heterotrimeric G-protein signaling pathway-Gi alpha and Gs alpha mediated pathway (P00026)	163	5	3.78	+	1.32	4.37E-01	1.00E+00
	Cadherin signaling pathway (P00012)	166	5	3.85	+	1.3	4.45E-01	1.00E+00
	Integrin signalling pathway (P00034)	200	6	4.64	+	1.29	4.77E-01	1.00E+00
	5HT2 type receptor mediated signaling pathway (P04374)	67	2	1.56	+	1.29	6.71E-01	1.00E+00
	Parkinson disease (P00049)	101	3	2.34	+	1.28	5.12E-01	1.00E+00
	VEGF signaling pathway (P00056)	68	2	1.58	+	1.27	6.74E-01	1.00E+00
	TGF-beta signaling pathway (P00052)	102	3	2.37	+	1.27	5.16E-01	1.00E+00
	5HT4 type receptor mediated signaling pathway (P04376)	34	1	0.79	+	1.27	5.52E-01	1.00E+00
	FAS signaling pathway (P00020)	34	1	0.79	+	1.27	5.52E-01	1.00E+00
	Metabotropic glutamate receptor group III pathway (P00039)	70	2	1.63	+	1.23	6.80E-01	1.00E+00
	GABA-B receptor II signaling (P05731)	36	1	0.84	+	1.2	5.73E-01	1.00E+00
	Heterotrimeric G-protein signaling pathway-rod outer segment phototransduction (P00028)	37	1	0.86	+	1.16	5.82E-01	1.00E+00
	Heterotrimeric G-protein signaling pathway-Gq alpha and Go alpha mediated pathway (P00027)	125	3	2.9	+	1.03	7.68E-01	1.00E+00
BMI-C3	Salvage pyrimidine ribonucleotides (P02775)	13	1	0.07	+	13.89	7.44E-02	1.00E+00
	De novo pyrimidine ribonucleotides biosythesis (P02740)	14	1	0.08	+	12.9	7.95E-02	1.00E+00
	De novo pyrimidine deoxyribonucleotide biosynthesis	15	1	0.08	+	12.04	8.46E-02	1.00E+00

	(P02739)							
	Glycolysis (P00024)	20	1	0.11	+	9.03	1.10E-01	1.00E+00
	Axon guidance mediated by semaphorins (P00007)	21	1	0.12	+	8.6	1.14E-01	1.00E+00
	p53 pathway by glucose deprivation (P04397)	23	1	0.13	+	7.85	1.24E-01	1.00E+00
	Hypoxia response via HIF activation (P00030)	29	1	0.16	+	6.23	1.53E-01	1.00E+00
	De novo purine biosynthesis (P02738)	31	1	0.17	+	5.83	1.62E-01	1.00E+00
	Insulin/IGF pathway-mitogen activated protein kinase kinase/MAP kinase cascade (P00032)	33	1	0.18	+	5.47	1.71E-01	1.00E+00
	VEGF signaling pathway (P00056)	68	2	0.38	+	5.31	5.70E-02	1.00E+00
	Axon guidance mediated by netrin (P00009)	35	1	0.19	+	5.16	1.80E-01	1.00E+00
	p38 MAPK pathway (P05918)	41	1	0.23	+	4.41	2.07E-01	1.00E+00
	Heterotrimeric G-protein signaling pathway-Gq alpha and Go alpha mediated pathway (P00027)	125	3	0.69	+	4.33	3.38E-02	1.00E+00
	Notch signaling pathway (P00045)	45	1	0.25	+	4.01	2.25E-01	1.00E+00
	Heterotrimeric G-protein signaling pathway-Gi alpha and Gs alpha mediated pathway (P00026)	163	3	0.9	+	3.32	6.40E-02	1.00E+00
	Angiogenesis (P00005)	181	3	1	+	2.99	8.14E-02	1.00E+00
	PDGF signaling pathway (P00047)	145	2	0.8	+	2.49	1.94E-01	1.00E+00
	Cadherin signaling pathway (P00012)	166	2	0.92	+	2.18	2.37E-01	1.00E+00
	Cytoskeletal regulation by Rho GTPase (P00016)	86	1	0.48	+	2.1	3.82E-01	1.00E+00
	p53 pathway (P00059)	89	1	0.49	+	2.03	3.92E-01	1.00E+00
	Interleukin signaling pathway (P00036)	89	1	0.49	+	2.03	3.92E-01	1.00E+00
	Wnt signaling pathway (P00057)	314	3	1.74	+	1.73	2.54E-01	1.00E+00
	FGF signaling pathway (P00021)	127	1	0.7	+	1.42	5.08E-01	1.00E+00

Figure S1: Study design.

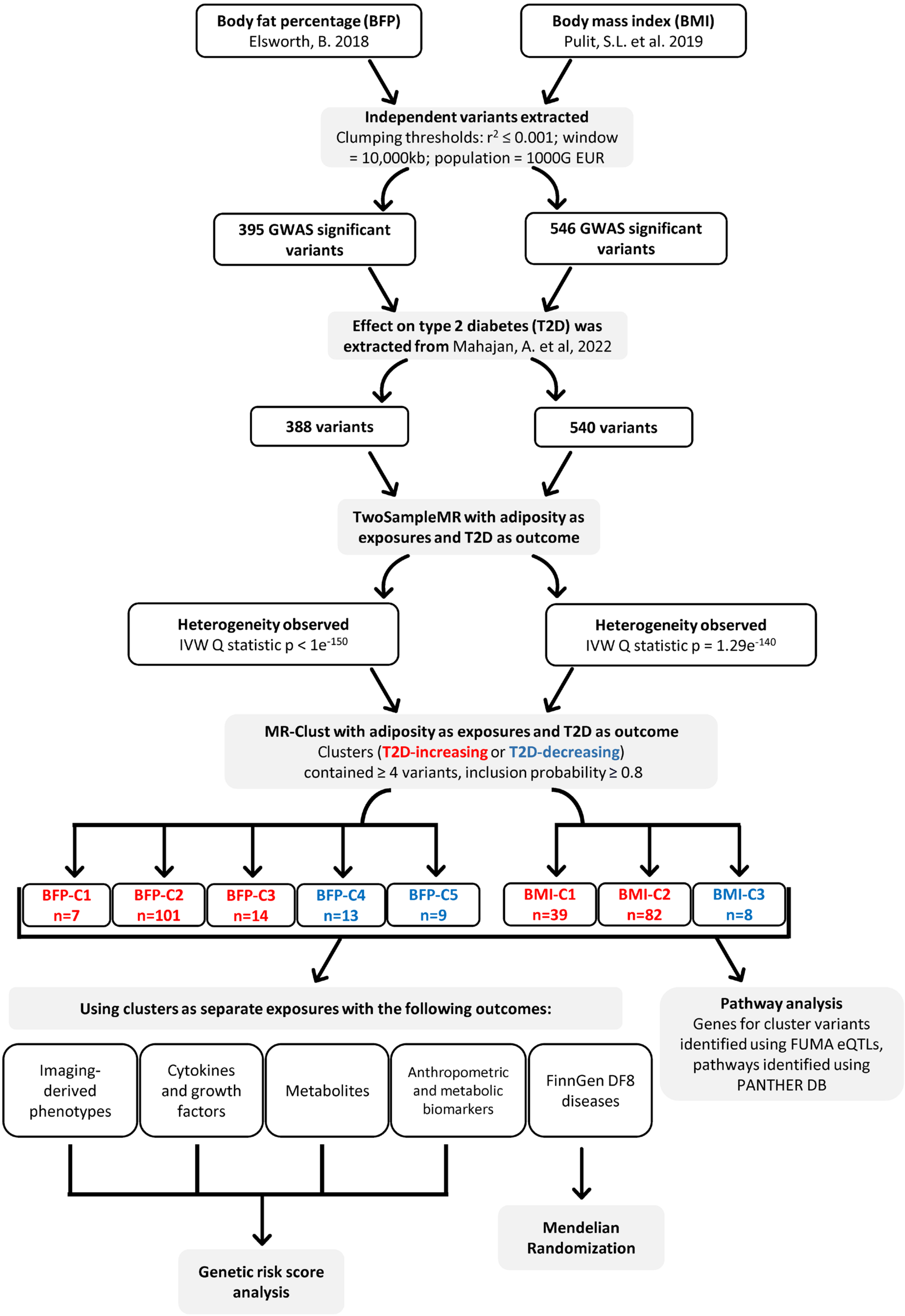

Figure S2: Scatter plots of genetic association between adiposity and type 2 diabetes. The x-axis is the genetic association between **(A)** body fat percentage (BFP) or **(B)** Body mass index (BMI), and the y-axis is the genetic association with type 2 diabetes. Each dot represents a genetic variant associated with (A) BFP (n=388) or (B) BMI (n=540). Error bars represent 95% confidence intervals for the genetic associations.

(A)

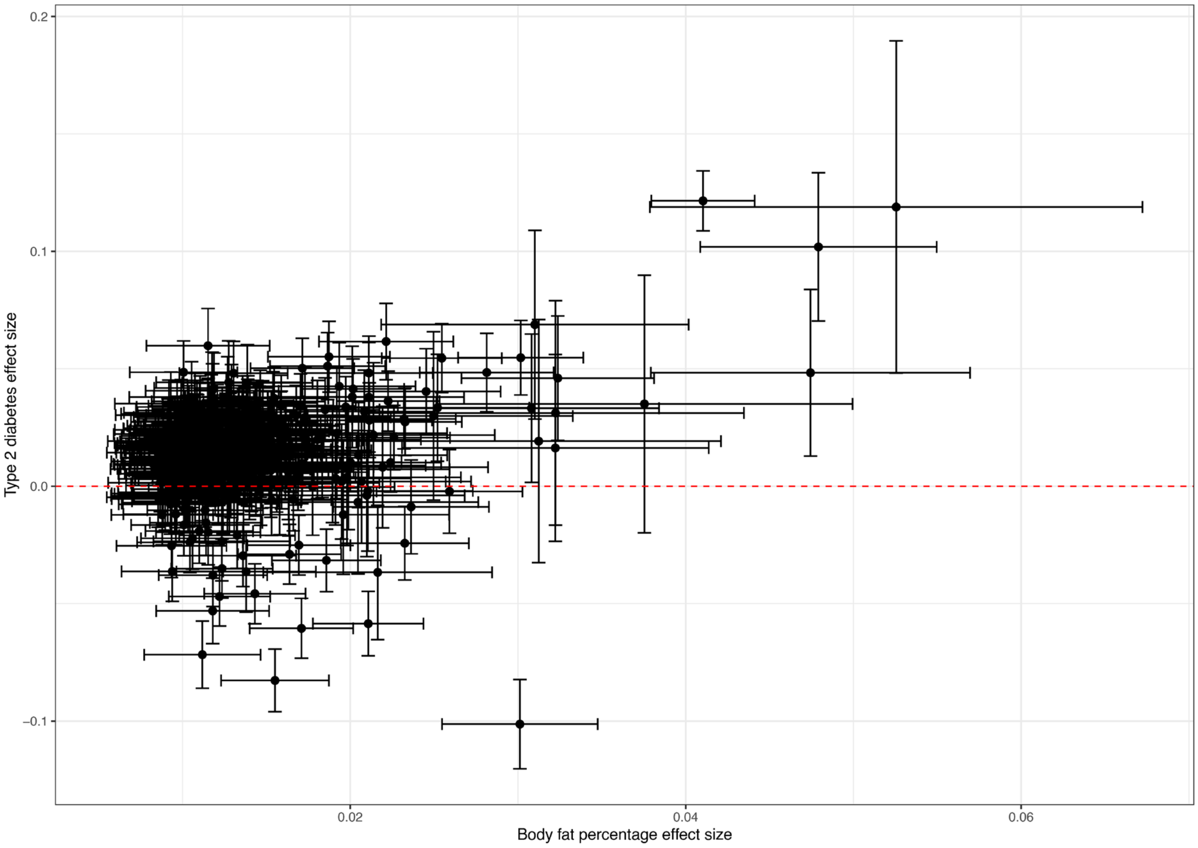

(B)

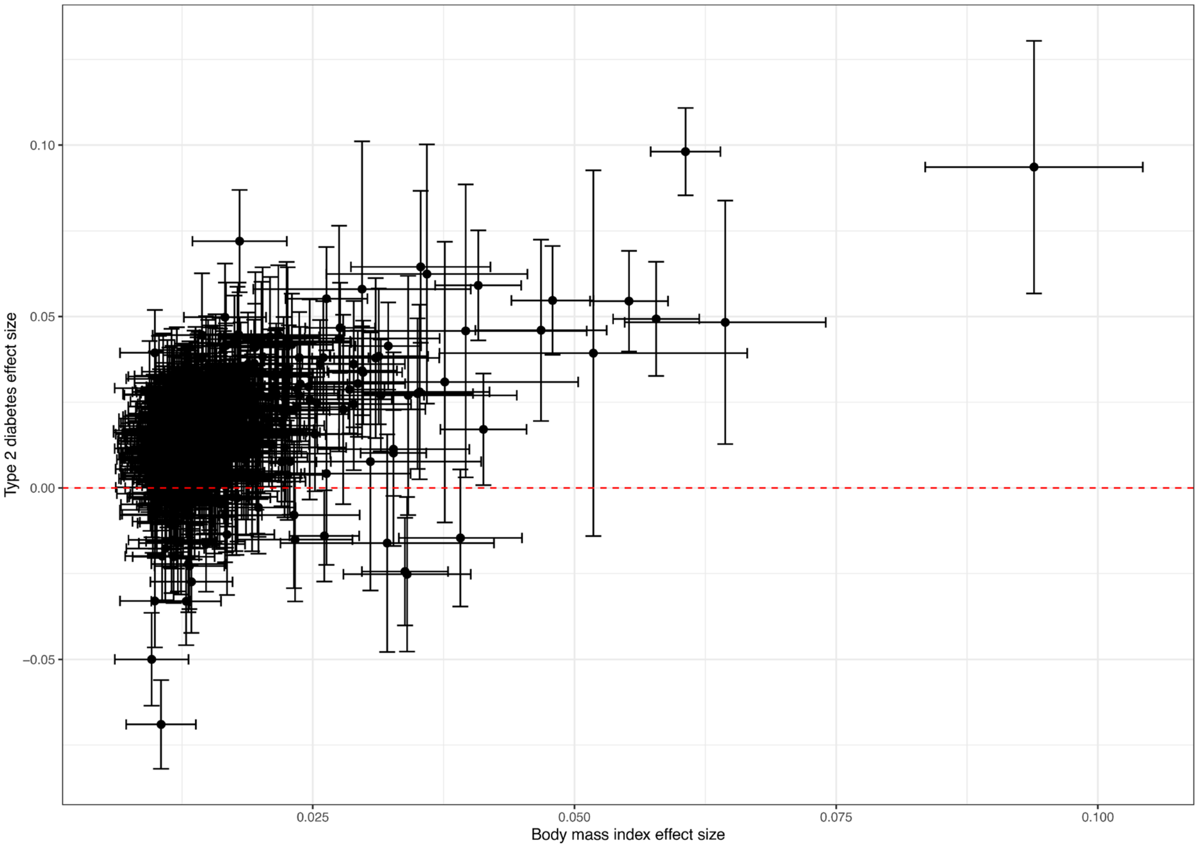

Type 2 diabetes effect size
0.10
0.05
0.00
−0.05
0.025
0.050
0.075
0.100
Body mass index effect size

Figure S3: Venn diagram showing number of variants either correlated with or shared between adiposity-increasing clusters. Correlation between all cluster variants were investigated using $r^2 \geq 0.8$. There was correlation amongst unfavorable adiposity clusters (BFP-C1: 7 variants, BMI-C1: 39 variants, BFP-C2: 101 variants, BMI-C2: 82 variants, BFP-C3: 14 variants) and amongst favorable adiposity clusters (BFP-C4: 13 variants and BMI-C3: 8 variants. BFP-C5: 9 variants did not have any correlated or shared variants with any other cluster. There was no correlation between unfavorable and favorable adiposity clusters.

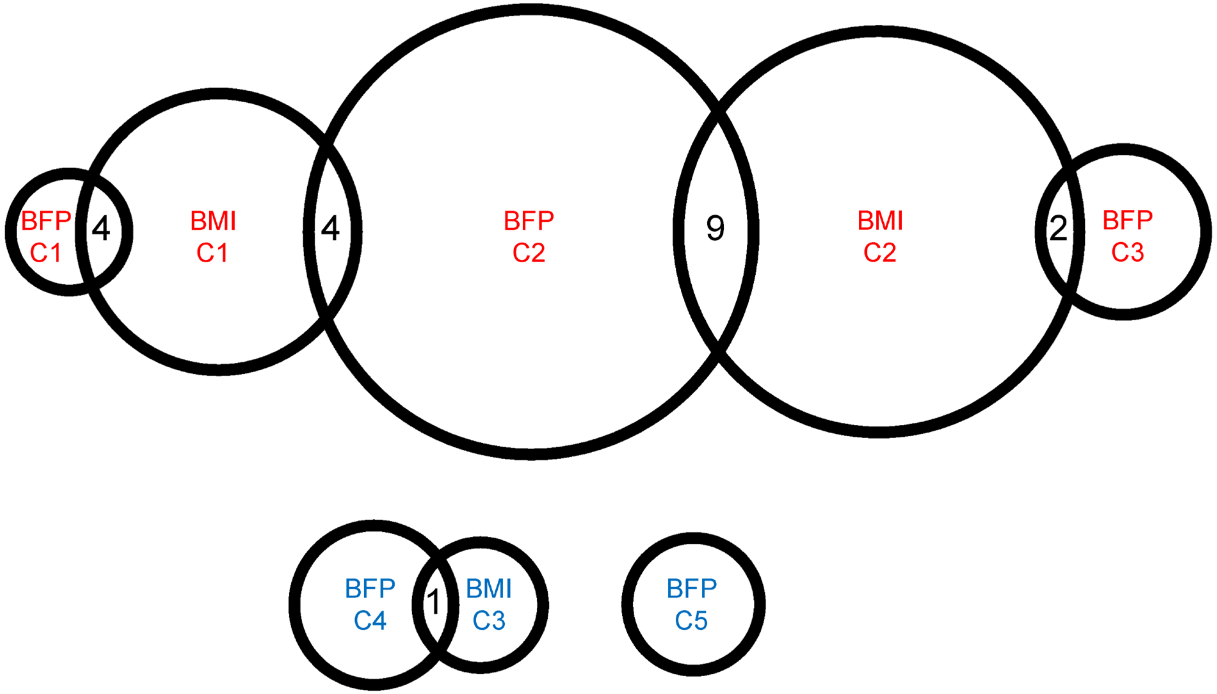

Supplement: Supplementary material [file EMS195997-supplement-Supplementary_material.pdf]
